# Identification of β-strand mediated protein–protein interaction inhibitors using ligand-directed fragment ligation†

**DOI:** 10.1039/d0sc05694d

**Published:** 2021-01-06

**Authors:** Zsófia Hegedüs, Fruzsina Hóbor, Deborah K. Shoemark, Sergio Celis, Lu-Yun Lian, Chi H. Trinh, Richard B. Sessions, Thomas A. Edwards, Andrew J. Wilson

**Affiliations:** School of Chemistry, University of Leeds Woodhouse Lane Leeds LS2 9JT UK a.j.wilson@leeds.ac.uk; Astbury Centre for Structural Molecular Biology, University of Leeds Woodhouse Lane Leeds LS2 9JT UK; School of Molecular and Cellular Biology, University of Leeds Woodhouse Lane Leeds LS2 9JT UK; Institute of Systems, Molecular and Integrative Biology, University of Liverpool Liverpool L69 3BX UK; School of Biochemistry, Biomedical Sciences Building, University of Bristol Bristol BS8 1TD UK

## Abstract

β-Strand mediated protein–protein interactions (PPIs) represent underexploited targets for chemical probe development despite representing a significant proportion of known and therapeutically relevant PPI targets. β-Strand mimicry is challenging given that both amino acid side-chains and backbone hydrogen-bonds are typically required for molecular recognition, yet these are oriented along perpendicular vectors. This paper describes an alternative approach, using GKAP/SHANK1 PDZ as a model and dynamic ligation screening to identify small-molecule replacements for tranches of peptide sequence. A peptide truncation of GKAP functionalized at the N- and C-termini with acylhydrazone groups was used as an anchor. Reversible acylhydrazone bond exchange with a library of aldehyde fragments in the presence of the protein as template and *in situ* screening using a fluorescence anisotropy (FA) assay identified peptide hybrid hits with comparable affinity to the GKAP peptide binding sequence. Identified hits were validated using FA, ITC, NMR and X-ray crystallography to confirm selective inhibition of the target PDZ-mediated PPI and mode of binding. These analyses together with molecular dynamics simulations demonstrated the ligands make transient interactions with an unoccupied basic patch through electrostatic interactions, establishing proof-of-concept that this unbiased approach to ligand discovery represents a powerful addition to the armory of tools that can be used to identify PPI modulators.

## Abstract 2

Dynamic ligation screening is used to identify acylhydrazone-linked peptide-fragment hybrids which bind to the SHANK1 PDZ domain with comparable affinity to the native GKAP peptide as shown by biophysical and structural analyses.

## Graphical abstract

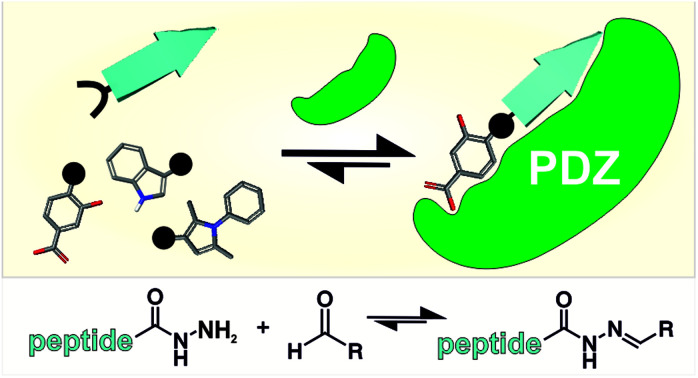

## Introduction

Protein–protein interactions (PPIs) play an essential role in the majority of biological processes and therefore represent arbiters of health and disease.^1,2^ Chemical probes for PPIs offer tremendous opportunities to understand PPIs and therefore biological mechanisms, whilst providing starting points for drug-discovery.^3^ Conventional ligand discovery methods (*e.g.* fragment screening and elaboration) have delivered PPI modulators for a number of targets^4–6^ leading to clinically approved drugs such as Venetoclax (ABT-199).^7^ However, despite the enormous opportunity offered by PPIs, methods to identify modulators of PPIs remain underdeveloped. Peptide interacting motifs represent promising templates for design given they are likely to mediate a significant proportion of PPIs;^8^ however, selecting the most appropriate scaffold to inhibit a specific PPI is highly dependent on the properties of the target interaction and varies in difficulty across PPI classes.^9,10^ There has been considerable success in developing generic approaches to mimic the α-helix and inhibit α-helix mediated PPIs (*e.g.* using peptides, constrained peptides and peptidomimetics), but less so for other PPI topographies.^11,12^ In particular, β-strand/β-sheet mediated PPIs exhibit a more complex topography and have proven more challenging; these interfaces are shallower and more elongated with backbone hydrogen bonding contributing significantly to the binding energy.^13,14^ Scaffolds that mimic β-strands/β-sheets^15–20^ have hitherto seen limited application^21–23^ in PPI inhibitor discovery.^24–29^

**Fig. 1:**
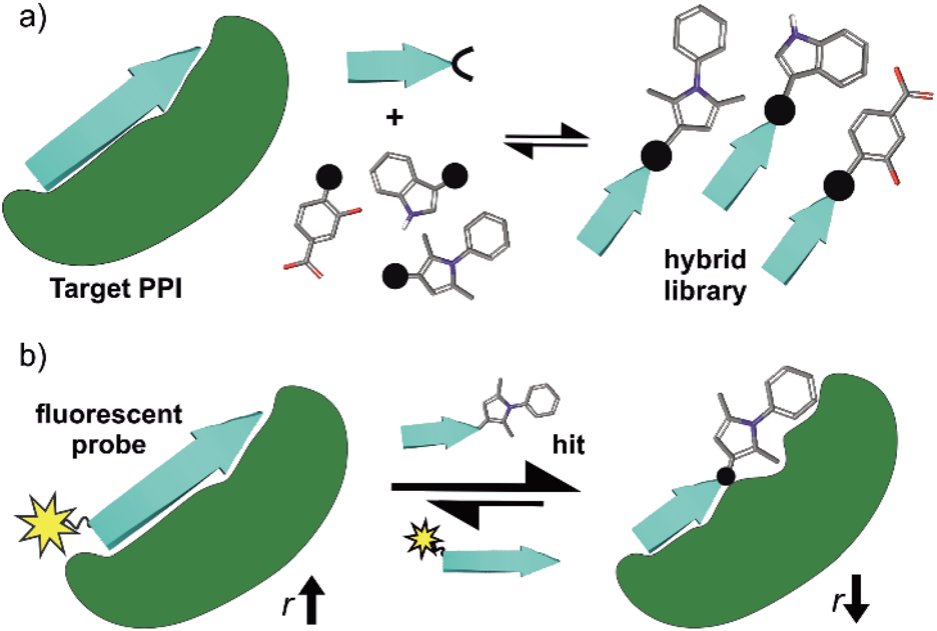
(a) Design and screening strategy for identification of hybrid inhibitors. Schematic depicting the design strategy for identification of small molecule-peptide hybrids using a truncated anchor peptide derived from a known interaction partner of the protein target, and (b) Schematic depicting fluorescence anisotropy (*r*) competition assay-based screen for the identification of hit compounds that compete with a fluorescently labelled ligand at the target binding site.

In this work, we take a novel approach to β-strand mimicry whereby a truncated protein-interacting motif is combined with a small-molecule fragment (Fig. 1a), to generate a functional mimetic,^30^*i.e.* a mimic that reproduces binding, but may not necessarily mimic structure. As a screening approach, this offers the advantage of exploiting a peptide with intrinsic target affinity as an anchor to permit identification of weakly binding fragments. A related principle has been exploited in the REPLACE strategy whereby – aided by *in silico* methods – key residues of known peptide ligands were “mutated” for molecular fragments to identify inhibitors of conventional drug discovery targets *e.g.* kinases.^31–35^ Subsequently, extension of this concept to α-helix mediated PPIs has been achieved by screening peptide-small molecule hybrids *in silico*, then preparing promising candidates for experimental validation using click chemistry.^36–38^ In this work, we instead use reversible hydrazone formation^39^ between peptide hydrazones and an aldehyde library together with dynamic ligation screening^40,41^ to identify peptide-fragment hybrids. Such dynamic, template assisted methods shift thermodynamic equilibria in favour of the highest affinity ligands^40–44^ and can target unoccupied binding sites^45^ in an unbiased manner; they have proven successful for discovery of active site inhibitors,^46–49^ but not been applied to PPI inhibitor discovery.

As a model β-strand mediated PPI, we use the GKAP/SHANK-PDZ^50^ which, plays a role in the organization of synaptic protein complexes and has been linked to several neuronal disorders.^51^ There are >250 PDZ domains with several therapeutically relevant proteins known to exhibit their function through a PDZ mediated interaction.^52–54^ Ligand discovery for PDZ domains has proven challenging (*e.g.* fragment-based screening was unsuccessful for the PDZ domain of PSD-95 ^55^), with the majority of potent ligands based on protein,^56,57^ peptide^58,59^ or peptidomimetic scaffolds^27,60–62^ and limited examples of small-molecules.^63–66^ This rendered the GKAP/SHANK-PDZ interaction as a stringent test for our dynamic ligation screening approach. Using a three-residue sequence from the GKAP ligand, C- and N-terminal hydrazones were generated, then peptide-fragment hybrid assembly under neutral conditions performed in the presence of protein as a template and directly coupled to a fluorescence-based biophysical assay (Fig. 1b). Characterization of the most promising hits using ITC, NMR, X-ray crystallography and molecular dynamics simulations established the hits as selective SHANK1 PDZ ligands (in comparison to PSD-95) with comparable potency to the GKAP PDZ-binding motif (PBM) (Ac-EAQTRL-COOH) from which they were derived. Potency was achieved by binding to a cluster of basic residues proximal to the peptide binding site on the PDZ domain. These results thus establish dynamic ligation screening as a powerful tool to identify β-strand peptide-fragment mimetics as PPI inhibitors and broaden the scope of design strategies for PPI inhibitor discovery.

## Results and discussion

### Hybrid library design

**Fig. 2:**
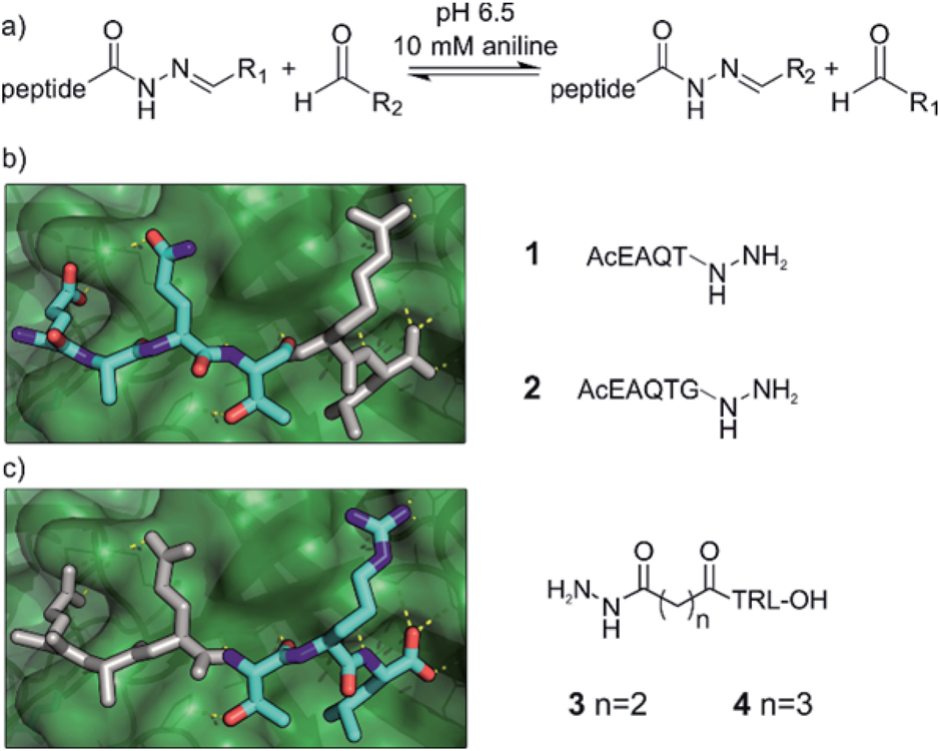
Design principles for the anchor peptides based on the GKAP/SHANK1 PDZ interaction. (a) General reaction scheme for acylhydrazone exchange using peptide hydrazones and aldehydes in the presence of 10 mM aniline at pH 6.5. Anchor peptides generated from the GKAP sequence (cyan) based on its binding properties to SHANK1 PDZ (green, 1Q3P), truncated either from (b) the C terminus or (c) the N terminus. Residues outlined in white indicate the truncated amino acids and the fragment-targeting binding site.

We chose acylhydrazone bond formation to covalently link fragments to peptide anchors (Fig. 2a). In the presence of anilines, the reaction can be performed close to neutral pH, allowing the screening to take place close to a physiologically relevant pH and temperature with the protein as a template for product formation.^67,68^ Moreover, at neutral pH acylhydrazones are stable, facilitating subsequent hit validation.^69,70^ Given the commercial availability of a large array of aldehydes, we thus designed the hybrids to be formed from peptide hydrazides.

Peptide anchors were designed based on the key features of the interaction between the wild type GKAP sequence and SHANK1 PDZ.^50^ Class I PDZ domains bind to consensus sequences with a Thr/Ser in the -2 position from the C-terminus, with the C-terminus hydrophobic residue and free carboxylate being the main determinants of affinity.^71,72^ The plasticity of PDZ domains allows the accommodation of various hydrophobic side-chains at the C terminus of the peptide,^73^ which we hypothesized to be an ideal target site for hydrophobic fragments; for SHANK1, Leu dominates for C-terminal carboxylates and Phe for non-C-terminal sequences.^74^ This led to compounds **1** and **2** (Fig. 2b and Scheme S1†), directing the fragments toward the C-terminal hydrophobic pocket on SHANK1 PDZ. On the other hand, compounds **3** and **4** contained the TRL-COOH core sequence and the hydrazide functionality was attached to the N-terminus of the peptide through a 2 or 3 carbon atom linker (Fig. 2c and Scheme S2†), allowing exploration of the protein surface for secondary binding sites further away from the key residues. To ease purification, all peptide hydrazides were reacted with benzaldehyde, yielding the corresponding acylhydrazones (**1-**, **2-**, **3-**, **4-A001**).

A diversity-based selection was performed on commercially available aldehyde fragments obeying guidelines for fragment-based drug design,^75^ resulting in a small library of 129 compounds (**A001–A129**, Table S1 and Fig. S1†). Acylhydrazone formation was tested in the presence of 10 mM aniline^67^ at pH 6.5 using a 5-fold excess of the competing aldehydes, which revealed fast and complete exchange of the benzaldehyde motif (Fig. S2†), reaching equilibrium within 24 hours. To allow us to perform the hybrid formation and screening in the presence of the target protein in a single step, it was necessary to first establish that SHANK1 PDZ was still able to bind to its natural ligand under the same conditions. For this, FITC labelled GKAP was used and the fluorescence anisotropy measurements gave an initial *K*_D_ of ∼1 μM, which did not significantly change over the course of 24 hours (Fig. S3†).

### Library screening

**Fig. 3:**
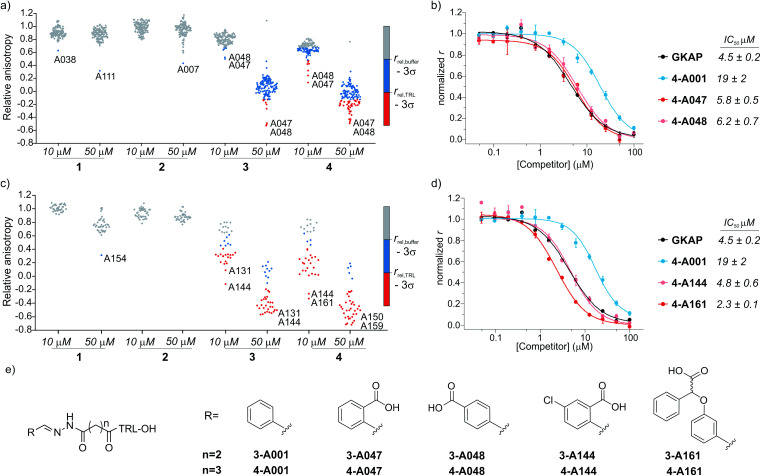
Results of the fluorescence anisotropy competition screening for compounds **1–4**. (a) Screening results for hydrazones formed from **1–4** using the diverse screening library (A001-A129). (b) FA competition curves of the best hybrid hits. (c) Screening results using the extended library including near neighbors (A130-165) for the initial hits. (d) FA competition curves for hybrids from the extended screen. All screening and validation assays were performed in 50 mM NH_4_Ac buffer, pH 6.5, 10 mM aniline using 1 μM final protein concentration and 10 nM FITC-Ahx-TRL-COOH as tracer at room temperature. Compounds **1-**, **2-**, **3-**, **4-A001** were incubated individually with 5 equivalents of fragments in the presence of SHANK1 PDZ for 24 hours. Anisotropy is expressed relative to the control experiment where no competitor is present. Hit thresholds were defined based on the relative anisotropy of the (i) buffer control (*r*_buffer_ − 3*σ*) and (ii) the TRL control (*r*_TRL_ − 3*σ*). Compounds below these thresholds are coloured blue and red respectively. Labels indicate the two best aldehyde fragment hits from the individual screens. For individual values and FA validation for additional hits, see ESI Fig. S5–S12.† (e) Structures of the synthesized hit compounds. The absolute configuration and enantiomeric purity of fragment A161 is unknown.

Prior to screening, the intrinsic inhibitory potency of the peptide hydrazones **1-**, **2-**, **3-**, **4-A001** was determined (Fig. S4†); they were no more active than the parent peptides. Then, hybrid formation and screening were performed by mixing each of these compounds at 10 or 50 μM concentration with 5 equivalents of the aldehydes in a 384 well microtiter plate. After 24 hours of equilibration in the presence of SHANK1 PDZ protein, FITC-Ahx-TRL-OH was added as competitor. In all assays, the GKAP PBM sequence (Ac-EAQTRL-COOH), Ac-TRL-OH and a buffer control were used, which facilitated determination of threshold values for the hit compounds (Fig. 3a, S5 and S6†). Any hybrid that exceeded the activity of its parent compound was considered an improvement caused by the replacement of the fragment.

The two different truncation strategies had profoundly different hit rates, reflecting the affinity of the parent compounds. The removal of the key C terminal residue of GKAP did not result in detectable binding affinity for the parent compounds **1-A001** and **2-A001** (Fig. S4†); our hypothesis was that a hybrid with a fragment effectively mimicking the truncated sequence could result in detectable affinity. Our library did not contain such fragments; hit rates were low, perhaps reflecting the challenge in attaining the correct orientation and composition of functionality needed to faithfully mimic the key interactions afforded by the natural truncated sequence. The solitary confirmed hit (Fig. S7†) appeared to interfere with the assay and was not considered further.

Conversely, using compounds **3-A001** and **4-A001** with intrinsic potency (Fig. S4†) resulted in a higher hit rate (Fig. 3a, S5 and S6†), with 51 hybrids exceeding the activity of Ac-TRL-COOH, which was the core motif in both hybrid sequences. The most promising hits were re-tested in order to validate the screening assay (Fig. 3b, S8 and S9†). The IC_50_ values measured for the best hits were in the range of 5–6 μM, a 10-fold improvement compared to the parent Ac-TRL-COOH sequence, indicating that covalent attachment of the fragment contributes favourably to interaction with the protein. Interestingly, the most potent hybrids formed from **3** and **4** contained the same carboxylic acid functionalized aromatic aldehyde fragments (A047 and A048, Fig. 3b), indicating that these types of fragments might play an important role in binding.

Based on this observation, we extended the aldehyde library to near neighbours of these fragments and selected an additional 36 carboxylic acid functionalized aromatic aldehydes (A130-165, Fig. S1 and Table S1†) with which to carry out a further screen. In this extended screen, the hit rate increased significantly (Fig. 3c, d and S10–S12†), confirming that an acidic binding motif is generic for favourable binding and confirming they could not have been identified without conjugation to the anchor peptides.

In the subsequent validation, the IC_50_ values of the best hits (formed from compound **4** and **A144** or **A161**) reached or slightly exceeded the binding affinity of full length GKAP peptide, indicating that the fragment can restore the affinity of the truncated sequence to that of the wild-type peptide. Significantly, all but one of the aldehyde fragments showed no inhibitory capability on their own in the assay (Fig. S13†).

### Structure–activity relationships

**Fig. 4:**
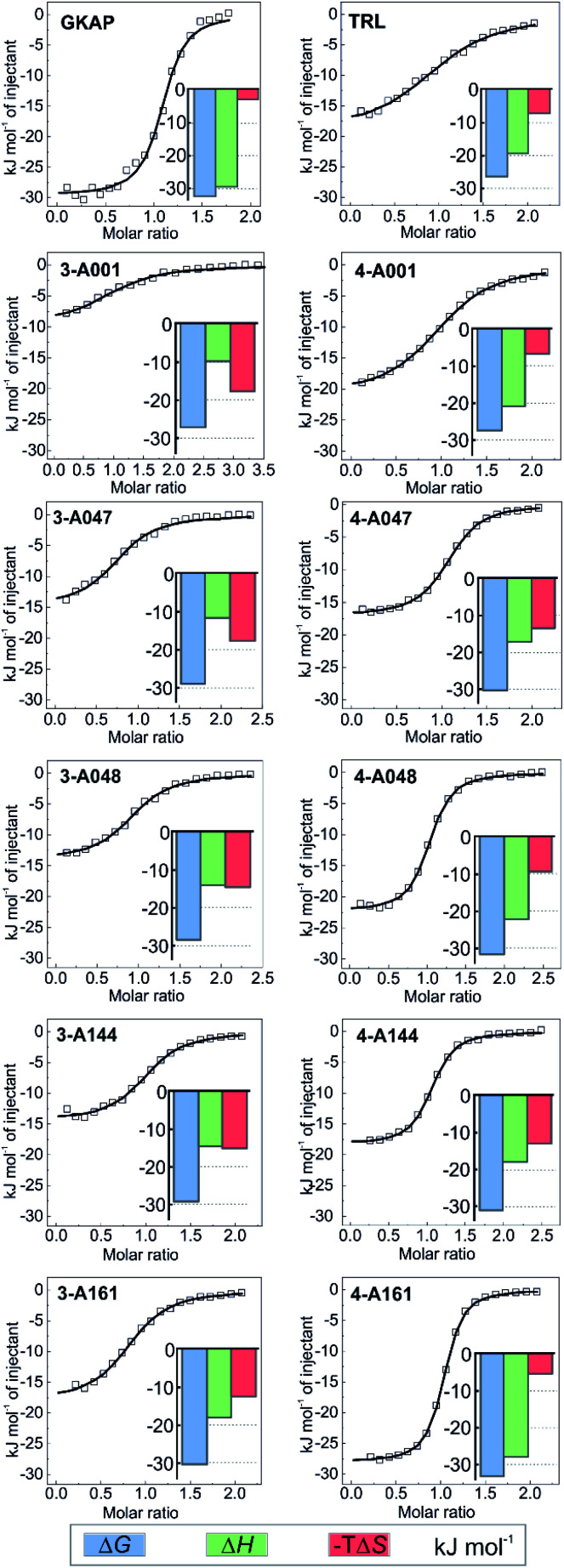
Hit validation by ITC. Fitted thermograms and thermodynamic signatures for the tested peptides and hydrazones. ITC data were acquired in 25 mM Tris, pH 7.5 buffer containing 150 mM NaCl at 25 °C by injecting 0.75–1 mM peptide or hydrazone solution into 75–150 μM protein in the cell. For individual raw ITC data and fitted thermograms see Fig. S14.†

To establish structure–activity relationships, the most active hybrids were synthesized individually (Fig. 3e and Scheme S2†) and subjected to further characterization by isothermal titration calorimetry (ITC) (see Fig. S14†). The truncation of the GKAP sequence (*K*_D_ = 2 ± 0.3 μM) to its core TRL motif (*K*_D_ = 22 ± 3 μM) resulted in a decrease in affinity (Fig. 4 and Table 1). This was accompanied by the loss of binding enthalpy but also resulted in a more favourable entropy of binding (Table 1), presumably due to the loss of rotatable bonds on amino acid removal. Hydrazones formed from benzaldehyde and this core motif (compounds **3-A001** and **4-A001**) revealed slightly increased affinity compared to the TRL motif (Fig. 4 and Table 1), which might be the result of favourable interaction of the aromatic fragment or the hydrazone bond itself with the protein. Hybrid hits in which the fragments contained the additional carboxylic acid functional group resulted in increased binding affinity in every case (Fig. 4 and Table 1), which confirmed the importance of the acidic group and that these fragments actively contribute to SHANK1 binding. Interestingly, hybrids with the C3 linker (compound **4** hydrazones) showed more favourable enthalpic contributions and slightly higher affinity in comparison to compounds having the C2 linker (compound **3**) when the same fragment was attached. This indicated that the more flexible linker allows for a better orientation of the fragment leading to a more favourable enthalpy of binding, but also increases the entropic cost of binding, leading only to a moderate difference in the overall binding energy.

**Table 1:** Thermodynamic parameters for the tested compounds binding to SHANK1 PDZ derived from the ITC experiments

	*n*	*K*_D_ (μM)	Δ*H* (kJ mol^−1^)	Δ*S* (J mol^−1^ K^−1^)
**GKAP**	1.07 ± 0.01	2.0 ± 0.3	−29.6 ± 0.4	9.7
**TRL**	1.04 ± 0.02	23 ± 2	−19.3 ± 0.5	24.1
**3-A001**	1.04 ± 0.04	16 ± 3	−9.7 ± 0.6	59.0
**4-A001**	1.00 ± 0.03	14.8 ± 0.8	−20.9 ± 0.2	22.2
**3-A047**	0.94 ± 0.01	8 ± 1	−11.5 ± 0.4	59.1
**4-A047**	1.06 ± 0.01	4.6 ± 0.2	−17.1 ± 0.1	44.8
**3-A048**	1.03 ± 0.01	10 ± 1	−14.1 ± 0.3	48.4
**4-A048**	0.99 ± 0.01	3.1 ± 0.2	−22.3 ± 0.2	30.7
**3-A144**	1.00 ± 0.02	7.1 ± 0.9	−14.4 ± 0.2	50.1
**4-A144**	1.03 ± 0.01	3.1 ± 0.2	−18.3 ± 0.1	44.1
**3-A161**	0.84 ± 0.01	4.8 ± 0.4	−18.0 ± 0.3	41.3
**4-A161**	1.00 ± 0.00	1.54 ± 0.05	−28.0 ± 0.1	17.3

Based on its affinity and the thermodynamic signature, compound **4-161** was identified as the best mimic of the wild-type sequence. It should be noted that the fragment in this hybrid has unknown enantiomeric purity, therefore the binding affinity might be the average of the two diastereoisomers. To compare the effect of fragments on binding energy we calculated group efficiency values (GE) (Tables S2–S4†). These data illustrate the potential of the dynamic ligation approach to rapidly explore structure affinity relationships to identify the efficient fragments. Moreover, the replacement of the N terminal EAQ amino acids of GKAP to completely non-peptidic motifs resulted in similar GE values indicating this strategy can also provide molecules that are less peptidic in nature, while keeping the original potency of the native ligand. It is worth noting that although compound **4-161** possessed the highest binding affinity, the aldehyde fragment in compounds **4-A048** or **4-A144** have slightly higher group efficiencies (Tables S2–S4†) and may therefore represent equally reasonable starting points for further optimization.

To obtain more insight on the molecular nature of the interaction between the peptide-fragment hybrids and SHANK1 PDZ, co-crystal structures were solved for compounds **3-A047**, **4-A047**, **3-A048**, and **4-A048** with SHANK1 PDZ at resolutions of 1.5–2.2 Å (Table S5†). In all structures, hybrids bound to the anticipated binding site with a similar conformation to the wild type sequence within the core amino acid sequence (RMSD values between 0.18–0.32, Fig. S15–S19 and Table S6†), indicating that the peptide component of the hybrid indeed fulfils the role of an anchor directing fragments towards the protein surface. In all hybrids, the hydrazone bond existed in an *E* configuration with the aromatic ring of the fragment co-planar with the hydrazone. The N terminal glutamate of the wild-type GKAP interacts with R679, thus we anticipated that the carboxylate of the hybrids might reproduce this same interaction. However, the N terminal part of the hybrids showed different binding behaviour in comparison with the wild type sequence. In some cases, we observed multiple conformations of the same compound having different orientations of the fragments relative to the peptide component presumably allowed by the conformational flexibility of the linker region. The more favourable entropy of binding (Table 1) supports the hypothesis that linker flexibility leads to a “fuzzier” mode^76^ of interaction (see below) although the carboxylate exhibited a slight preference for interaction with R736 (Fig. S19†) which differs from the wild-type GKAP peptide (see above) and likely arises from a combination of hybrid flexibility and the propensity of R736 to engage in effective charge-reinforced hydrogen-bonding interactions.

**Fig. 5:**
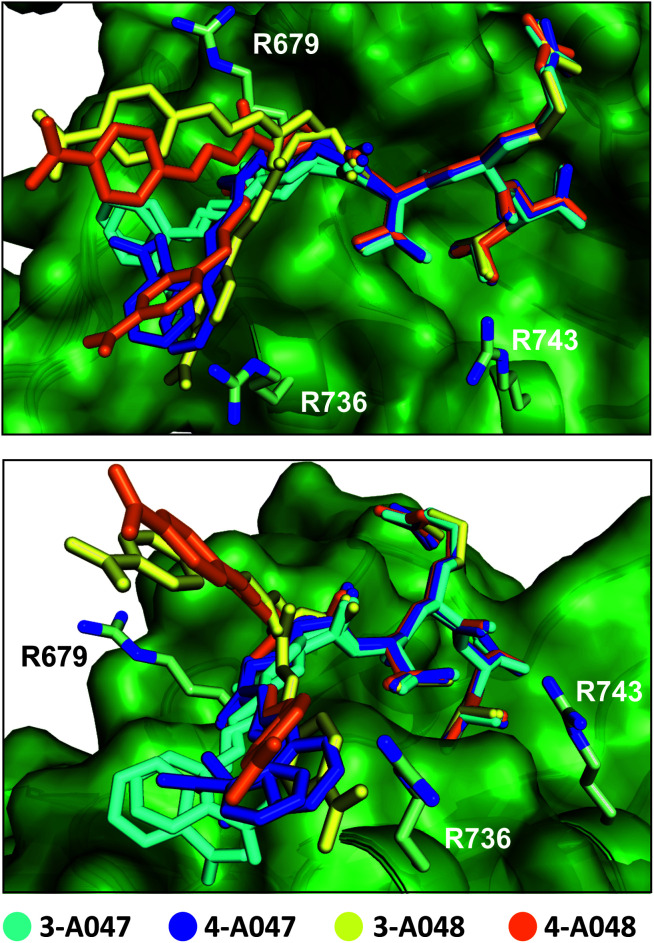
Superimposed crystal structure of **3-A047** (cyan, PDB: 6YWZ), **4-A047** (blue, PDB: 6YX1), **3-A048** (yellow, PDB: 6YX0) and **4-A048** (orange, PDB: 6YX2) in complex with SHANK1 PDZ (green) showing two different orientations of the complexes. The core TRL sequence binds in a similar manner to the native wild-type ligand in both crystallographic protomers whilst the orientation of the acylhydrazone fragment component varies for each ligand and appears to switch interactions dynamically with a series of basic residues (R679, R736, R743) at the periphery of the β-strand binding cleft on the PDZ domain.

It is noteworthy that the linker flexibility resulted in high B-factors with some missing density around the fragment in most of the structures. Although this leads to slight uncertainty about the exact location of the fragments, we hypothesize that the key carboxylic acid moiety moves dynamically across a positively charged patch formed by R679, R736 and R743 on the protein surface (Fig. 5), making transient charge-reinforced hydrogen-bonding and cation–π interactions which is also consistent with a more favourable entropy of interaction. Finally, in the crystal structures where strong electron density allowed determination of the location of the fragment, we observed that this motif interacted with a loop of a symmetry related protein in the crystals (Fig. S17 and S18†). To probe this behaviour further, we determined the rotational correlation time (*τ*_c_) of the SHANK1 PDZ protein in the absence/presence of ligand using NMR *T*_1_ and *T*_2_ relaxation experiments (Fig. S20†). These analyses indicated similar *τ*_c_ values for apo-SHANK1 and SHANK1 in the presence of these compounds, meaning that the interaction in the X-ray structure most likely arises from a crystal packing specific interaction.

To provide support for the hypothesis that the hybrids target the cationic patch on the surface of SHANK1, we performed molecular dynamics simulations on the SHANK1-compound **3-A048** complex using the crystal structure as a starting point. Over the course of three repeats of 200 ns, the aromatic carboxylate of the fragment indeed moves across the surface of the SHANK1 protein, making transient contacts with R679, R736 and R743, as well as intramolecular interaction onto the arginine in the peptide ligand to a lesser extent (see: ESI, Movie S1†). The distance between the carboxylate and these residues plotted against time over the course of the simulations (Fig. S21†) further indicates that the aromatic carboxylate is able to make transient non-covalent contacts in turn with each of the positively charged side-chains of the three arginine residues with a preference for R736.

Since Class I PDZ domains bind to consensus sequences,^71,72^ we set out to further assess the selectivity of the peptide-fragment hybrids against another Class I PDZ domain. We compared the ability to bind to SHANK1 PDZ against the ability to bind to the PDZ domain of PSD-95, a therapeutically important target in neuronal diseases and cancer.^58^ We monitored the binding using chemical shift perturbations in the ^1^H–^15^N HSQC spectra of the PDZ domain of PSD-95 (Fig. S22, S23, Tables S7 and S8†). These analyses indicated weak binding with EC_50_ = 2.4 ± 0.3 mM for **4-A047** (Fig. S24†), which is comparable with the affinity of GKAP towards PSD-95 (Table S7†). The observed weak potency of GKAP and the peptide-acylhydrazone fragment hybrids towards PSD-95 likely derives from its preference for Val or Ile at the C-terminus of its ligands.^77^ However, PSD-95 also lacks the patch of cationic residues proximal to the C-terminal carboxylate binding site. Such a result provides confidence that the dynamic ligation screening approach used here can encode and retain selectivity.

## Conclusions

The identification of inhibitors of β-sheet mediated PPI interfaces, which are generally shallow and elongated, is challenging. Here, we used dynamic ligation screening to identify peptide-fragment hybrids linked through an acylhydrazone bond, that are able to functionally mimic a β-strand and inhibit its PPI using GKAP/SHANK1 PDZ as a model interaction. The identified ligands were shown to bind with comparable potency to the GKAP PBM (Ac-EAQTRL-COOH) ligand from which they were derived and were selective when tested against an alternative PDZ domain. Crystallographic studies supported by molecular dynamics analyses indicated that the fragment portion of the hybrids was able to reinforce SHANK1 recognition by engaging in transient charge-reinforced and cation–π interactions on the protein surface. The observation of this distinct binding mode provides new insight on the molecular recognition of SHANK1 towards its peptide/protein ligands that can inform chemical probes development – a focus of our future studies.

More generally, our approach is advantageous in that it allows: (i) use of commercially available aldehyde fragments to build a small, diverse library then extend it to near neighbors based on initial hits; (ii) ligation to be performed under conditions where the reaction is reversible, and is compatible with the screening assay; (iii) validate hits easily without the need for a further synthetic step, thus providing useful insights on structure–activity relationships; and (iv) explore unoccupied binding sites on a protein surface in an unbiased manner to generate ligands with target affinity and selectivity. Significantly, whilst conventional fragment based approaches and reversible protein-directed fragment discovery in the form of disulfide-tethering have been used to develop modulators of PPIs,^78–80^ the use of ligand-directed fragment discovery to develop modulators of PPIs has not been widely developed. Thus, these proof-of concept results exemplified here point to future application of dynamic-ligation screening to identify and optimize PPI modulators more broadly.

## Author contributions

Z. H., T. A. E. and A. J. W. conceived and designed the research program, Z. H. designed studies and performed the research with support from F. H. and L.-Y. L. (NMR), S. C. (synthesis) and C. H. T. (crystallography). D. K. S. and R. B. S. performed molecular dynamics analyses. The manuscript was written by Z. H. and A. J. W. with contributions from all authors. Z. H. prepared all figures.

## Conflicts of interest

There are no conflicts to declare.

## Supplementary Material

SC-012-D0SC05694D-s001

SC-012-D0SC05694D-s002

SC-012-D0SC05694D-s003

SC-012-D0SC05694D-s004

SC-012-D0SC05694D-s005

SC-012-D0SC05694D-s006
