# Supplementary material for: Identification of β-strand mediated protein–protein interaction inhibitors using ligand-directed fragment ligation
Source: Chem Sci. 2021 Jan 6;12(6):2286–93. doi: 10.1039/d0sc05694d (PMC8179271; doi:10.1039/d0sc05694d)
Supplement: SC-012-D0SC05694D-s005 [file SC-012-D0SC05694D-s005.pdf]

Electronic Supplementary Material (ESI) for Chemical Science.
This journal is © The Royal Society of Chemistry 2020

Supporting information

Identification of β-strand mediated protein-protein interactions inhibitors using ligand-directed fragment ligation

Zsófia Hegedüs[1], Fruzsina Hóbor[2,3], Deborah K. Shoemark[5], Sergio Celis[1], Lu-Yun Lian[4], Chi H. Trinh[2,3], Richard B. Sessions[5], Thomas A. Edwards[2,3], Andrew J. Wilson[1,2]*

[1]School of Chemistry, University of Leeds, Woodhouse Lane, Leeds LS2 9JT, UK, [2]Astbury Centre for Structural Molecular Biology, University of Leeds, Woodhouse Lane, Leeds LS2 9JT, UK, [3]School of Molecular and Cellular Biology, University of Leeds, Woodhouse Lane, Leeds LS2 9JT, UK, [4]Institute of Integrative Biology, University of Liverpool, Liverpool L69 3BX, UK, [5]School of Biochemistry, Biomedical Sciences Building, University of Bristol, Bristol, BS8 1TD.

Table of contents

1. Supplementary tables and figures 4

Table S1. Aldehyde library used for screening 4

Scheme S1. Synthesis of hyrazones..................... 17

Scheme S2. Synthesis of hyrazones with hydrazide functionality on the N-terminus 17

Figure S1. Molecular properites of the aldehyde fragment library 18

Figure S2. Rate of hydrazone exchange 19

Figure S3. Fluorescence anisotropy direct titration of SHANK1 PDZ with FITC-Ahx-GKAP as tracer 19

Figure S4. Fluorescence anisotropy competition assay and fitted IC_{50} values for GKAP PDZ-binding motif (PBM) (Ac-EAQTRL-COOH), Ac-TRL-OH and compounds **1-, 2-, 3-, 4-A001**..................... 20

Figure S5. Results of the fluorescence anisotropy competition screening of hydrazones formed from a) **1**, b) **2**, c) **3**, d) **4** at 50 μM concentration.. 21

Figure S6. Results of the fluorescence anisotropy competition screening of hydrazones formed from a) **1**, b) **2**, c) **3**, d) **4** at 10 μM concentration.. 22

Figure S7. Fluorescence anisotropy competition assay and fitted IC_{50} values for GKAP PBM (Ac-EAQTRL-COOH) and the hybrid compounds selected for validation from the single point screens.. 23

Figure S8. Fluorescence anisotropy competition assay and fitted IC_{50} values for GKAP PBM (Ac-EAQTRL-COOH) and the hybrid compounds selected for validation from the single point screens. 24

Figure S9. Fluorescence anisotropy competition assay and fitted IC_{50} values for GKAP PBM (Ac-EAQTRL-COOH) and the hybrid compounds selected for validation from the single point screens. 24

Figure S10. Results of the fluorescence anisotropy competition screening at 50 μM for hydrazones formed from a) **1**, b) **2**, c) **3**, d) **4** and at 10 μM concentration for e) **1**, f) **2**, g) **3**, h) **4**. with aldehydes A130-165. 25

Figure S11. Fluorescence anisotropy competition assay and fitted IC_{50} values for GKAP PBM (Ac-EAQTRL-COOH) and the hybrid compounds selected for validation from the single point screens.. 26

Figure S12. Fluorescence anisotropy competition assay and fitted IC_{50} values for GKAP PBM (Ac-EAQTRL-COOH) and the hybrid compounds selected for validation from the single point screens.. 26

Figure S13. Results of the fluorescence anisotropy competition screening of the aldehyde fragments at 250 μM concentration.. 27

Figure S14. Raw ITC data and fitted thermograms for the tested hydrazone hits. . 28

Table S2. Calculated group efficiency values (GE) for GKAP and the hydrazides relative to the core TRL motif. 29

Table S3. Calculated group efficiency values (GE) for hydrazides relative to the core compound **3-A001**.. 30

Table S4. Calculated group efficiency values (GE) for hydrazides relative to the core compound **4-A001.** 31

Table S5. Statistics for obtained crystal structures. 32

Figure S15. Co-crystal structure of SHANK1 PDZ (green) with compound **3-A047** . 33

Figure S16. Co-crystal structure of SHANK1 PDZ (green) with compound **4-A047** .. 34

Figure S17. Co-crystal structure of SHANK1 PDZ (green) with compound **3-A048** .. 35

Figure S18. Co-crystal structure of SHANK1 PDZ (green) with compound **4-A048** .. 36

Figure S19. Overlays of co-crystal structures of GKAP in complex with SHANK1 PDZ with **3-A047**; **3-A048**; **4-A047** and **4-A048** 37

Table S6. RMSD values of aligned crystal structures with 1Q3P……………………………38

Figure S20. Fitted rotational correlation time of the SHANK1 residues with and without ligands 39

Figure S21. Molecular dynamics simulations for the the **3-A048**/SHANK1 interaction ……40

Figure S22. Overlay of HSQC plots for **4-A047** with Ligand:protein ratio ranging from 1:1 to 100:1………….. 41

Figure S23. Mapping of chemical shift changes onto the structure of PSD95 PDZ1. 41

Table S7. Concentration dependent shift changes of compound **4-A047** 42

Figure S24. Fitted EC_{50} value for compound **4-A047**. 42

Table S8. Relative affinities using magnitude of shift changes of residues whose chemical shifts are significantly affected 43

2. Materials and methods 44

2.1. Peptide Synthesis and purification 44

2.1.1. General methods 44

2.1.1. Synthesis of peptide hydrazides 45

2.2. Protein expression and purification 45

2.3. Aldehyde library selection 46

2.4. Fluorescence anisotropy 46

2.4.1. General comments 46

2.4.2. Direct titration 47

2.4.1. Competition assay 47

2.4.2. Library screening 48

2.5. Isothermal tritration calorimetry 49

2.6. Co-crystallization 49

2.7. Molecular dynamics simulations 50

2.8. NMR relaxation experiments 50

2.9. PSD95 PDZ1 Ligand Experiments 51

2.10. Compound characterization 52

2.11. References 69

1. Supplementary tables and figures

Table S1. Aldehyde library used for screening

#	NAME	Structure	Molweight	cLogP	H-Acc	H-Don	PSA
A001	Benzaldehyde	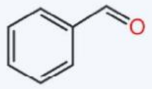	106.124	1.6	1	0	17.07
A002	Propionaldehyde	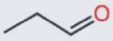	58.0796	0.29	1	0	17.07
A003	Butyraldehyde	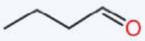	72.1065	0.75	1	0	17.07
A004	2-Methylpropionaldehyde	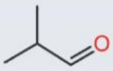	72.1065	0.51	1	0	17.07
A005	Isovaleraldehyde	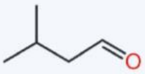	86.1334	1.0	1	0	17.07
A006	Trimethylacetaldehyde	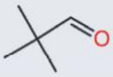	86.1334	1.1	1	0	17.07
A007	D-(+)-Glyceraldehyde	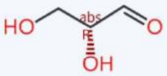	90.0776	-1.7	3	2	57.53
A008	Pyrrole-2-carboxaldehyde	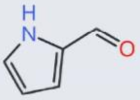	95.1007	0.37	2	1	32.86
A009	3-Furancarboxaldehyde	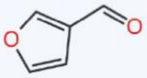	96.0848	0.73	2	0	30.21
A010	4-Imidazolecarboxaldehyde	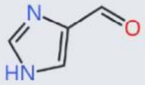	96.0888	-0.19	3	1	45.75
A011	3,3-Dimethylbutyraldehyde	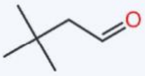	100.160	1.5	1	0	17.07
A012	methional	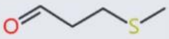	104.173	0.44	1	0	42.37
A013	2-Formylpyridine	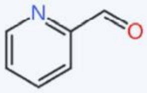	107.112	0.65	2	0	29.96

#	NAME	Structure	Molweight	cLogP	H-Acc	H-Don	PSA
A014	p-Formylpyridine	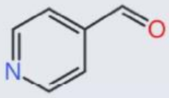	107.112	0.59	2	0	29.96
A015	1-Methyl-2-imidazolecarboxaldehyde	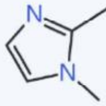	110.116	-0.22	3	0	34.89
A016	5-Methylisoxazole-3-carboxaldehyde	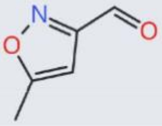	111.100	0.46	3	0	43.1
A017	5,6-Dihydro-2H-pyran-3-carbaldehyde	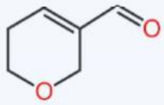	112.128	-0.086	2	0	26.3
A018	2-Thiophenecarboxaldehyde	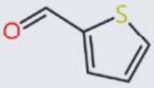	112.152	1.5	1	0	45.31
A019	Cyclohexanecarboxaldehyde	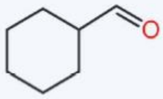	112.171	1.2	1	0	17.07
A020	Tetrahydro-2H-pyran-4-carbaldehyde	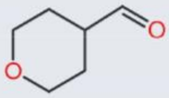	114.143	0.018	2	0	26.3
A021	3-(Methylthio)butanal	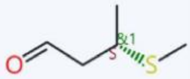	118.199	0.85	1	0	42.37
A022	o-Tolualdehyde	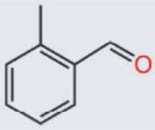	120.151	1.9	1	0	17.07
A023	Phenyl acetaldehyde	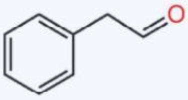	120.151	1.3	1	0	17.07
A024	4-Hydroxybenzaldehyde	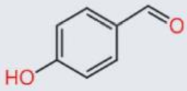	122.123	1.2	2	1	37.3
A025	Salicylaldehyde	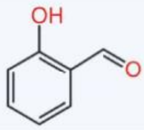	122.123	1.2	2	1	37.3
A026	4-Aminopyrimidine-5-carboxaldehyde	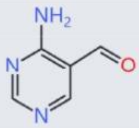	123.115	-0.30	4	1	68.87

#	NAME	Structure	Molweight	cLogP	H-Acc	H-Don	PSA
A027	45-Fluorobenzaldehyde	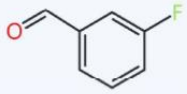	124.114	1.7	1	0	17.07
A028	4,5-Dimethyl-2-furaldehyde	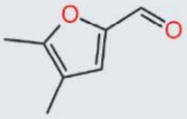	124.139	1.5	2	0	30.21
A029	4-Methylthiazole-5-carboxaldehyde	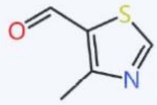	127.167	0.85	2	0	58.2
A030	2-Aminothiazole-5-carboxaldehyde	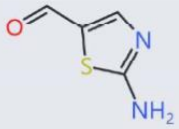	128.155	0.62	3	1	84.22
A031	4-Cyanobenzaldehyde	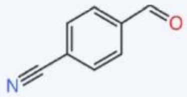	131.134	1.4	2	0	40.86
A032	4-Methoxybenzaldehyde	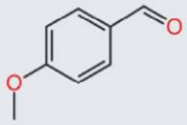	136.150	1.5	2	0	26.3
A033	6-Methoxy-2-pyridinecarboxaldehyde	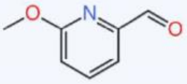	137.138	0.93	3	0	39.19
A034	4-Chlorobenzaldehyde	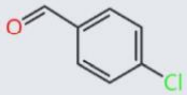	140.569	2.2	1	0	17.07
A035	4,5-Dimethylthiazole-2-carboxaldehyde	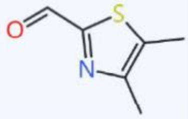	141.194	1.2	2	0	58.2
A036	2-Chloroisonicotinaldehyde	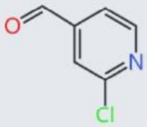	141.557	1.3	2	0	29.96
A037	4-chloropicolinaldehyde	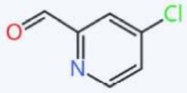	141.557	1.3	2	0	29.96
A038	Ethyl 2-formyl-1-cyclopropanecarboxylate, predominantly trans	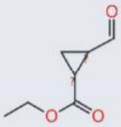	142.153	-0.016	3	0	43.37
A039	Indole-3-carboxaldehyde	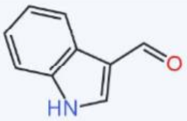	145.161	1.6	2	1	32.86

#	NAME	Structure	Molweight	cLogP	H-Acc	H-Don	PSA
A040	2-Benzofurancarboxaldehyde	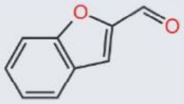	146.145	2.1	2	0	30.21
A041	7-Azaindole-3-carboxaldehyde	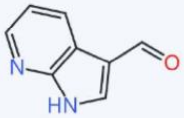	146.149	0.91	3	1	45.75
A042	1H-Indazole-3-carboxaldehyde	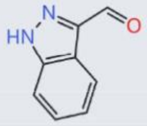	146.149	0.86	3	1	45.75
A043	(2Z)-2-Phenyl-2-butenal	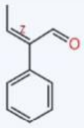	146.188	1.8	1	0	17.07
A044	1,2,4-Triazolo[1,5-a]pyridine-6-carboxaldehyde	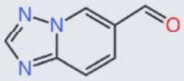	147.137	-0.066	4	0	47.26
A045	3-Phenylaziridine-2-carboxaldehyde dimer	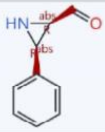	147.176	0.42	2	1	39.01
A046	Mesitaldehyde	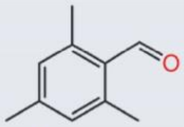	148.204	2.6	1	0	17.07
A047	2-Formylbenzoic acid	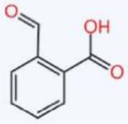	150.133	1.1	3	1	54.37
A048	4-Carboxybenzaldehyde	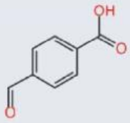	150.133	1.1	3	1	54.37
A049	Benzyloxyacetaldehyde	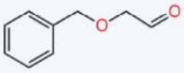	150.176	0.76	2	0	26.3
A050	(S)-(-)-Perillaldehyde	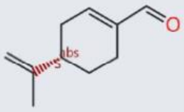	150.220	2.2	1	0	17.07
A051	Safranal	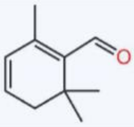	150.220	1.8	1	0	17.07
A052	2,4-Dihydroxy-6-methylbenzaldehyde	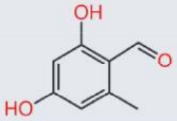	152.149	1.2	3	2	57.53

#	NAME	Structure	Molweight	cLogP	H-Acc	H-Don	PSA
A053	3-(5-Methyl-2-furyl)butanal	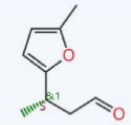	152.192	1.7	2	0	30.21
A054	4-(Methylthio)benzaldehyde	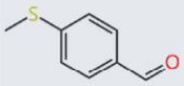	152.217	2.1	1	0	42.37
A055	Citral	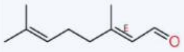	152.236	3.3	1	0	17.07
A056	5-(Ethoxymethyl)furan-2-carboxaldehyde	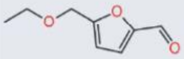	154.164	1.1	3	0	39.44
A057	(R)-(+)-Citronellal	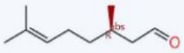	154.252	3.1	1	0	17.07
A058	3-Formyl-2-thienylboronic acid	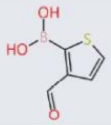	155.969	1.1	3	2	85.77
A059	1-Naphthaldehyde	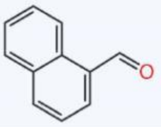	156.184	2.8	1	0	17.07
A060	2-Naphthaldehyde	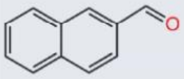	156.184	2.8	1	0	17.07
A061	Quinaldaldehyde	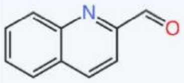	157.172	2.0	2	0	29.96
A062	N-Boc-2-aminoacetaldehyde	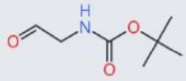	159.184	0.27	4	1	55.4
A063	1-Methyl-1H-indole-6-carboxaldehyde	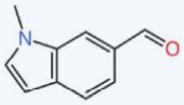	159.187	1.8	2	0	22
A064	2-Chloro-3-fluoropyridine-4-carboxaldehyde	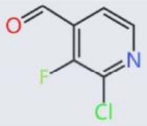	159.547	1.4	2	0	29.96
A065	1-methylpyrrolo[2,3-b]pyridine-3-carbaldehyde	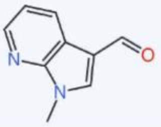	160.176	1.0	3	0	34.89

#	NAME	Structure	Molweight	cLogP	H-Acc	H-Don	PSA
A066	Thianaphthene-3-carboxaldehyde	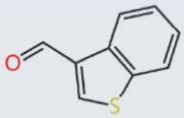	162.212	2.4	1	0	45.31
A067	Benzothiazole-2-carboxaldehyde	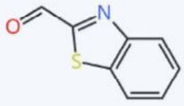	163.200	1.6	2	0	58.2
A068	3-Formyl-5-methylphenylboronic acid	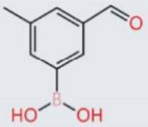	163.967	1.7	3	2	57.53
A069	2,5-Dimethoxybenzaldehyde	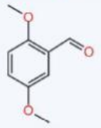	166.175	1.5	3	0	35.53
A070	2,4-Dimethoxybenzaldehyde	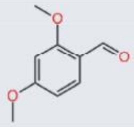	166.175	1.5	3	0	35.53
A071	Ethyl 4-formylpyrrole-2-carboxylate	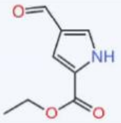	167.163	0.69	4	1	59.16
A072	(5-Methyl-2,4-dioxo-3,4-dihydro-1(2H)-pyrimidinyl)acetaldehyde	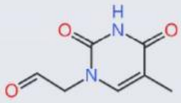	168.152	-1.1	5	1	66.48
A073	Thieno[3,2-b]thiophene-2-carboxaldehyde	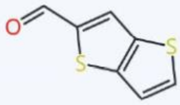	168.240	2.2	1	0	73.55
A074	(R)-1,4-Dioxaspiro[4.5]decane-2-carboxaldehyde	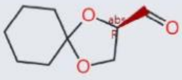	170.207	0.39	3	0	35.53
A075	Hydroxycitronellal	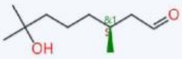	172.267	2.1	2	1	37.3
A076	4-(Trifluoromethyl)benzaldehyde	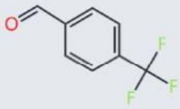	174.121	2.4	1	0	17.07
A077	5-Methoxyindole-3-carboxaldehyde	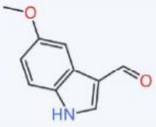	175.186	1.6	3	1	42.09
A078	4-(tert-Butyloxy)benzaldehyde	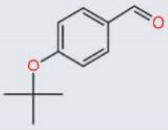	178.230	2.7	2	0	26.3

#	NAME	Structure	Molweight	cLogP	H-Acc	H-Don	PSA
A079	5-Chloroindole-3-carboxaldehyde	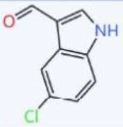	179.606	2.2	2	1	32.86
A080	1-(2-Tetrahydropyranyl)-1H-pyrazole-5-carboxaldehyde	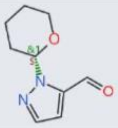	180.206	0.83	4	0	44.12
A081	2,4-Dichlorothiazole-5-carboxaldehyde	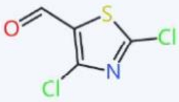	182.030	2.3	2	0	58.2
A082	4-Biphenylcarbaldehyde	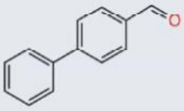	182.221	3.3	1	0	17.07
A083	4-(2-Pyridinyl)benzaldehyde	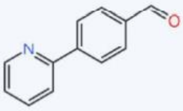	183.209	2.3	2	0	29.96
A084	1-(4-Methylphenyl)pyrrole-2-carbaldehyde	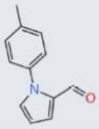	185.225	2.2	2	0	22
A085	2-Chloro-6-nitrobenzaldehyde	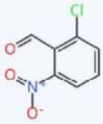	185.566	1.3	4	0	62.89
A086	3,5-Difluoro-4-formylphenylboronic acid	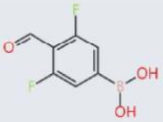	185.921	1.5	3	2	57.53
A087	6-Bromo-3-pyridinecarboxaldehyde	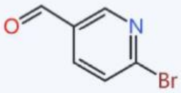	186.008	1.4	2	0	29.96
A088	5-Bromonicotinaldehyde	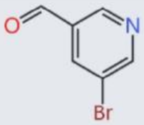	186.008	1.3	2	0	29.96
A089	2-Bromoisonicotinaldehyde	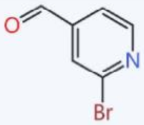	186.008	1.4	2	0	29.96
A090	3-Methyl-1-phenyl-1H-pyrazole-4-carboxaldehyde	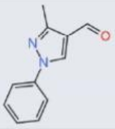	186.213	1.2	3	0	34.89
A091	2-Butyl-5-chloro-1H-imidazole-4-carboxaldehyde	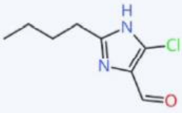	186.641	2.1	3	1	45.75

#	NAME	Structure	Molweight	cLogP	H-Acc	H-Don	PSA
A092	4-Phenylthiophene-2-carboxaldehyde	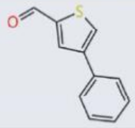	188.250	3.1	1	0	45.31
A093	6-(3-Thienyl)pyridine-2-carboxaldehyde	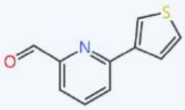	189.238	2.2	2	0	58.2
A094	4-(1-Piperidinyl)benzaldehyde	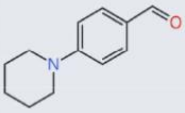	189.257	2.4	2	0	20.31
A095	5-Bromothiophene-3-carboxaldehyde	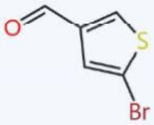	191.048	2.3	1	0	45.31
A096	4-Bromothiazole-2-carboxaldehyde	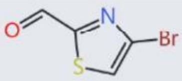	192.036	1.2	2	0	58.2
A097	2-Bromothiazole-5-carboxaldehyde	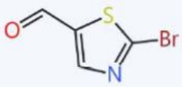	192.036	1.7	2	0	58.2
A098	2-Bromothiazole-4-carboxaldehyde	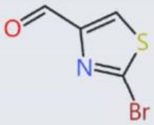	192.036	1.7	2	0	58.2
A099	4-Diethylaminosalicylaldehyde	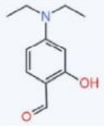	193.245	2.0	3	1	40.54
A100	6-Nitropiperonal	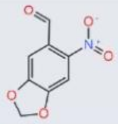	195.130	0.78	6	0	81.35
A101	Trimethoxybenzaldehyde	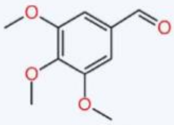	196.201	1.4	4	0	44.76
A102	4-Phenoxybenzaldehyde	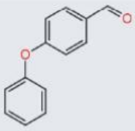	198.220	3.0	2	0	26.3
A103	Boc-L-prolinal	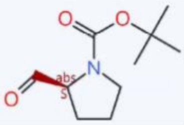	199.249	1.2	4	0	46.61
A104	1-Allyl-2-methyl-1H-indole-3-carbaldehyde	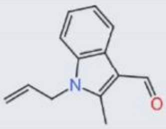	199.252	2.7	2	0	22

#	NAME	Structure	Molweight	cLogP	H-Acc	H-Don	PSA
A105	2-Bromo-5-hydroxybenzaldehyde	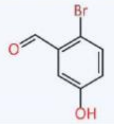	201.019	2.0	2	1	37.3
A106	4-Bromo-2-fluorobenzaldehyde	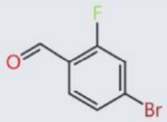	203.010	2.4	1	0	17.07
A107	N-Benzylpiperidine-4-carboxaldehyde	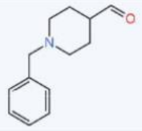	203.284	1.5	2	0	20.31
A108	2-Chloro-6-methylquinoline-3-carboxaldehyde	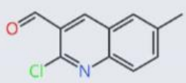	205.644	3.0	2	0	29.96
A109	N-(3-Formyl-2-pyridinyl)-2,2-dimethylpropanamide	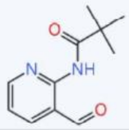	206.244	1.9	4	1	59.06
A110	Methyl 3-formyl-4-nitrobenzoate	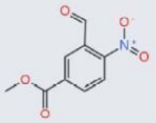	209.157	0.58	6	0	89.19
A111	3-Formyl-6-isopropylchromone	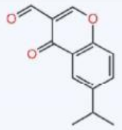	216.235	1.9	3	0	43.37
A112	2,3-Dimethoxy-1-naphthaldehyde	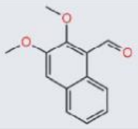	216.235	2.6	3	0	35.53
A113	2-Methyl-N-ethyl-N-(2-cyanoethyl)-4-aminobenzaldehyde	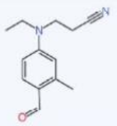	216.283	2.3	3	0	44.1
A114	2,3,6,7-Tetrahydro-8-hydroxy-1H,5H-benzo[ij]quinolizine-9-carboxaldehyde	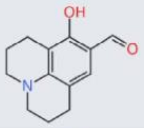	217.267	2.2	3	1	40.54
A115	4,6-Dichloro-2-(methylthio)pyrimidine-5-carboxaldehyde	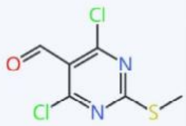	223.083	2.2	3	0	68.15
A116	5-Bromoindole-3-carboxaldehyde	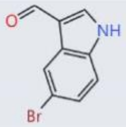	224.057	2.4	2	1	32.86
A117	6-Bromo-1H-indazole-4-carboxaldehyde	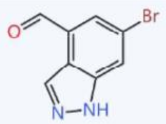	225.045	1.5	3	1	45.75

#	NAME	Structure	Molweight	cLogP	H-Acc	H-Don	PSA
A118	3-Bromo-5-nitrobenzaldehyde	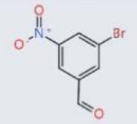	230.017	1.4	4	0	62.89
A119	1-(Phenylsulfonyl)-2-pyrrolecarboxaldehyde	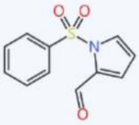	235.262	0.62	4	0	64.52
A120	3-Bromobenzothiophene-2-carboxaldehyde	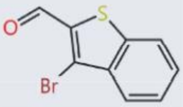	241.108	3.2	1	0	45.31
A121	3,5-Di-tert-butyl-2-methoxybenzaldehyde	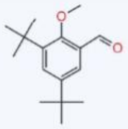	248.365	4.7	2	0	26.3
A122	3,5-Dibromopyridine-2-carboxaldehyde	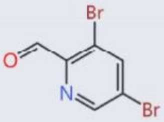	264.904	2.1	2	0	29.96
A123	3,4-Dibromothiophene-2-carboxaldehyde	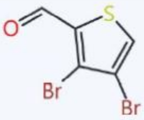	269.944	2.9	1	0	45.31
A124	5'-Bromo-2,2'-bithiophene-5-carboxaldehyde	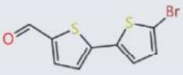	273.174	4.3	1	0	73.55
A125	5-[2-Chloro-5-(trifluoromethyl)phenyl]furfural	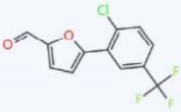	274.625	4.0	2	0	30.21
A126	3-Hydroxy-2-iodo-4-methoxybenzaldehyde	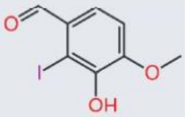	278.041	1.6	3	1	46.53
A127	5-Chloro-2-hydroxy-3-iodobenzaldehyde	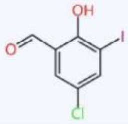	282.460	2.3	2	1	37.3
A128	1-Boc-4-(2-formylphenyl)piperazine	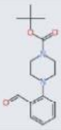	290.362	2.8	5	0	49.85
A129	5-(4-Bromophenyl)-2-chloropyridine-3-carboxaldehyde	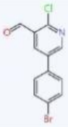	296.551	3.7	2	0	29.96
A130	5-Formyl-1H-pyrrole-2-carboxylic acid	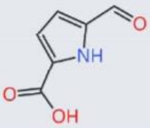	139.110	-0.091	4	2	70.16

#	NAME	Structure	Molweight	cLogP	H-Acc	H-Don	PSA
A131	5-Formyl-2-furoic acid	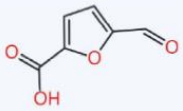	140.094	0.32	4	1	67.51
A132	4-Formyl-1H-pyrazole-3-carboxylic acid	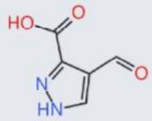	140.098	-1.0	5	2	83.05
A133	3-Formylbenzoic acid	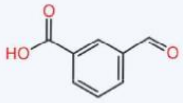	150.133	1.1	3	1	54.37
A134	2-(4-Formyl-1H-imidazol-1-yl)acetic acid	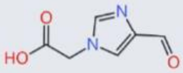	154.125	-1.3	5	1	72.19
A135	5-Formyl-2-thiophenecarboxylic acid	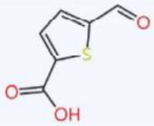	156.161	1.0	3	1	82.61
A136	5-Formylsalicylic acid	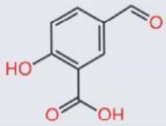	166.132	0.73	4	2	74.6
A137	5-Formyl-2,4-dimethyl-1H-pyrrole-3-carboxylic acid	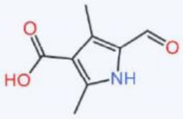	167.163	0.60	4	2	70.16
A138	2-fluoro-5-formylbenzoic acid	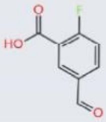	168.123	1.2	3	1	54.37
A139	4-fluoro-3-formyl-benzoic acid	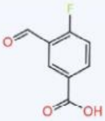	168.123	1.2	3	1	54.37
A140	4-Formylcinnamic acid, predominantly trans	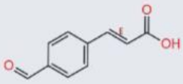	176.171	1.4	3	1	54.37
A141	2-formylcinnamic acid	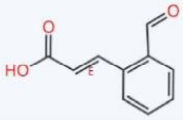	176.171	1.4	3	1	54.37
A142	5-formyl-2-methoxybenzoic acid	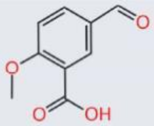	180.159	1.0	4	1	63.6
A143	4-Formyl-1,2,5-trimethyl-1H-pyrrole-3-carboxylic acid	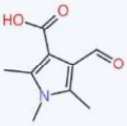	181.190	0.73	4	1	59.3

#	NAME	Structure	Molweight	cLogP	H-Acc	H-Don	PSA
A144	5-chloro-2-formyl-benzoic acid	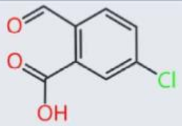	184.578	1.7	3	1	54.37
A145	3-formyl-1H-indole-7-carboxylic acid	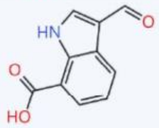	189.170	1.1	4	2	70.16
A146	4-Formylquinoline-8-carboxylic acid	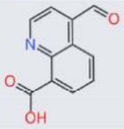	201.181	1.4	4	1	67.26
A147	4-(2-Formyl-1H-pyrrol-1-yl)benzoic acid	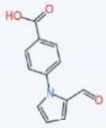	215.207	1.3	4	1	59.3
A148	3-(3-Formyl-1H-indol-1-yl)propanoic acid	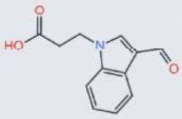	217.223	1.2	4	1	59.3
A149	2-(3-formyl-1H-indol-1-yl)propanoic acid	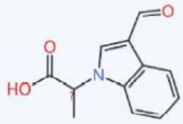	217.223	1.2	4	1	59.3
A150	6-Formyl-2,4-dimethyl-4H-thieno[3,2-b]pyrrole-5-carboxylic acid	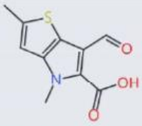	223.251	1.4	4	1	87.54
A151	3'-Formyl-biphenyl-3-carboxylic acid	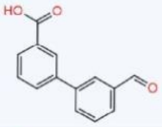	226.230	2.7	3	1	54.37
A152	2?-Formyl[1,1?-biphenyl]-2-carboxylic acid	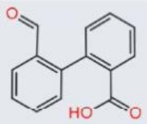	226.230	2.7	3	1	54.37
A153	4'-formyl-biphenyl-4-carboxylic acid	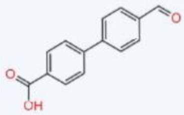	226.230	2.7	3	1	54.37
A154	2-(4-formylphenyl)isonicotinic acid	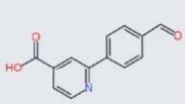	227.218	1.8	4	1	67.26
A155	4-(5-Formyl-2-furyl)-2-hydroxybenzoic acid	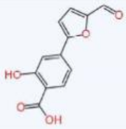	232.191	1.7	5	2	87.74
A156	4-(3-methoxy-4-formyl)phenoxybutyric acid	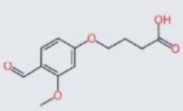	238.238	1.5	5	1	72.83

#	NAME	Structure	Molweight	cLogP	H-Acc	H-Don	PSA
A157	4-(3-formyl-2,5-dimethyl-1H-pyrrol-1-yl)benzenecarboxylic acid	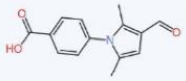	243.261	2.1	4	1	59.3
A158	3-Formyl-1-isobutyl-1H-indole-4-carboxylic acid	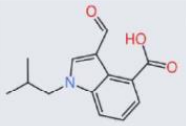	245.277	2.2	4	1	59.3
A159	4-chloro-3-(5-formyl-furan-2-yl)-benzoic acid	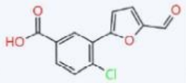	250.637	2.6	4	1	67.51
A160	3-(3-formylphenoxymethyl)benzoic acid	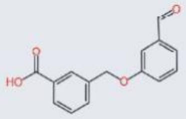	256.256	2.4	4	1	63.6
A161	2-(3-formylphenoxy)-2-phenylacetic acid	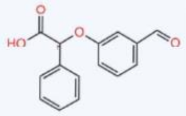	256.256	2.0	4	1	63.6
A162	3-(3-Formyl-2,5-dimethyl-pyrrol-1-yl)-2-methyl-benzoic acid	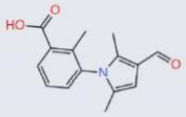	257.288	2.4	4	1	59.3
A163	(2-Chloro-6-ethoxy-4-formylphenoxy)acetic acid	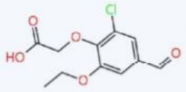	258.656	1.6	5	1	72.83
A164	5-(4-formylphenoxymethyl)-3-methylfuran-2-carboxylic acid	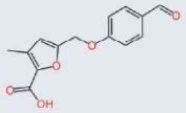	260.244	2.0	5	1	76.74
A165	5-((3-Formyl-1H-indol-1-yl)methyl)furan-2-carboxylic acid	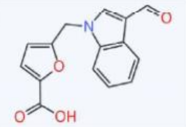	269.255	1.9	5	1	72.44

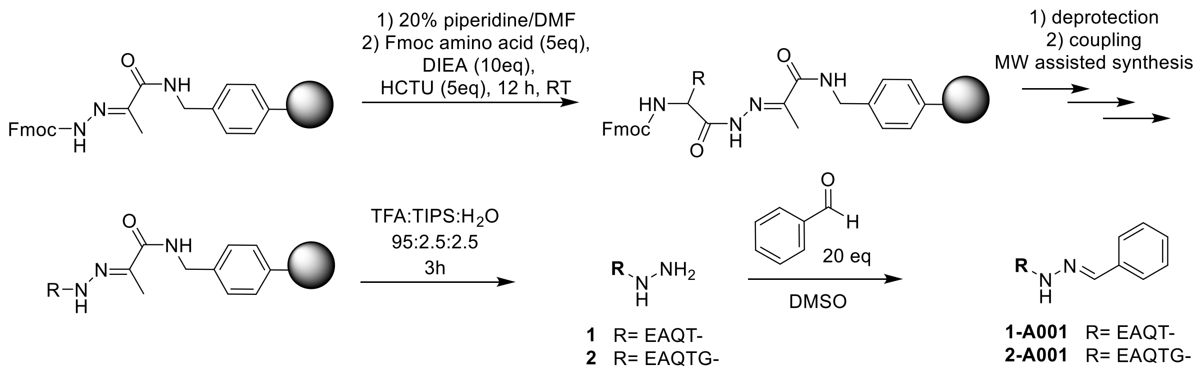

Scheme S1. Synthesis of hyrazones with hydrazide functionality on the C-terminus (compounds **1** and **2** and the corresponding hydrazone **1-A001** and **2-A001**) using Fmoc-NHN=Pyv Resin (Iris Biotech).

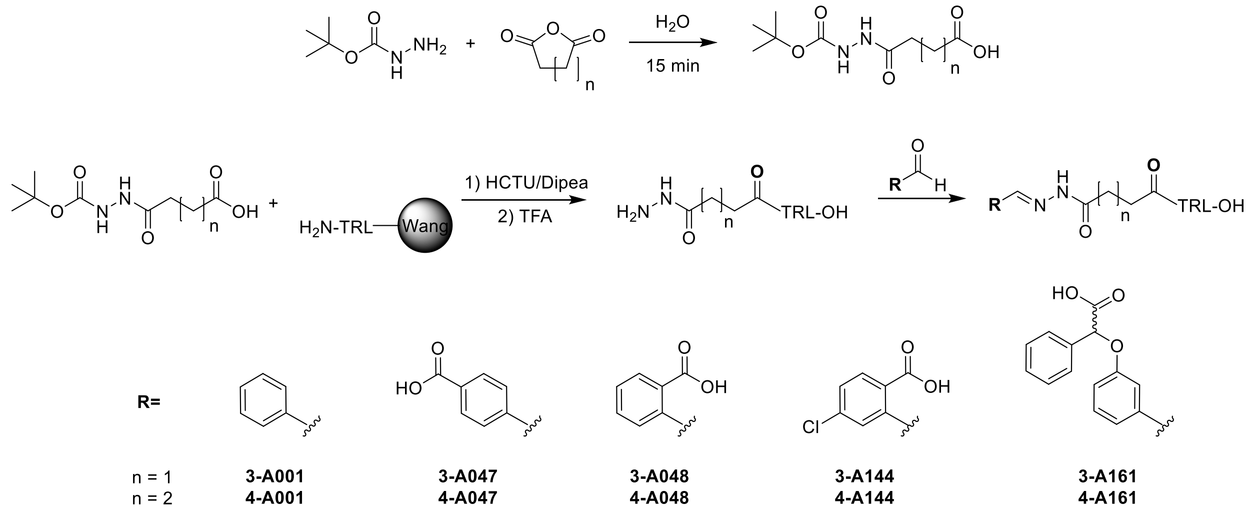

Scheme S2. Synthesis of hyrazones with hydrazide functionality on the N-terminus. The absolute configuration and enantiomeric purity of fragment A161 is unknown.

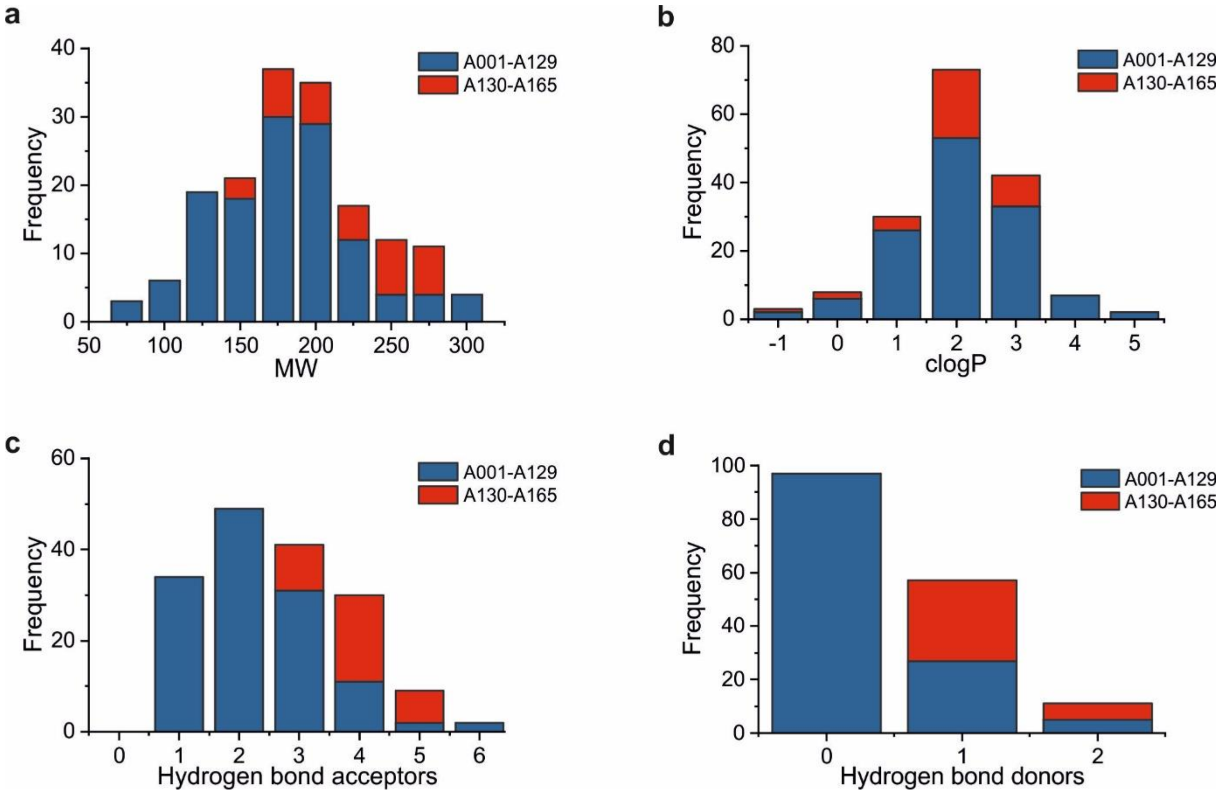

Figure S1. Molecular properites of the aldehyde fragment library: a) molecular weight distribution, b) clogP disctribution c) number of hydrogen bond acceptors and d) number of hydrogen bond donors. Blue bars correspond to the diverse aldehyde set (A001-A129), red bars correspond to a subset of compounds having additional carboxylic acid functionality (A130-165). Diversity was assessed using 2D molecular descriptors (FragFp, DataWarrior[1]), which resulted in 119 clusters with 0.85 Tanimoto similarity.

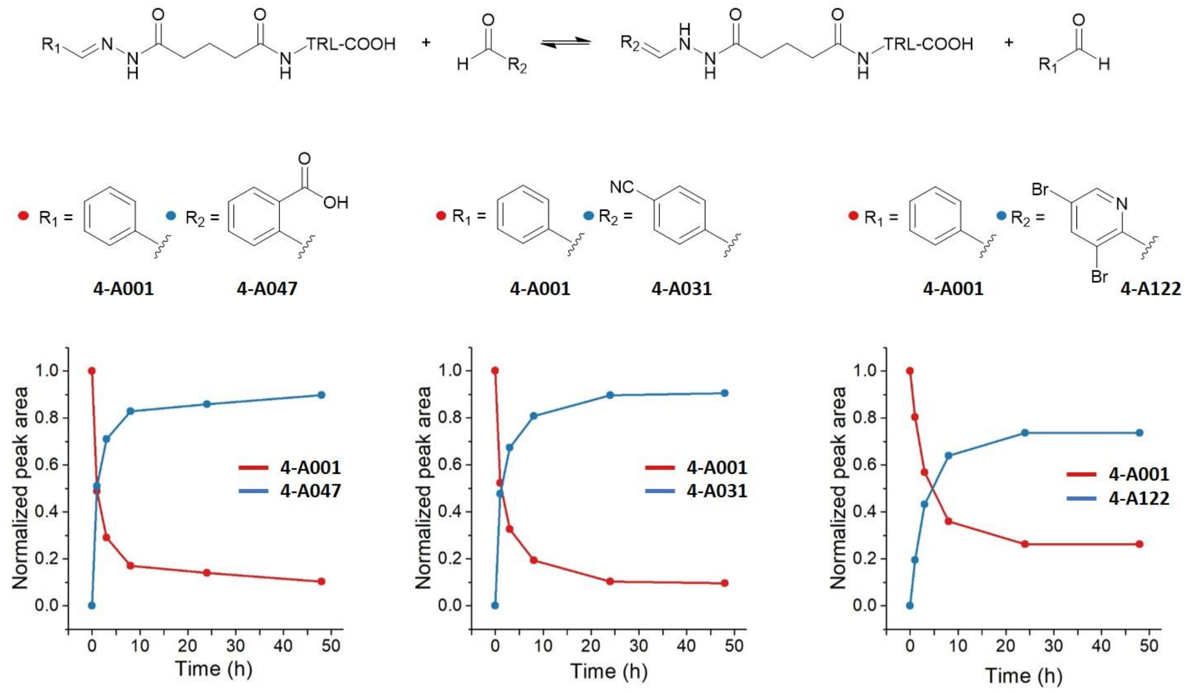

Figure S2. Rate of hydrazone exchange in 50 mM ammonium-acetate (NH_4Ac), pH 6.5, 10 mM aniline buffer using 100 µM compound **4-A001** and 5 equivalents of three different aldehydes (corresponding to R_2 groups) at room temperature. Hydrazone exchange was monitored by LC-MS; peaks for extracted ion chromatograms were integrated and normalized. Red lines represent the decreasing concentration of compound **4-A001**, blue lines represent the formation of the new hydrazone.

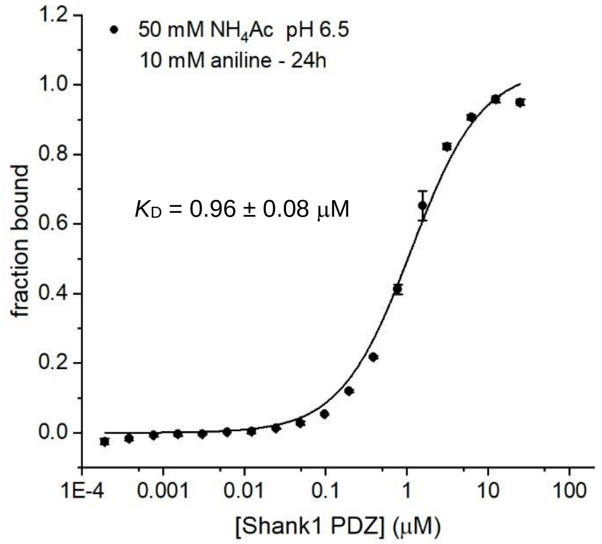

Figure S3. Fluorescence anisotropy direct titration of SHANK1 PDZ in 50 mM NH_4Ac, pH 6.5 10 mM aniline containing buffer using 50 nM FITC-Ahx-GKAP as tracer. Plate was read after 24 hr incubation at room temperature.

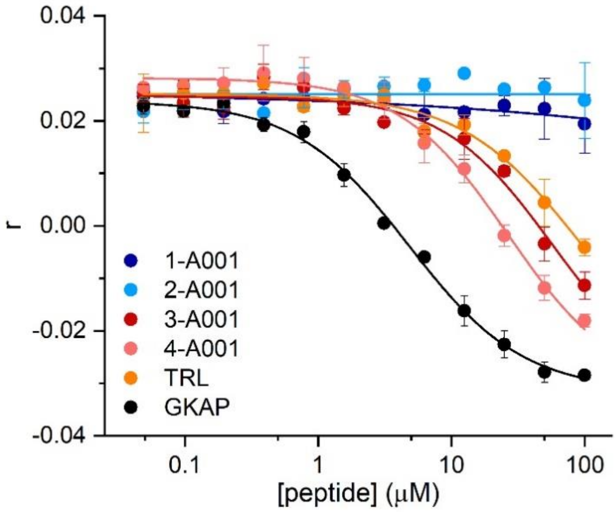

Competitor	IC_{50} (μM)
**GKAP**	4.5 ± 0.4
**AC-TRL-OH**	68 ± 5
**1-A001**	nd
**2-A001**	nd
**3-A001**	51 ± 3
**4-A001**	19 ± 3

Figure S4. Fluorescence anisotropy competition assay and fitted IC_{50} values for GKAP PDZ-binding motif (PBM) (Ac-EAQTRL-COOH), Ac-TRL-OH and compounds **1-, 2-, 3-, 4-A001** in 50 mM NH_4Ac, pH 6.5 10 mM aniline containing buffer using 1 μM Shank1 PDZ and 10 nM FITC-Ahx-TRL-COOH tracer; plates were read immediately at room temperature.

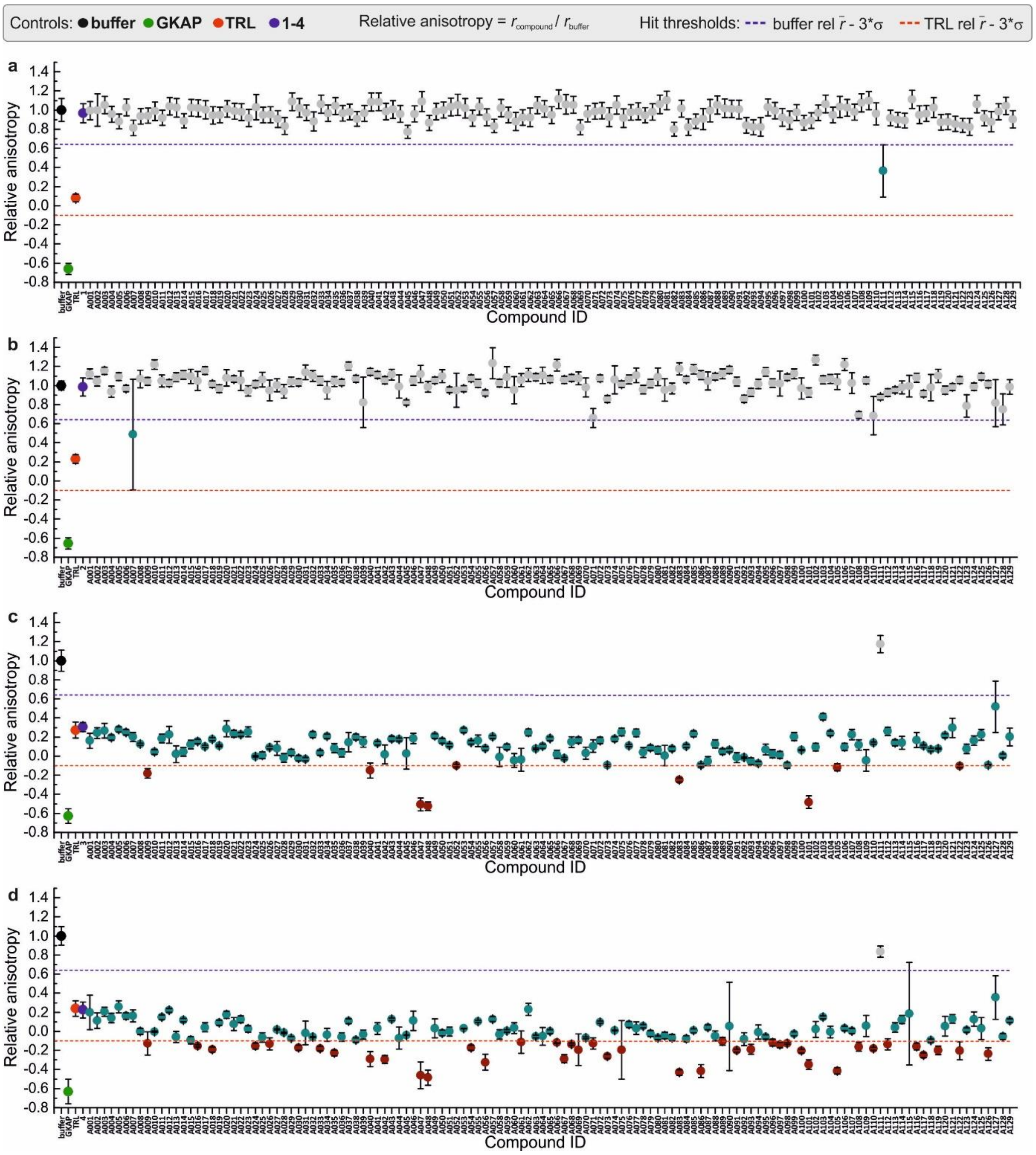

Figure S5. Results of the fluorescence anisotropy competition screening of hydrazones formed from a) **1**, b) **2**, c) **3**, d) **4** at 50 μM concentration. Compounds incubated with 5 equivalents of aldehydes (A001-A129) individually, for 24 hours before tracer addition. Assay was performed in 50 mM NH_4Ac buffer, pH 6.5, 10 mM aniline using 1 μM final protein concentration and 10 nM FITC-Ahx-TRL-COOH as tracer at room temperature. All assays included a buffer (black), GKAP PBM (Ac-EAQTRL-COOH) (green) and TRL (red) control. Anisotropy is expressed relative to the buffer control, where no competitor is present. Each dot on the graph represents the relative activity of a hybrid compound. Hit thresholds were defined as i) buffer r_{rel}-3σ (blue dashed line) and ii) TRL r_{rel}-3σ (red dashed line). Hybrids reaching these thresholds are colored cyan and red respectively. Error bars represent the standard deviation of sample duplicates.

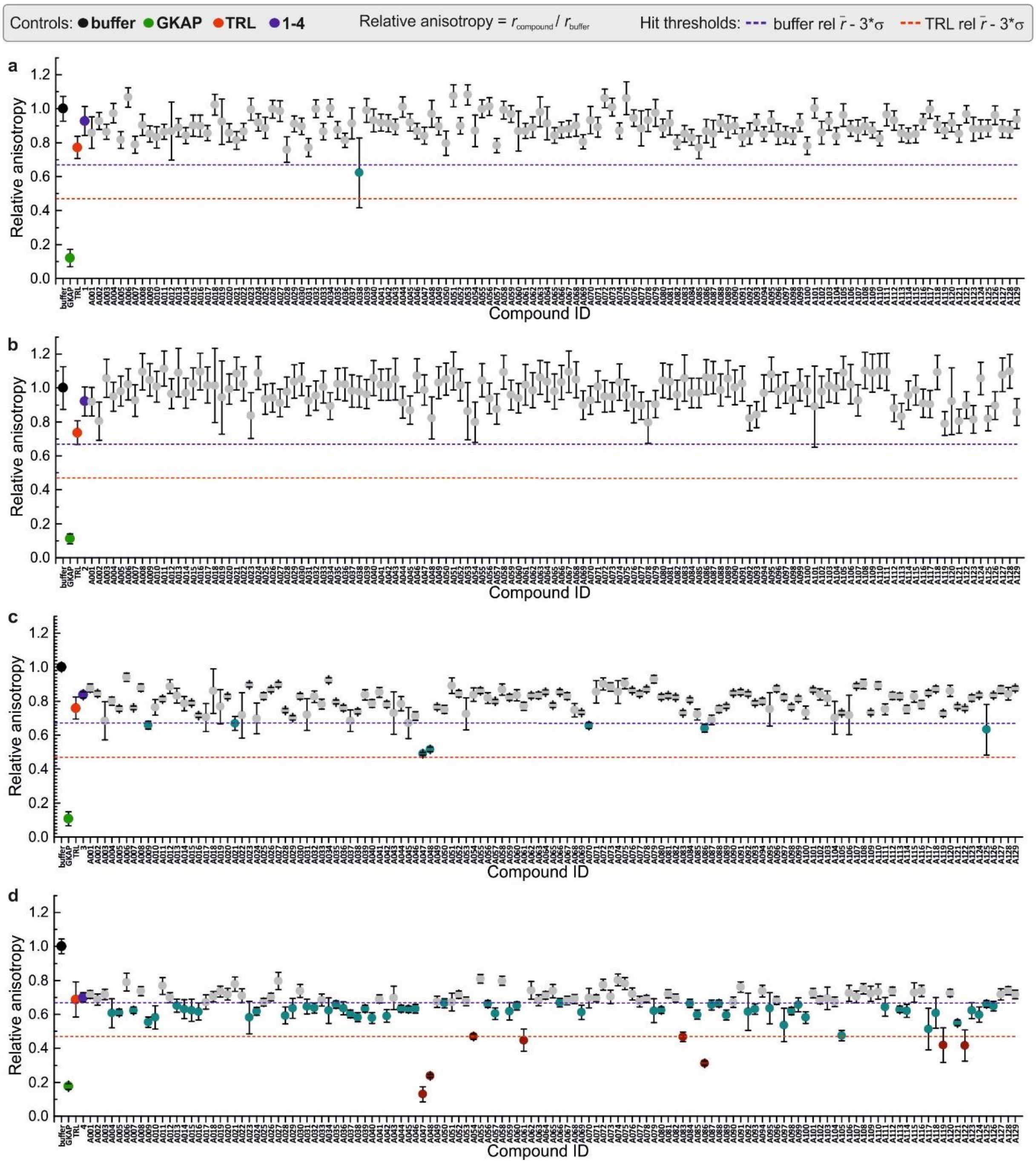

Figure S6. Results of the fluorescence anisotropy competition screening of hydrazones formed from a) **1**, b) **2**, c) **3**, d) **4** at 10 μM concentration. Compounds incubated with 5 equivalents of aldehydes (A001-A129) individually, for 24 hours before tracer addition. Assay was performed in 50 mM NH_4Ac buffer, pH 6.5, 10 mM aniline using 1 μM final protein concentration and 10 nM FITC-Ahx-TRL-COOH as tracer at room temperature. All assays included a buffer (black), GKAP PBM (Ac-EAQTRL-COOH) (green) and TRL (red) control. Anisotropy is expressed relative to the buffer control, where no competitor is present. Each dot on the graph represents the relative activity of a hybrid compound. Hit thresholds were defined as i) buffer r_{rel}-3σ (blue dashed line) and ii) TRL r_{rel}-3σ (red dashed line). Hybrids reaching these thresholds are colored cyan and red respectively. Error bars represent the standard deviation of sample duplicates.

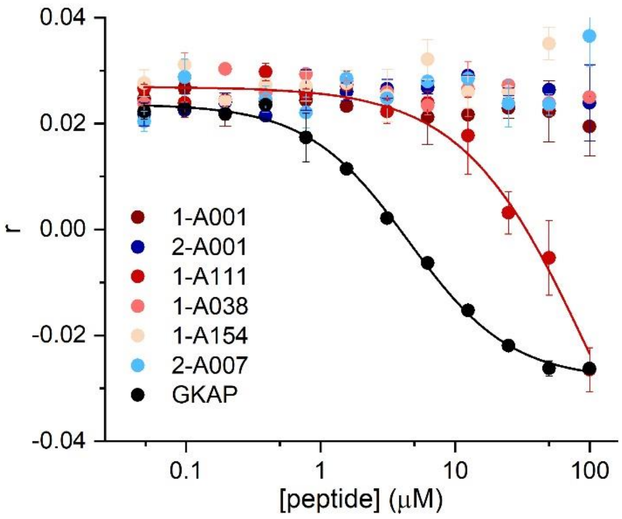

Competitor	IC_{50} (μM)
**GKAP**	4.5 ± 0.4
**1-A001**	nd
**2-A001**	nd
**1-A111**	97 ± 143
**1-A038**	nd
**1-A154**	nd
**2-A007**	nd

Figure S7. Fluorescence anisotropy competition assay and fitted IC_{50} values for GKAP PBM (Ac-EAQTRL-COOH) and the hybrid compounds selected for validation from the single point screens. Compounds **1-A001** and **2-A001** were incubated with 5 equivalents of aldehydes in 50 mM NH_4Ac, pH 6.5 10 mM aniline containing buffer at room termparture in presence of protein for 24 hours, after which 10 nM FITC-Ahx-TRL-COOH was added as tracer. Final SHANK1 PDZ concentration was 1 μM. Error bars represent standard deviation of duplicate samples. Compund **1-A111** resulted in protein precipitation and alteration of fluorescence intensity, therefore this compound was excluded from further screens.

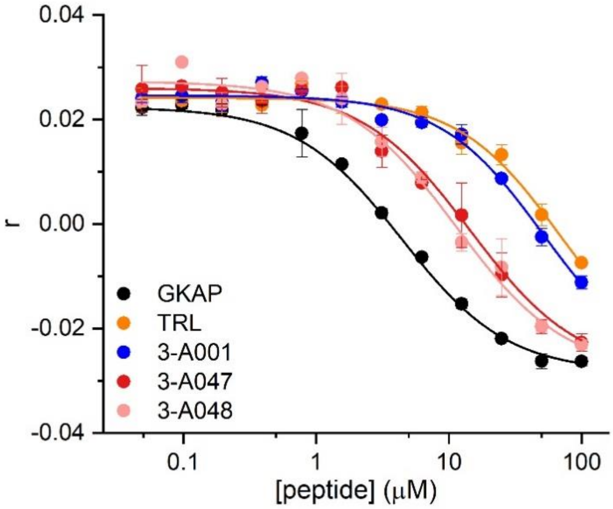

Competitor	IC_{50} (μM)
GKAP	4.5 ± 0.4
AC-TRL-OH	68 ± 5
3-A001	51 ± 3
3-A047	14 ± 1
3-A048	11 ± 1

Figure S8. Fluorescence anisotropy competition assay and fitted IC_{50} values for GKAP PBM (Ac-EAQTRL-COOH) and the hybrid compounds selected for validation from the single point screens. Compound **3-A001** was incubated with 5 equivalents of aldehydes in 50 mM NH_4Ac, pH 6.5 10 mM aniline containing buffer at room termparture in the presence of protein for 24 hours, after which 10 nM FITC-Ahx-TRL-COOH was added as tracer. The final SHANK1 PDZ concentration was 1 μM. Error bars represent standard deviation of duplicate samples.

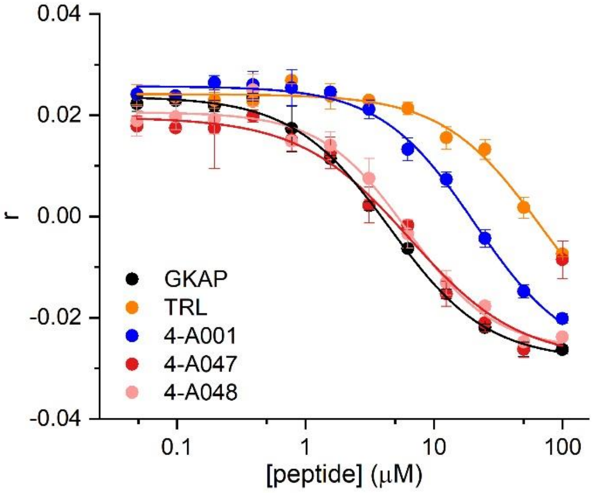

Competitor	IC_{50} (μM)
GKAP	4.5 ± 0.4
AC-TRL-OH	68 ± 5
4-A001	19 ± 3
4-A047	7.3 ± 0.6
4-A048	7.5 ± 0.8

Figure S9. Fluorescence anisotropy competition assay and fitted IC_{50} values for GKAP PBM (Ac-EAQTRL-COOH) and the hybrid compounds selected for validation from the single point screens. Compound **4-A001** was incubated with 5 equivalents of aldehydes in 50 mM NH_4Ac, pH 6.5 10 mM aniline containing buffer at room termparture in the presence of protein for 24 hours, after which 10 nM FITC-Ahx-TRL-COOH was added as tracer. The final SHANK1 PDZ concentration was 1 μM. Error bars represent standard deviation of duplicate samples.

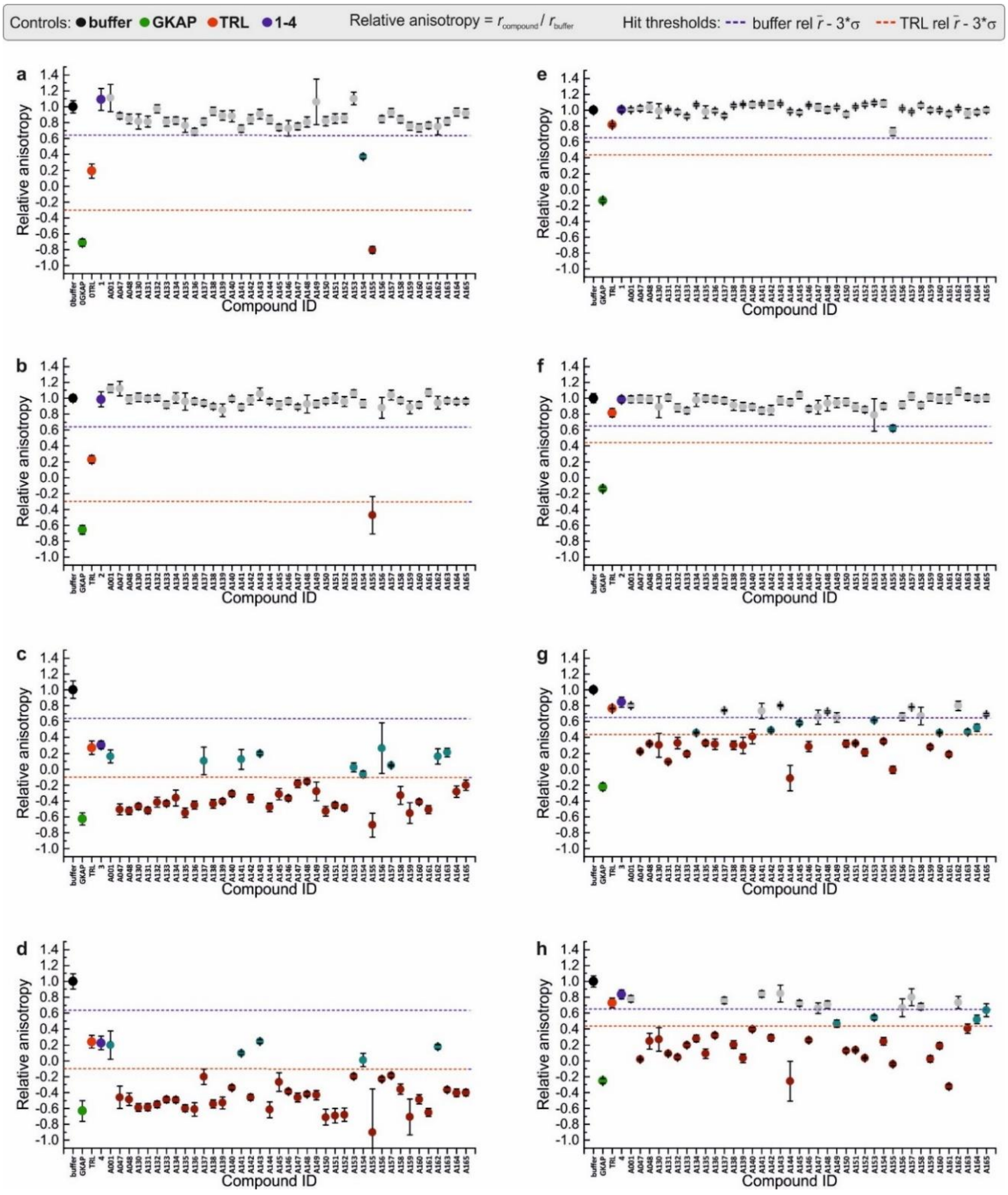

Figure S10. Results of the fluorescence anisotropy competition screening at 50 μM for hydrazones formed from a) **1**, b) **2**, c) **3**, d) **4** and at 10 μM concentration for e) **1**, f) **2**, g) **3**, h) **4**. Compounds were individually incubated with 5 equivalents of aldehydes (A130-165) for 24 hours before tracer addition. The assay was performed in 50 mM NH_4Ac buffer, pH 6.5, 10 mM aniline using 1 μM final protein concentration and 10 nM FITC-Ahx-TRL-COOH as tracer at room temperature. All assays included a buffer (black), GKAP PBM (Ac-EAQTRL-COOH) (green) and TRL (red) control. Anisotropy is expressed relative to the buffer control, where no competitor is present. Each dot on the graph represents the relative activity of a hybrid compound formed with different aldehyde fragments. Hit thresholds were defined as i) buffer r_{rel}-3σ (blue dashed line) and ii) TRL r_{rel}-3σ (red dashed line). Hybrids reaching these thresholds are colored cyan and red respectively. Error bars represent the standard deviation of sample duplicates.

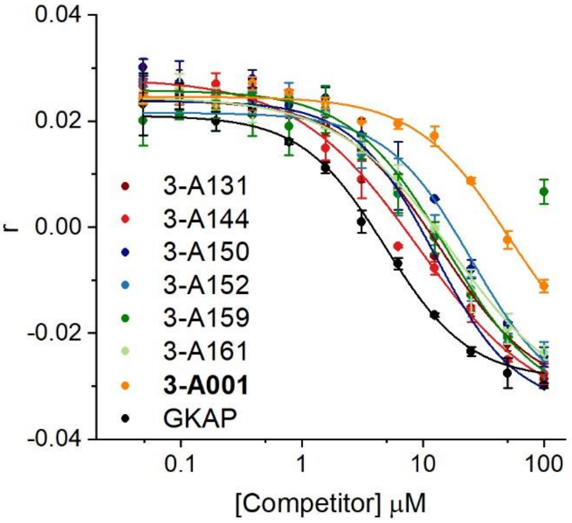

Competitor	IC_{50} (μM)
**GKAP**	4.5 ± 0.4
**3-A001**	51 ± 3
**3-A131**	12.5 ± 0.4
**3-A144**	**9.0 ± 1.6**
**3-A150**	25.3 ± 0.9
**3-A152**	15.7 ± 0.8
**3-A159**	13.9± 1.1
**3-A161**	17.6 ± 0.3

Figure S11. Fluorescence anisotropy competition assay and fitted IC_{50} values for GKAP PBM (Ac-EAQTRL-COOH) and the hybrid compounds selected for validation from the single point screens. Compounds **3-A001** was incubated with 5 equivalents of aldehydes in 50 mM NH_4Ac, pH 6.5 10 mM aniline containing buffer at room termparture in the presence of protein for 24 hours, after which 10 nM FITC-Ahx-TRL-COOH was added as tracer. The final SHANK1 PDZ concentration was 1 μM. Error bars represent standard deviation of duplicate samples.

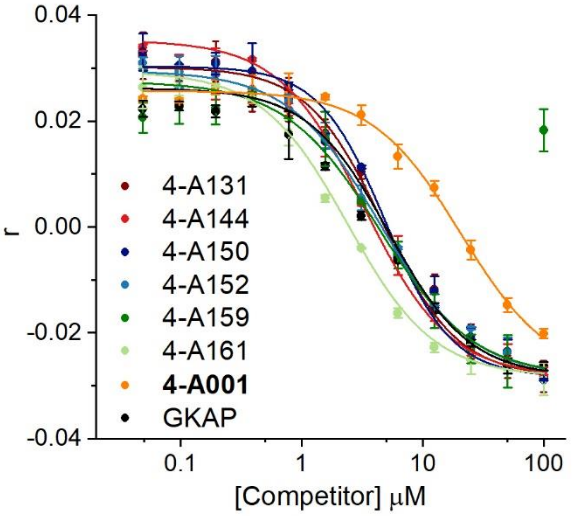

Competitor	IC_{50} (μM)
**GKAP**	4.5 ± 0.4
**4-A001**	19 ± 3
**4-A131**	4.8 ± 0.1
**4-A144**	3.7 ± 0.4
**4-A150**	5.1 ± 0.4
**4-A152**	4.5 ± 0.2
**4-A159**	4.2 ± 0.5
**4-A161**	**2.4 ± 0.1**

Figure S12. Fluorescence anisotropy competition assay and fitted IC_{50} values for GKAP PBM (Ac-EAQTRL-COOH) and the hybrid compounds selected for validation from the single point screens. Compound **4-A001** was incubated with 5 equivalents of aldehydes in 50 mM NH_4Ac, pH 6.5 10 mM aniline containing buffer at room termparture in the presence of protein for 24 hours, after which 10 nM FITC-Ahx-TRL-COOH was added as tracer. The final SHANK1 PDZ concentration was 1 μM. Error bars represent standard deviation of duplicate samples.

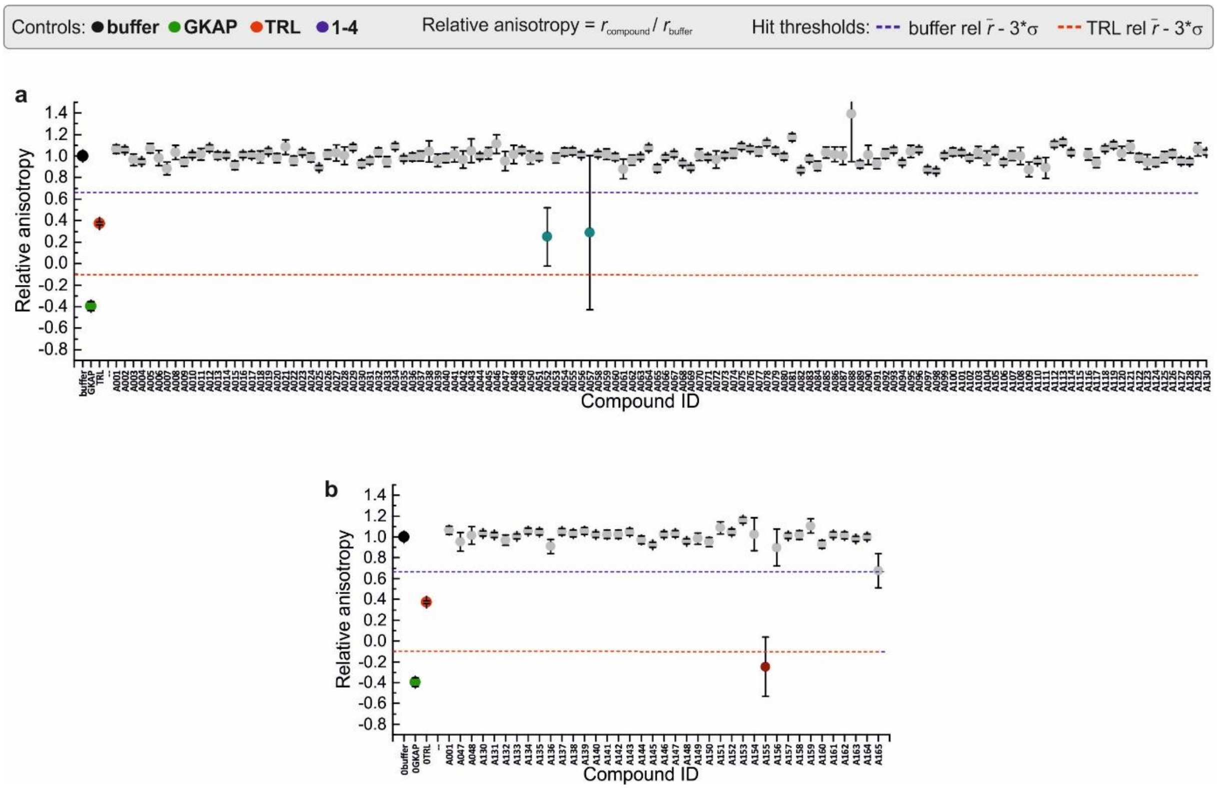

Figure S13. Results of the fluorescence anisotropy competition screening of the aldehyde fragments at 250 μM concentration. Aldehydes (A001-A165) were incubated for 24 hours in the presence of SHANK1 PDZ for 24 hours before tracer addition. Assay was performed in 50 mM NH_4Ac buffer, pH 6.5, 10 mM aniline using 1 μM final protein concentration and 10 nM FITC-Ahx-TRL-COOH as tracer at room temperature. All assays included a buffer (black), GKAP PBM (Ac-EAQTRL-COOH) (green) and TRL (red) control. Anisotropy is expressed relative to the buffer control, where no competitor is present. Each dot on the graph represents the relative activity of a an aldehyde fragment. Hit thresholds were defined as i) buffer r_{rel}-3σ (blue dashed line) and ii) TRL r_{rel}-3σ (red dashed line). Fragments reaching these thresholds are colored cyan and red respectively. Error bars represent the standard deviation of sample duplicates. A155 excluded from further screens due to its interference with fluorescence intensity.

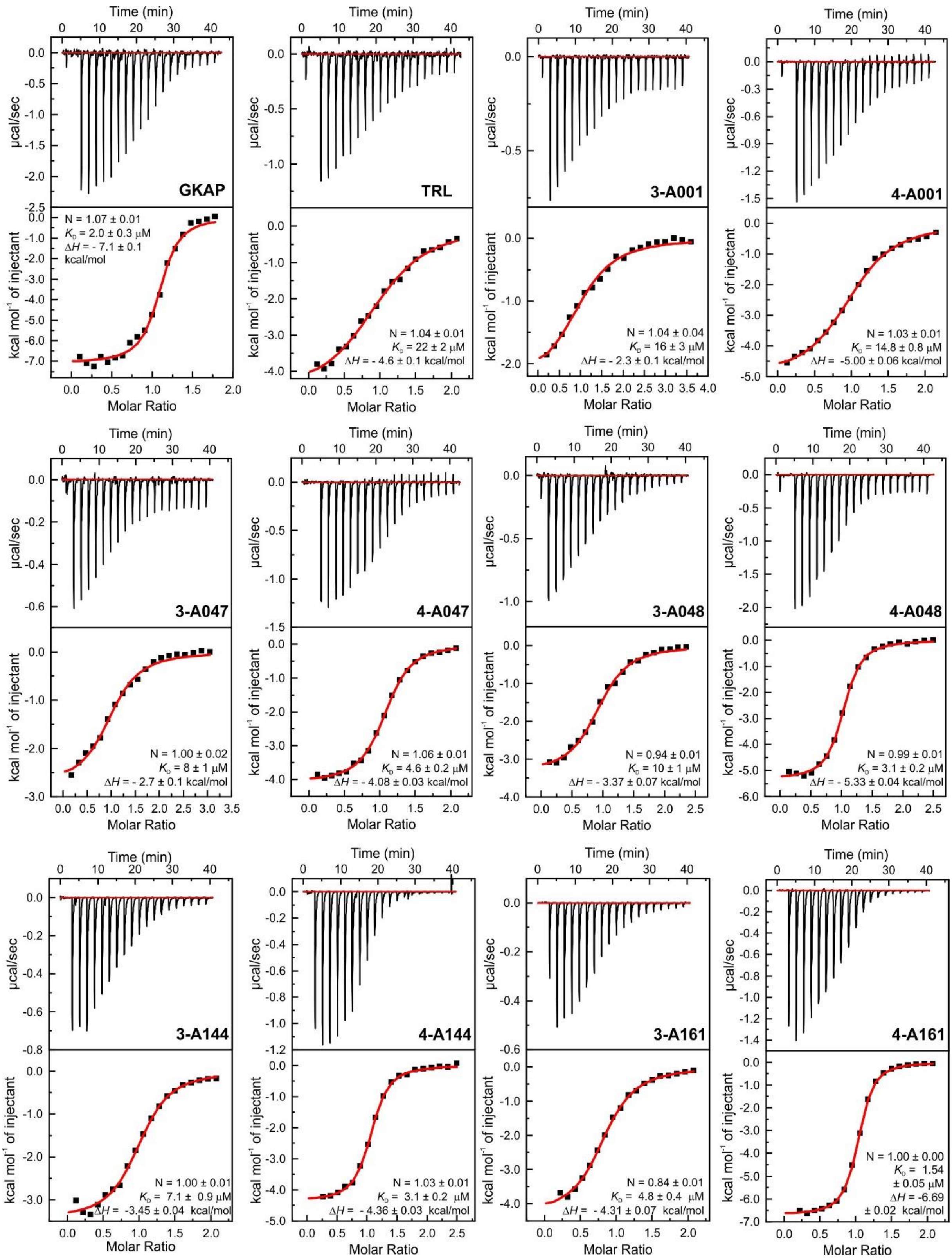

Figure S14. Raw ITC data (top) and fitted thermograms (bottom) for the tested hydrazone hits and controls. ITCs were measured in 25 mM Tris, pH 7.5 buffer containing 150 mM NaCl at 25 °C by injecting 0.75-1 mM peptide or hydrazone solution to 75-150 μM protein.

Table S2. Calculated group efficiency values (GE) for GKAP and the hydrazides relative to the core Ac-TRL-OH motif. (NnH = number of non-hydrogen atoms). The absolute configuration and enantiomeric purity of fragment A161 is unknown.

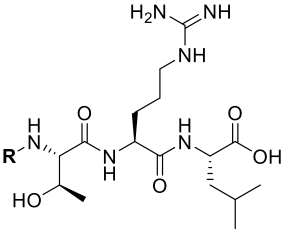

R = 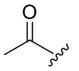

Ac-TRL-OH

Compound	R	ΔΔG (kJ/mol) ($\Delta G_{Compound}$-$\Delta G_{Ac\text{-}TRL\text{-}OH}$)	ΔN_{nH} ($N_{nH,Compound}$- $N_{nH,Ac\text{-}TRL\text{-}OH}$)	GE (-ΔΔG/ΔN_{nH})
**GKAP**	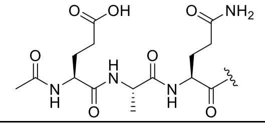	-6.03	23	0.26
**3-A001**	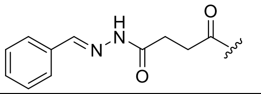	-0.84	12	0.07
**4-A001**	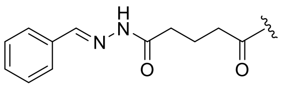	-1.08	13	0.08
**3-A047**	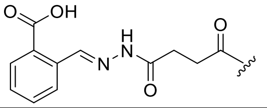	-2.65	15	0.18
**4-A047**	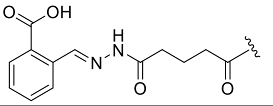	-2.05	16	0.13
**3-A048**	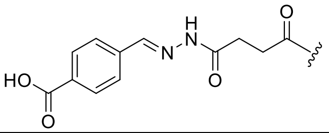	-3.98	15	0.27
**4-A048**	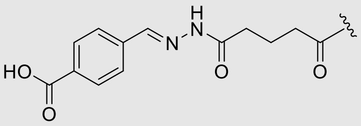	-4.98	16	0.31
**3-A144**	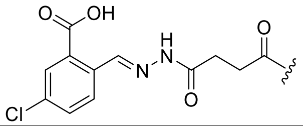	-2.89	16	0.18
**4-A144**	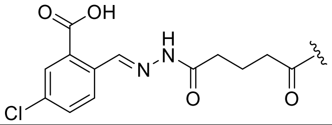	-3.87	17	0.23
**3-A161**	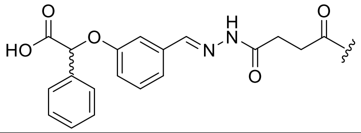	-4.94	23	0.21
**4-A161**	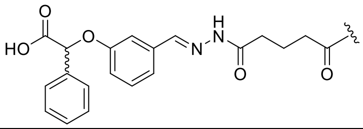	-6.69	24	0.28

Table S3. Calculated group efficiency values (GE) for hydrazides with the core compound **3-A001**. Added groups compared to the core compound highlighted red. The absolute configuration and enantiomeric purity of fragment A161 is unknown.

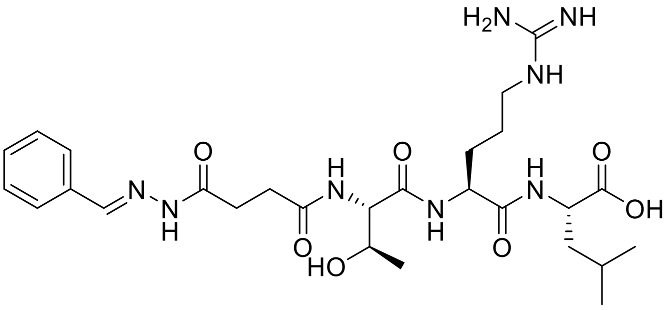

3-A001

**Compound**	**Added substituent (in red)**	**$\Delta\Delta G$ (kJ/mol)** **($\Delta G_{Compound}$-$\Delta G_{3\text{-}A001}$)**	**ΔN_{nH}** **($N_{nH,Compound}$-$N_{nH,\ 3\text{-}A001}$)**	**GE** **($-\Delta\Delta G/\Delta N_{nH}$)**
**3-A047**	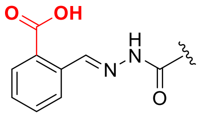	-1.81	3	0.60
**3-A048**	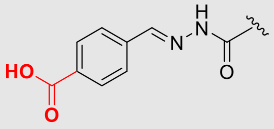	-3.14	3	1.05
**3-A144**	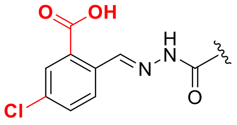	-2.05	4	0.51
**3-A161**	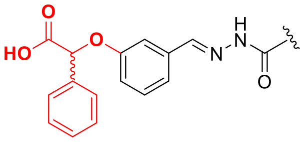	-4.10	11	0.37

Table S4. Calculated group efficiency values (GE) for hydrazides with the core compound **4-A001.** Added groups compared to the core compound highlighted red. The absolute configuration and enantiomeric purity of fragment A161 is unknown.

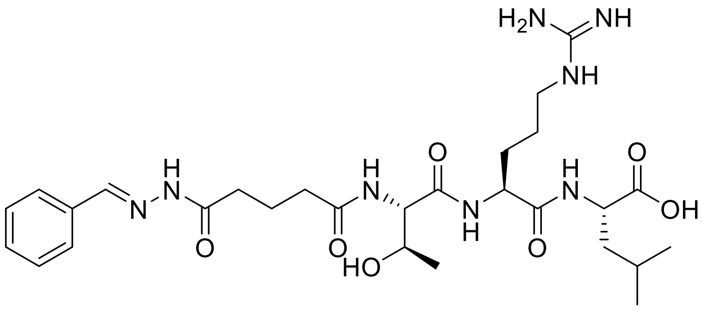

4-A001

Compound	Added substituent (in red)	ΔΔG (kJ/mol) ($\Delta G_{Compound}$-$\Delta G_{4\text{-}A001}$)	ΔN_{nH} ($N_{nH,Compound}$-$N_{nH,4\text{-}A001}$)	GE (-ΔΔG/ΔN_{nH})
**4-A047**	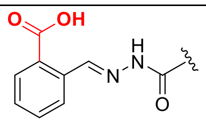	-0.97	3	0.32
**4-A048**	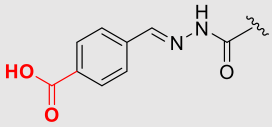	-3.90	3	1.30
**4-A144**	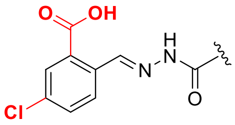	-2.79	4	0.70
**4-A161**	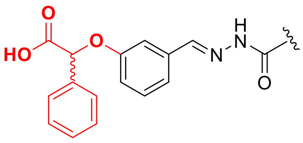	-5.61	11	0.51

Table S5. Statistics for obtained crystal structures.

	Ligand			
	**3-A047**	**4-A047**	**3-A048**	**4-A048**
**Reservoir conditions**	0.1 M HEPES pH 7<br>35% PEG 400	0.1 M HEPES 7.75<br>40% PEG 400<br>30% w/v Trimethylamine N-oxide dihydrate	0.1 M HEPES pH 7<br>35% PEG 400	0.2 M HEPES 7.75<br>40% PEG 400
**Data Collection**				
X-ray source	DLS Beamline i03	DLS Beamline i04-1	DLS Beamline i03	DLS Beamline i03
Processed using	xia2 (DIALS, Aimless)	Autoproc + Staraniso	xia2 (DIALS, Aimless)	xia2 (DIALS, Aimless)
oscillations	0.25	0.15	0.25	0.2
images collected	800	2400	800	1000
Space group	$P\ 2_1\ 2_1\ 2_1$	$P\ 2_1\ 2_1\ 2_1$	$P\ 2_1\ 2_1\ 2_1$	$P\ 2_1\ 2_1\ 2_1$
**Unit cell dimensions**				
a, *b*, *c*, (Å)	44.57, 65.97,86.90	44.67 65.91 85.64	44.30, 64.95, 88.28	45.43, 65.64, 86.35
α, β, γ (°)	90.0, 90.0, 90.0	90.0, 90.0, 90.0	90.0, 90.0, 90.0	90.0, 90.0, 90.0
Resolution	2.12 - 65.97 (2.12-2.16)	1.8- 52.23 (1.8 - 1.86)	1.57 - 88.28 (1.57 - 1.60)	1.62- 65.64 (1.62 - 1.65)
Observations	103336 (4553)	284711 (11510)	250100 (11477)	223892 (8301)
Unique reflections	15149 (734)	23236 (1163)	36380 (1782)	33616 (1510)
R_{merge} (I)	0.107 (1.355)	0.050 (1.838)	0.039 (1.291)	0.079 (1.120)
R_{meas} (I)	0.116 (1.479)	0.053 (1.939)	0.042 (1.406)	0.086 (1.240)
R_{pim} (I)		0.015 (0.612)	0.016 (0.550)	0.033 (0.522)
CC 1/2	0.996 (0.640)	1.000 (0.706)	0.999 (0.777)	0.998 (0.863)
I/σ	7.4 (1.2)	19.1 (1.2)	15.1 (1.1)	8.1 (1.0)
Completeness	100.0 (100.0)	92.16 (71.14)	100.0 (99.6)	99.7 (90.8)
Redundancy	6.8 (6.2)	12.3 (9.9)	6.9 (6.4)	6.7 (5.5)
**Refinement**				
Protein molecules in au	2	2	2	2
Rwork/Rfree (%)	21.43/24.61	22.24/25.59	20.53/23.11	20.97/24.71
No atoms	1795	1819	1926	1989
Protein	1622	1614	1657	1654
Ligand	97	92	90	92
Water	76	113	179	240
**Mean B factors (Å)**	54.8	45.0	43.7	36.7
Protein	54.1	43.3	42.5	35.2
Ligand	68.5	77.0	66.3	60.8
Water	53.5	43.4	50.0	44.0
**R.m.s. deviations**				
Bond length (Å)	0.008	0.008	0.006	0.006
Bond angles	0.87	0.86	0.72	0.68
**Ramahandran statistics**				
% favoured	98.5	99.0	99.5	100.00
% allowed	1.5	1.0	0.5	0
% outliers	0	0	0	0
Clashscore	3.25	5.66	2.00	3.15

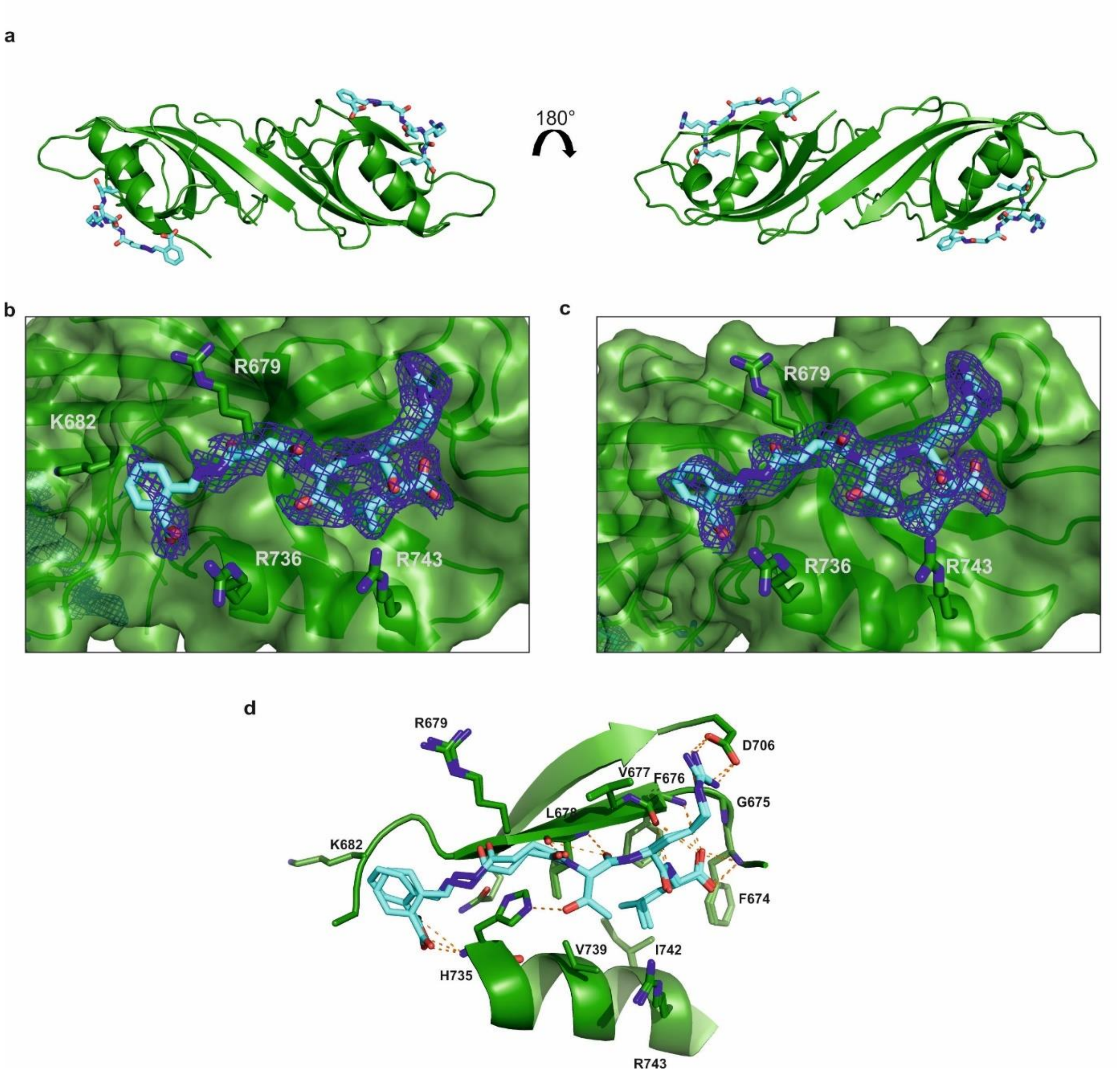

Figure S15. a) Co-crystal structure of SHANK1 PDZ (green) with compound **3-A047** (cyan) (PDB: 6YWZ) b-c) Ligand binding sites showing electron density map around the ligand at rmsd level of 0.5 (dark blue); adjacent positively charged side chains are labelled. d) Overlay of the two binding sites showing the two different ligand conformations. Polar contacts are indicated by orange dashed lines.

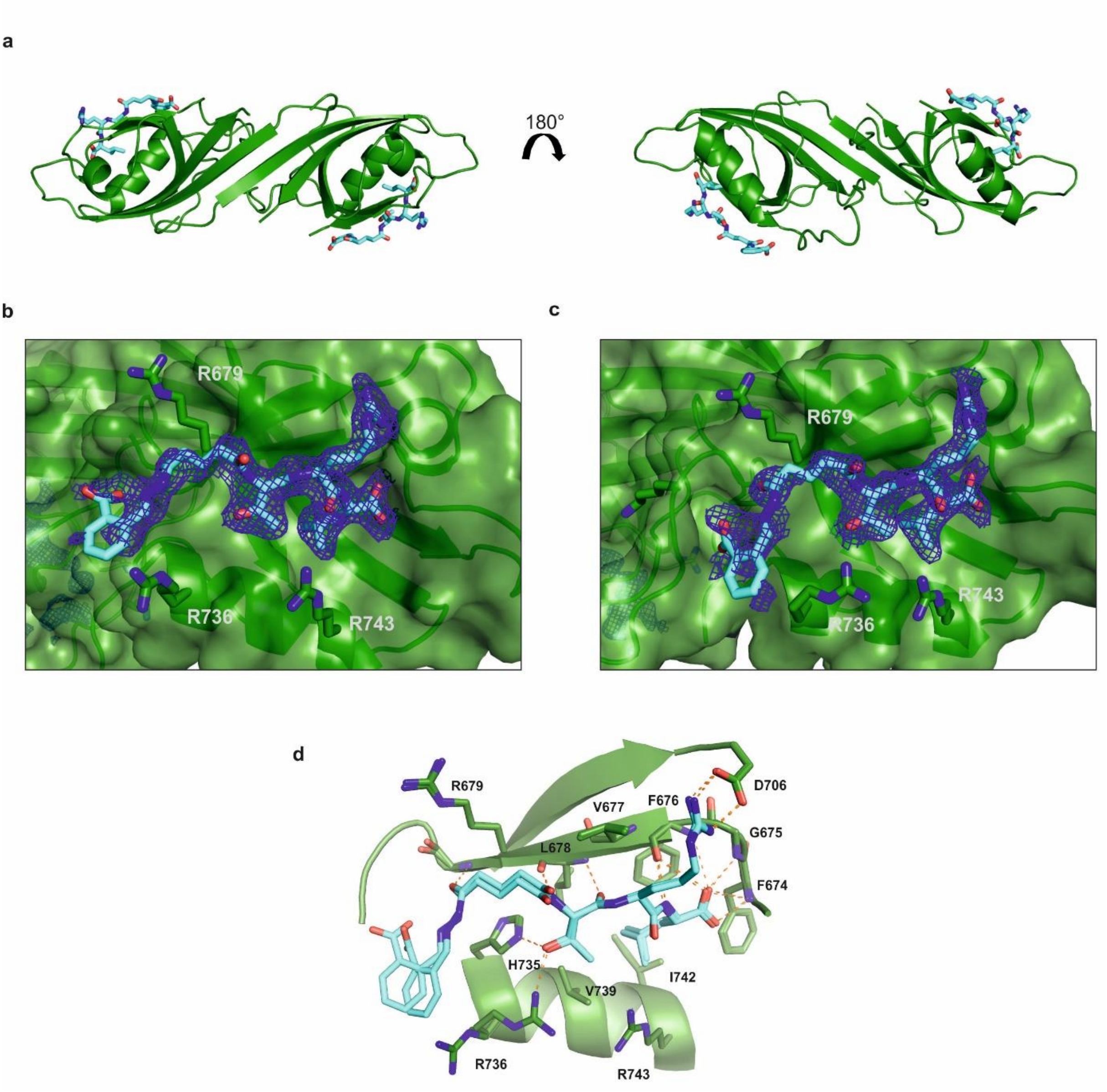

Figure S16. a) Co-crystal structure of SHANK1 PDZ (green) with compound **4-A047** (cyan) (PDB: 6YX1) b-c) Ligand binding sites showing electron density map around the ligand at rmsd level of 0.5 (dark blue), adjacent positively charged side chains are labelled. d) Overlay of the two binding sites showing the two different ligand conformations. Polar contacts indicated by orange dashed lines.

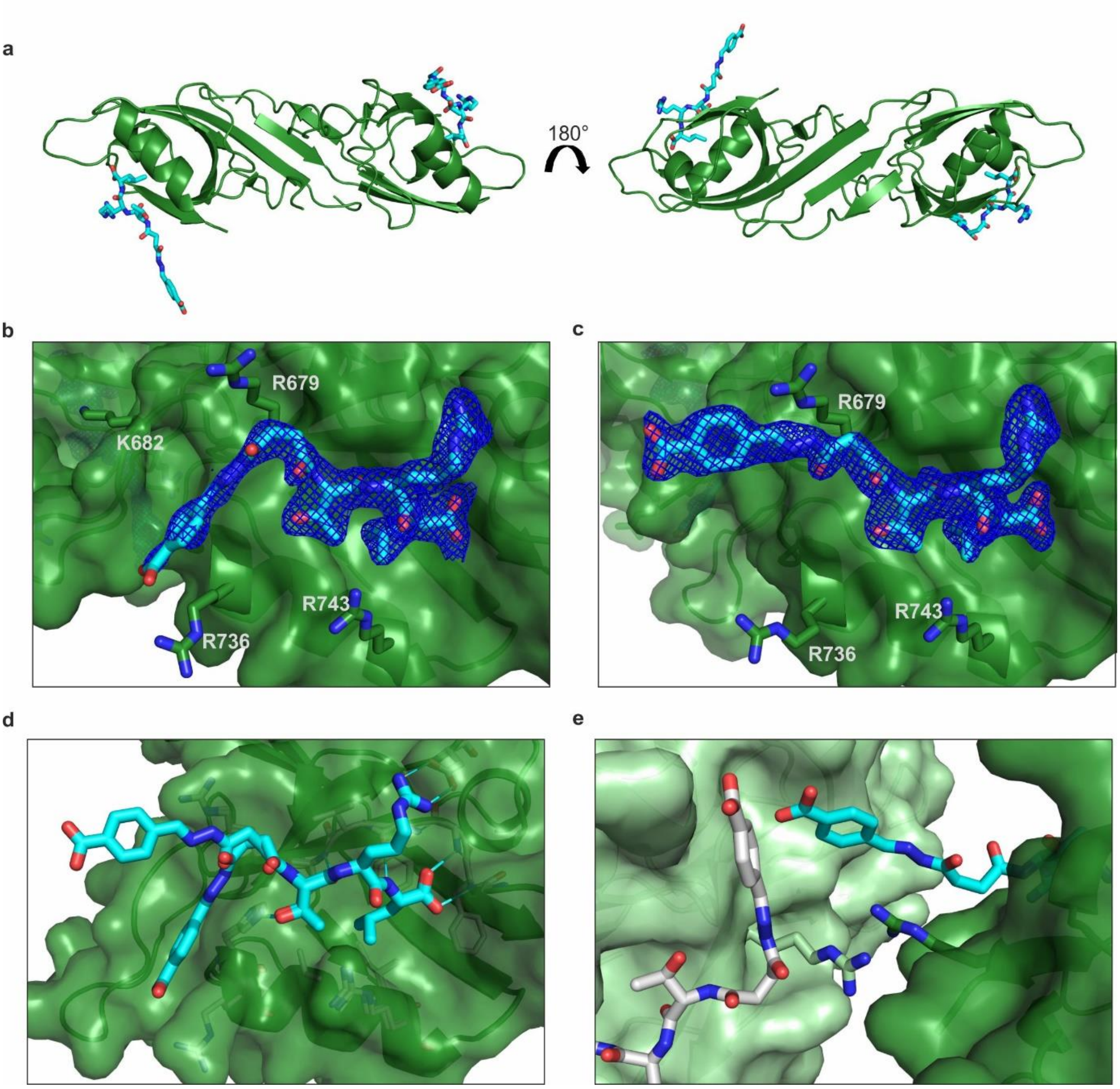

Figure S17. a) Co-crystal structure of SHANK1 PDZ (green) with compound **3-A048** (cyan) (PDB: 6YX0) b-c) Ligand binding sites showing electron density map around the ligand at rmsd level of 1.0 (dark blue), adjacent positively charged side chains are labelled. d) Overlay of the two binding sites showing the two different ligand conformations. e) Interface between symmetry related binding sites, showing that the conformation of the ligand is influenced by crystal packing.

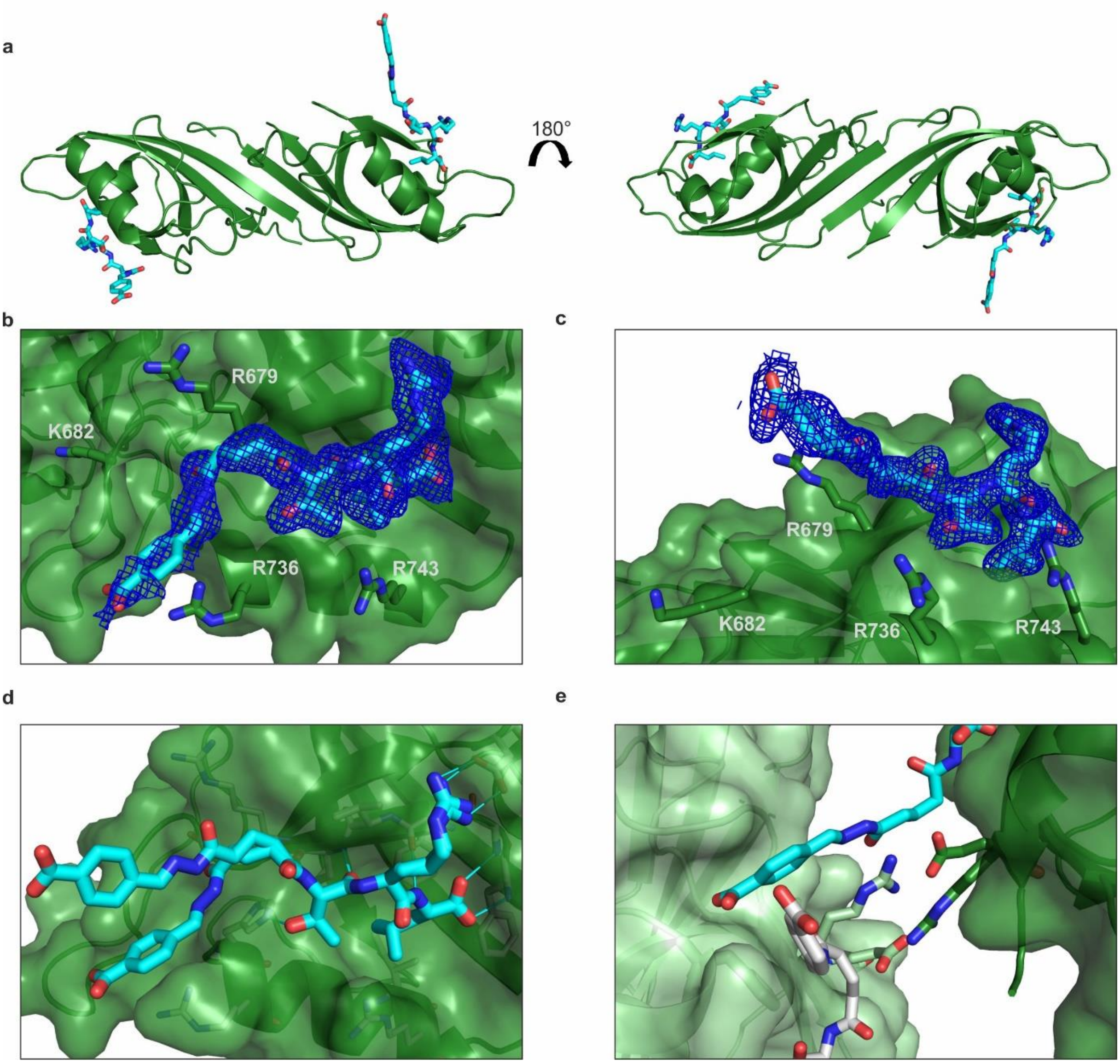

Figure S18. a) Co-crystal structure of SHANK1 PDZ (green) with compound **4-A048** (cyan) (PDB: 6YX2) b-c) Ligand binding sites showing electron density map around the ligand at rmsd level of 0.5 (dark blue), adjacent positively charged side chains are labelled. d) Overlay of the two binding sites showing the two different ligand conformations. e) Interface between symmetry related binding sites, showing that the conformation of the ligand is influenced by crystal packing.

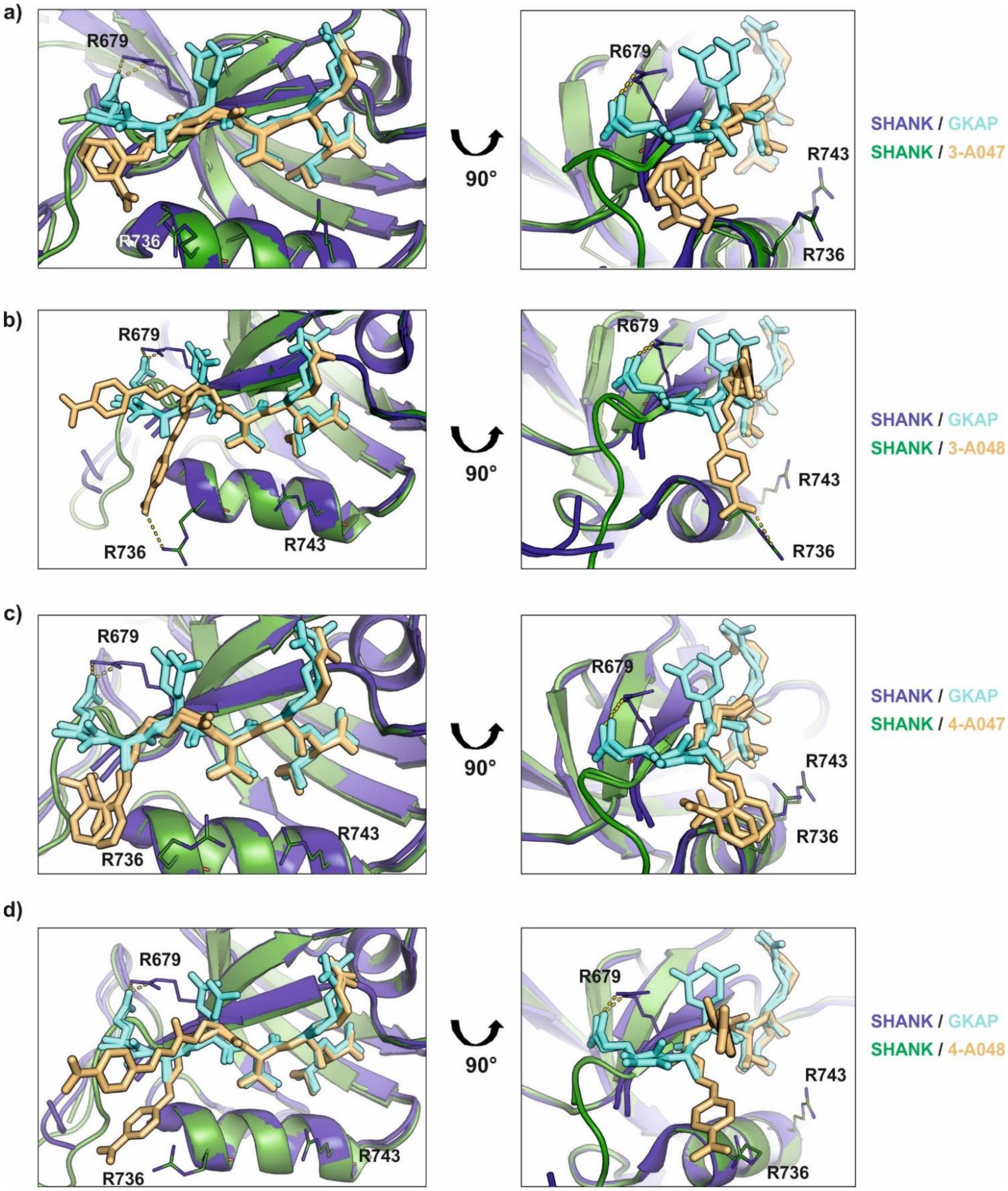

Figure S19. Overlays of co-crystal structures of GKAP (cyan) in complex with SHANK1 PDZ (blue, PDB:1Q3P) with a) **3-A047**; b) **3-A048**; c) **4-A047** and d) **4-A048** (orange) in complex with SHANK1 PDZ (forest green), chains A and B are superimposed in all crystal structures. Yellow dashed lines indicate polar contacts. Only relevant Arg side-chains are shown in the figure. The N-terminal glutamate residue of GKAP interacts with R679, in contrast, the carboxylic acid moiety of the bound hybrids exhibits a preference for interaction with the opposite binding site, making an interaction with R736 of SHANK PDZ.

Table S6. RMSD values of crystal structures after alignment with ShankPDZ/GKAP complex (1Q3P). Alignment was performed pairwise to full protein chains (chain A and B separately) and TRL-OH residues of the ligands.

Compound	*PDB*	*RMSD Full protein-chain*		*RMSD TRL-OH residues*	
		Chain A	Chain B	Chain A	Chain B
**3-A047**	6YWZ	0.411	0.452	0.316	0.231
**3-A048**	6YX0	0.353	0.469	0.211	0.183
**4-A047**	6YX1	0.416	0.519	0.251	0.287
**4-A048**	6YX2	0.353	0.484	0.146	0.199

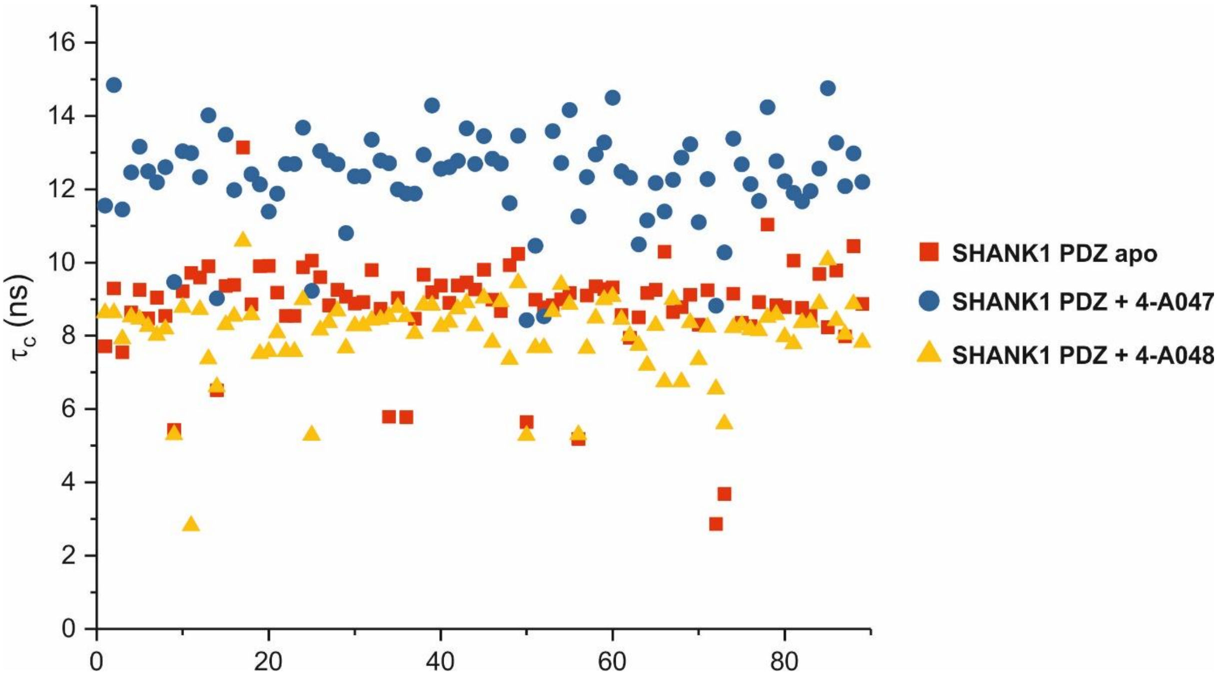

Figure S20. Fitted rotational correlation time of the SHANK1 residues with and without ligands at 0.32 mM protein concentration using 1:2 protein:ligand ratio in 5 mM Tris, 100 mM NaCl, pH 7.4. The average τ_c of SHANK1 8.8 ns corresponds to approximately 13 kDa, and is similar for SHANK1 in the presence of **4-A048** (τ_c = 8.1 ns). The τ_c of SHANK1 in the presence of **4-A047** is higher, (12.4 ns) which corresponds to a protein of approximately 19 kDa, which is below what would be expected if dimerization were to occur. SHANK1 has been observed to dimerize in the solid state through interactions of protomers via β-strand distal to the ligand binding cleft[2]; we conclude that this is likely a crystallographic artefact or only occurs at higher concentrations that those used for these NMR analyses and differs from the interaction of the ligand with a symmetry related protein observed in this work (Figure S18).

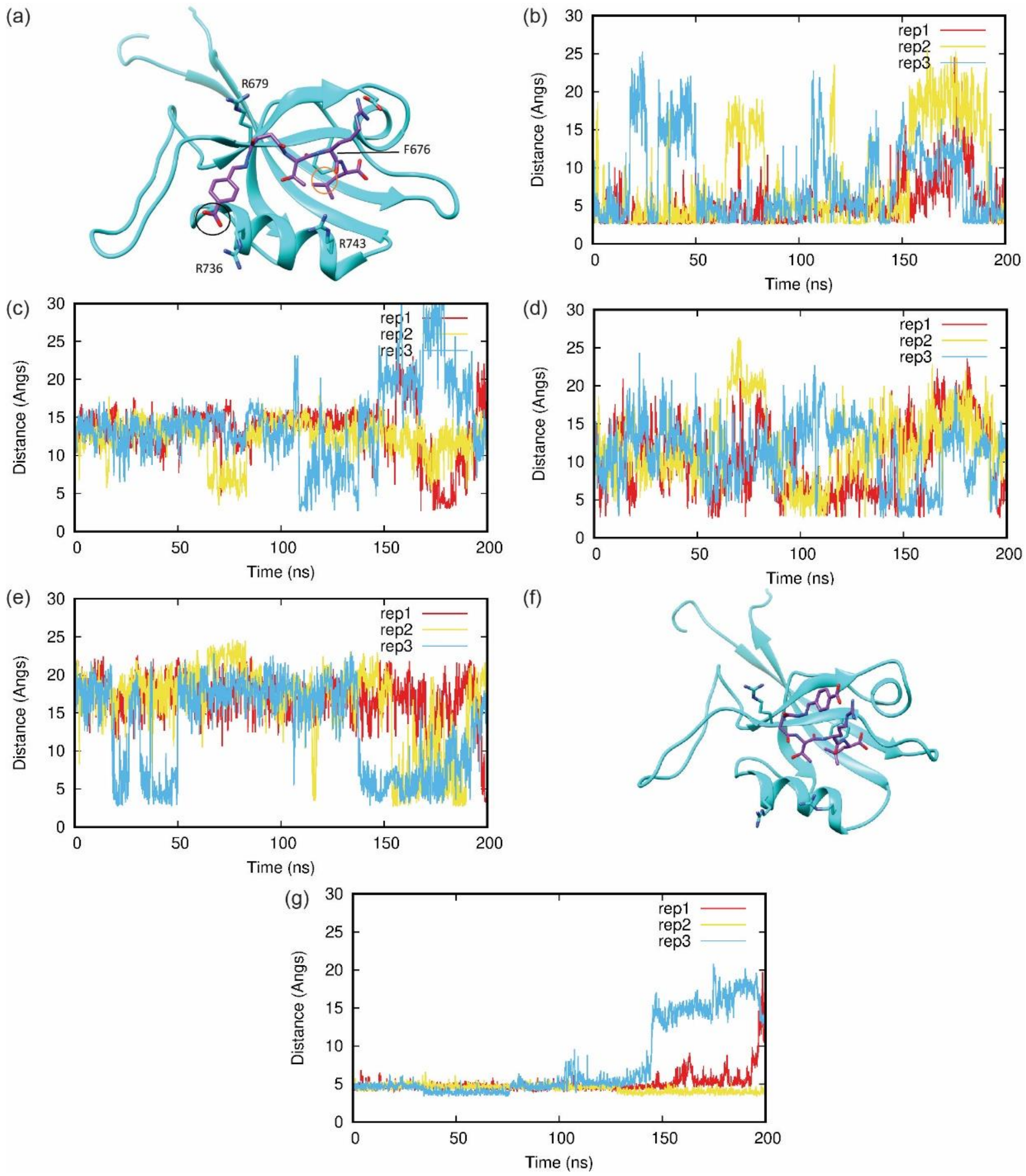

Figure S21. Molecular dynamics simulations for the the **3-A048**/SHANK1 interaction (n = 3), illustrating distances between the aromatic carboxylate of **3-A048** and arginine residues on SHANK1 or the ligand (see also Moive S1); these data provide a further rationale for the disorder in this region observed in the x-ray structures; (a) Binding pose at the start of the simluations showing the positions of: the arginine sidechains in the distance calculations between the aromatic carboxulate (circled black) of **3-A048**; F767 of SHANK1-PDZ and the methyl C2 of Val in **3-A048** (circled orange); (b) Distance fom R736 to aromatic carboxylate of **3-A048** (c) Distance fom R679 to aromatic carboxylate of **3-A048** (d) Distance fom R743 to aromatic carboxylate of **3-A048** (e) intramolcular distance fom aromatic carboxylate of **3-A048** to arginine of **3-A048** (f) diagram illustrating intramolecular interaction between aromatic carboxylate of **3-A048** to arginine of **3-A048** (g) distance between F767 of SHANK1-PDZ and the methyl C2 of Val in **3-A048**. Collectively the simulations indicate that interaction between R736 and the aromatic carboxylate of **3-A048** is generally stable, that the side-chains of R679 and R743 seem to reciprocate for attracting the carboyxlate and that when the carboxylate folds over it can interact with its own guanidinium group on the ligand, pushing out R679.

PSD 95 PDZ1 amino acid sequence

```
         10         20         30         40         50         60
GAMEMEYEEI TLERGNSGLG FSIAGGTDNP HIGDDPSIFI TKIIPGGAAA QDGRLRVNDS
         70         80         90
ILFVNEVDVR EVTHSAAVEA LKEAGSIVRL YVMRRKPPA
```

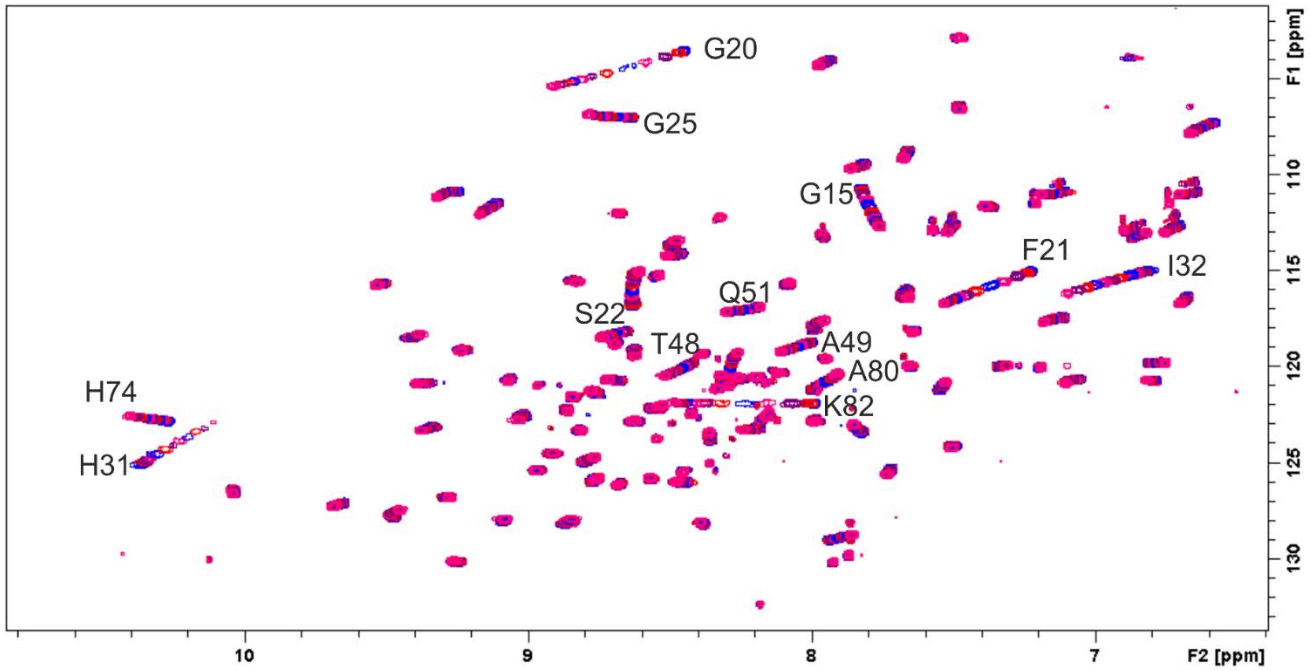

Figure S22. Overlay of plots for **4-A047** with Ligand:protein ratio ranging from 1:1 to 100:1. The gradual change in chemical shifts of limited residues indicate that the binding of the ligand is specific and to the putative ligand binding site.

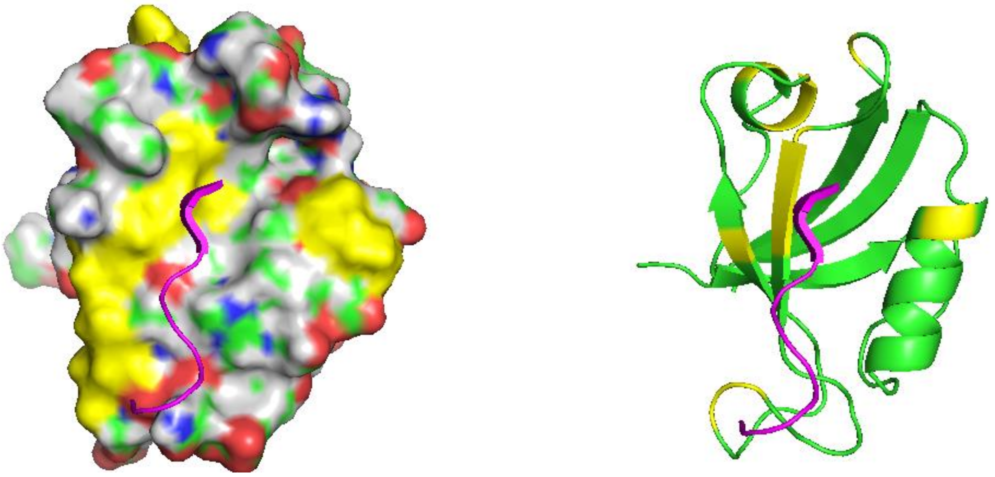

Figure S23. Mapping of chemical shift changes onto the structure of PSD95 PDZ1 using PDB Accession Code 2KA9 (cypin peptide in magenta). Residues whose chemical shifts are affected are coloured yellow; these residues line the binding site.

Table S7. Concentration dependent shift changes of compound **4-A047** for a few residues whose chemical shifts have been assigned

Concentration (µM)	^{1}H Chemical Shifts (ppm)				
	K82	G20	F21	I32	H31
0	7.99	8.448	7.225	6.811	10.37
72	8.01	8.466	7.24	6.83	10.349
327	8.0674	8.516	7.27	6.84	10.344
936	8.16	8.583	7.32	6.857	10.328
1512	8.23	8.652	7.365	6.875	10.309
2592	8.32	8.72	7.42	6.91	10.278
3528	8.37	8.773	7.453	6.936	10.251
4392	8.4	8.812	7.478	6.967	10.22
5184	8.44	8.838	7.494	6.992	10.20
5976	8.46	8.861	7.508	7.02	10.17
6912	8.47	8.885	7.521	7.057	10.15
7776	8.47	8.9	7.53	7.091	10.10
EC_{50} fitted (µM)	2433±289	4609± 678	3145± 262	Cannot be fitted	Cannot be fitted

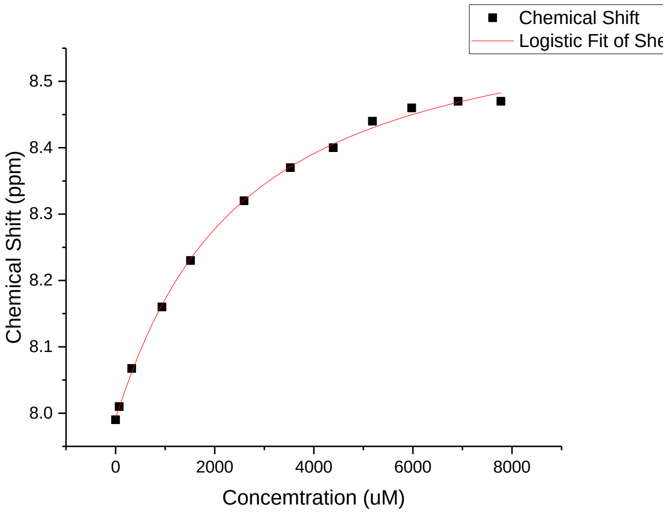

Figure S24. Residue K82: EC_{50}=2433±289 µM. A simple logistic fit is used to get this EC_{50} value.

Table S8. Relative affinities using magnitude of shift changes of residues whose chemical shifts are significantly affected

Residues							
Top shift changes seen in these residues	**4-A047**	**3-A048**	**4-A048**	**3-A144**	**4-A144**	**4-A161**	**GKAP**
G15	0.096	0.075	0.109	0.028	0.130	0.022	0.08
G20	0.163	0.121	0.184	0.043	0.218	0.035	0.133
F21	0.124	0.097	0.142	0.035	0.176	0.028	0.106
S22	0.087	0.069	0.101	0.025	0.123	0.022	0.066
G25	0.037	0.025	0.042	0.01	0.039	0.011	0.057
H31	0.067	0.046	0.051	0.035	0.070	0.035	0.068
I32	0.049	0.035	0.039	0.022	0.052	0.026	0.049
T41	0.018	0.013	0.016	0.0055	0.026	0.0037	0.015
A48	0.041	0.033	0.048	0.013	0.06	0.0099	0.035
A49	0.042	0.031	0.050	0.011	0.058	0.0099	0.038
Q51	0.034	0.027	0.039	0.01	0.048	0.0073	0.031
A80	0.059	0.042	0.026	0.014	0.02	0.012	0.044
K82	0.169	0.123	0.190	0.039	0.229	0.032	0.138
Affinities relative to **6 (4-A047)***		Slightly weaker	Slightly stronger	Weakest	Possibly strongest	Weakest	Slightly weaker

* Although the relationship between shift magnitude and affinity should be used cautiously, it is valid here as we are evaluating a well established interaction using a series of structurally and closely related ligands, considered as binding in a specific way.

2. Materials and methods

2.1. Peptide Synthesis and purification

2.1.1. General methods

All Fmoc-protected amino acids and coupling reagents were purchased from Novabiochem (Merck) or from Fluorochem. DMF used for couplings was ACS grade from VWR. Peptides were synthesized either manually or using microwave assisted synthesis.

Manual couplings were performed using solid phase peptide synthesis using 5 equivalent amino acid excess unless otherwise stated, using HCTU (5 eq) and DIEA (10 eq) as coupling reagents and base in a fritted SPE tube, shaking the coupling mixture at room temperature for 2 hours or overnight.

Microwave assisted synthesis was performed on CEM Liberty Blue peptide synthesizer using 5 equivalent amino acid excess and DIC (5 equivalent) and Oxyma (5 equivalent) as coupling reagents. Single couplings were performed at 90 °C for 60s. Arginine was double coupled at 75 °C for 5 minutes. Deprotection solution was 20% piperidine in DMF. Coupling efficiency was monitored using UV absorbance after Fmoc deprotection.

N-terminal acetylation of the peptides was performed manually in a fritted SPE tube using 50 equivalents acetic anhydride and DIPEA in DMF: DCM 1:1 (1ml) for 2 x 30 minutes. Peptides were cleaved using TFA: TIPS: water 90:5:5 (w/w) mixture for 3 hours. After cleavage the TFA was evaporated and the residual was dissolved in DMSO.

Preparative purifications were performed using RP-HPLC on an Agilent 1260 Infinity HPLC equipped with a diode array and a mass-detector using a Kinetex EVO C18 (250mm x 21.2 mm, Phenomenex) column at 10 ml/min flow rate. Eluents were A: 0.1% formic acid in H_2O and B: 0.1% formic acid in acetonitrile using a gradient of 5-35 or 5-45% B over 35 minutes. Collected fractions were analysed using LC-MS (Eluents were A: 0.1% formic acid in H_2O and B: 0.1% formic acid in acetonitrile), pure fractions were freeze dried. To asses purity analytical UV-HPLC were performed on Agilent 1260 infinity HPLC. High resolution mass spectrometry was performed on Bruker maXis II™ ESI–QTOF mass spectrometer.

2.1.1. Synthesis of peptide hydrazides

C-terminal peptide hydrazides (**1-2**) were synthesized on Fmoc-hydrazono-pyruvyl-aminomethylpolystyrene resin (Iris Biotech) on 0.1 mmol scale. The first amino acid was coupled manually overnight and the synthesis was continued on CEM Liberty Blue peptide synthesizer, peptides were acetylated and cleaved as described above.

For peptides containing hydrazide on the N-terminus (**2-3**), first the TRL sequence was synthesized manually on Fmoc-Leu-Wang resin as described above. For compound **2** with the C2 linker 4-(N'-tert-butoxycarbonyl-hydrazino)-4-oxo-butyric acid (1.2 equivalents) was dissolved in DMF together with 1.2 equivalents HCTU and added to the resin, to which 2 equivalents of DIPEA was added and coupled at room temperature for 3 hours. For compound **3** with the C3 linker 4-(N'-tert-butoxycarbonyl-hydrazino)-4-oxo-propionic acid (1.2 equivalents) was dissolved in DMF together with 1.2 equivalents HCTU and added to the resin, to which 2 equivalents of DIPEA was added and coupled at room temperature for 3 hours (Scheme S1). Coupling was repeated twice, followed by cleavage using TFA:TIPS:water 90:5:5 (w/w) mixture for 3 hours. TFA was evaporated and crude hydrazides were dissolved in DMSO.

In order to yield hydrazones (**1-A001, 2-A001, 3-A001, 3-A047, 3-A048, 3-A144, 3-A161, 4-A001, 4-A047, 4-A048, 4-A144, 4-A161**) approximately 20-fold excess of the corresponding aldehyde fragment was added to the crude peptide hydrazides in DMSO and hydrazone formation was followed by LCMS. After completion, the formed hydrazones were purified by RP-HPLC. In order to maintain stable hydrazones the freeze dried compounds were dissolved in either 20 mM Tris, 150 mM NaCl, pH 7.5 or in 50 mM NH_4Ac, pH 7.5 buffer.

2.2. Protein expression and purification

Human Shank1 PDZ domain (656-762) was cloned into the pGEX-6P-2 expression vector and transformed to BL21 Gold cell line. 10 ml overnight starter culture was inoculated to 1 L 2xYT media containing 50 μg/ml chloramphenicol. Cells were grown at 37 °C until OD_{600} 0.6-0.8 and induced with 0.1 mM IPTG and incubated overnight at 18 °C. Cells were harvested and resuspended in 20 mM Tris, pH 8, 500 mM NaCl, containing protease inhibitor and 1U of DNase I per litre of cell culture. Cells were lysed by sonication (8 cycles, 20 seconds on 40 seconds off, 10 μA) and centrifuged at 25,000 g for 45 minutes at 4 °C. The supernatant was filtered

(0.22 μm membrane) and applied to glutathione beads and washed with 10 CV 20 mM Tris, pH 8, 500 mM NaCl. GST was cleaved on-column overnight at 4 °C using Prescission protease. The eluted fractions were concentrated and purified by size-exclusion chromatography on S75 26/60 pg column in 20 mM Tris, 150 mM NaCl, 5% glycerol pH 7.5 buffer. Collected fractions were analysed by SDS-PAGE and concentrated. Pure protein was analysed by high resolution mass spectrometry: expected m/z: 12326.3 measured m/z: 12325.6. Concentration was determined by UV-VIS spectroscopy using the 8480 M^{-1} cm^{-1} extinction coefficient.

PSD-95 PDZ1 was prepared as described in Volgrig *et. al* (2013) ACS Chem Biol, 8: 2209-2216.[3]

2.3. Aldehyde library selection

A diverse subset of fragments was selected from commercially available small molecule aldehydes (Aldrich[CPR,] Fluorochem and Acros). Available compounds having one aldehyde functionality were filtered first based on physicochemical properties: 80 Da < MW < 400 Da, HBA, HBD ≤ 3, and clogP ≤ 5. To select a diverse set of compounds, first, binary molecular fingerprints were generated for each molecule using the *FragFp* descriptor implemented in DataWarrior (openmolecules.org), which relies on a predefined substructure fragment library and is used to compare 2D structural similarities. Then, using the same software, structurally dissimilar subset of compounds was selected based on the molecular fingerprints. Compounds were further excluded based on undesired functional groups and commerical availability, which resulted in a library of 165 aldehydes including a subset of 36 compounds having carboxylic acid functional groups.

2.4. Fluorescence anisotropy

2.4.1. General comments

Fluorescence anisotropy assays were performed in 384-well plates (Greiner Bio-one) and the fluorescence anisotropy measured using a Perkin Elmer EnVisionTM 2103 MultiLabel plate reader, with excitation at 480 nm (30 nm bandwidth), polarised dichroic mirror at 505 nm and emission at 535 nm (40 nm bandwidth, S and P polarised) at a controlled temperature of 25 °C. Fluorescence anisotropy data was processed as described previously.[4] The data from both

the P (perpendicular intensity) and S (parallel intensity) channels, resulting from this measurement and corrected by subtracting the corresponding control wells, were used to calculate the intensity and anisotropy for each well following Equations 1 and 2:

$$I = (2PG)+S \quad Equation\ 1$$

$$r = (S-PG) \quad Equation\ 2$$

r = anisotropy, I = total intensity, P = perpendicular intensity, S = parallel intensity, G is an instrument gain factor.

2.4.2. Direct titration

Assays were performed in 50 mM NH_4Ac, 10 mM aniline, pH 6.5 buffer. Shank 1 PDZ was dialysed into the assay buffer prior to the experiment. Fluorescence anisotropy direct titration assays were performed with protein concentration diluted over 24 points using ½ dilutions. 20 µL of buffer were first added to each well then 20 µL of protein solution was added to the first column. The well solution was mixed and 20 µL was taken out and added to the next column, which resulted in a serial dilution of the protein across the plate, starting from 50 µM final protein concentration. Finally, 20 µL of tracer (FITC-Ahx-TRL-COOH) was added to the wells. For control wells, the tracer peptide was replaced with an identical volume of assay buffer and plates were read immediately, and after an hour or after 24 hours.

$$L_b = (r-r_{min})/\lambda(r_{max}-r)+r-r_{min} \quad Equation\ 3$$

$$y = \{(k+x+[FL])-\surd\{k+x+[FL]^2-4x[FL]\}\}/2 \quad Equation\ 4$$

r = anisotropy, I = total intensity, P = perpendicular intensity, S = parallel intensity, L_b = fraction ligand bound, λ = Ibound/Iunbound = 1, [FL] = concentration of fluorescent ligand, k = K_d and x = [added titrant], G is an instrument gain factor.

2.4.1. Competition assay

Experiments were performed in either 50 mM NH_4Ac, pH 6.5 buffer containing 10 mM aniline. FA competition assays were performed with the concentration of the competitior starting 100 µM, which was serially diluted over 12 points in a 1/2 regime with fixed protein

and tracer concentrations. 20 μL of protein and 20 μL of FITC-Ahx-TRL-COOH tracer were added to each well to give a final concentration of 1 μM and 10 nM respectively, matching the concentrations with the screening conditions. For control wells, the tracer peptide was replaced with an identical volume of assay buffer. The total volume in each well was 60 μL. Plates were read immediately after incubation at room temperature. The calculated average anisotropy values and their standard deviation were then fitted to a sigmoidal logistic model (Equation 5) using OriginPro 9.0. which provided the IC50 and error values.

$$y = r_{max} + (r_{min}\text{-}r_{max})/(1+(x/x_o)^p) \qquad Equation\ 5$$

2.4.2. Library screening

Screening was performed using fluorescence anisotropy competition assay, in 50 mM NH_4Ac pH 6.5 buffer containing 10 mM aniline. Each sample was prepared in duplicates. Screening was performed at 10 or 50 μM concentration of **1-4** using 5-fold excess of aldehydes in each well, with 1 μM protein and 10 nM final tracer concentration. Aldehyde stock solutions were prepared in DMSO at 100 mM concentration and pipetted into a 96-well plate, which then was further diluted into the assay buffer to the appropriate concentration. First, 10 μL hydrazone **1-,2-,3-,4-A001** and control peptides GKAP and TRL (from a 300 or 60 μM stock solution) was added to each well, which was followed by the addition of 10 μL of aldehyde solutions (from 1.5 or 0.3 mM stock). Then 20 μL SHANK1 PDZ protein was added to each well from a 3 μM stock solution. The plate was sealed and hydrazone formation was allowed to proceed at room temperature for 24 hours. After 24 hours 20 μL FITC-Ahx-TRL-COOH was added to the experiment wells or equal amount of buffers was added to the blanks and the plate was read immediately. All plates were prepared with the following controls: i) negative control without any competitor, ii) positive controls: GKAP, TRL iii) starting compounds without aldehydes. Aldehydes were tested on their own without having hydrazides in the solution to exclude any effect on fluorescence intensity.

Anisotropy was calculated as described above, duplicates were averaged. Anisotropies were normalized relative to the negative buffer control. Two different threshold values were used to assess hit compounds, which was calculated based on the average relative anisotropy calculated for the negative control or the TRL control peptide - 3 x standard deviation.

2.5. Isothermal tritration calorimetry

ITC experiments were carried out using Microcal ITC200i instrument (Malvern) at 25°C in 20 mM Tris, 150 mM NaCl, pH 7.5 buffer. Shank1 PDZ was dialysed against the buffer prior to experiment, lyophilized peptides and hydrazones were dissolved in the same buffer. 75 or 150 μM Shank1 PDZ was present in the cell and titrated with 0.8 – 1.5 mM peptide solutions, depending on compound solubility, from the syringe using 2 μL injections with 120 s spacing between the injections for 20 injection. Heats of peptide dilution were measured by titrating the peptide into buffer and subtracted from each measurement. Data was analysed using Microcal Origin 8 and fitted to a one-binding site model.

2.6.Co-crystallization

SHANK1 PDZ was incubated with ligands at a 1:1.2 to 1-1.5 molar ratio in 25 mM Tris, 150 mM NaCl pH 7.5 buffer having 10.5 mg/ml final protein concentration. Sparse matrix screening using the JCSG Core suites (Qiagen) was performed with the sitting-drop vapor-diffusion method at 20 °C. Protein was mixed with crystallization solution at a 1:1 ratio with final drop volume of 0.2 μl. Initial hit conditions were further optimized by screening HEPES pH 7 – 8.25 in 0.25 pH steps and PEG 400 concentration from 25-40% in 5% steps, crystals grew within 2-3 weeks. Crystals were flash cooled in liquid nitrogen without addition of any further cryorprotectant and sent to Diamond Light Source (DLS) for data collection. Data were collected at 100 K. For compounds **3-A047, 3-A048**, and **4-A048** data were processed with the xia2[5] bundle using DIALS[6] for integration and Pointless[7], Aimless[8] for scaling and merging. For compound **4-A047**, data was processed using autoPROC[9], XDS[10], Aimless[8] and STARANISO[11]. Phasing was performed by molecular replacement using Phaser[12] and using Chain A from 1Q3O.pdb as a search model. Initial refinement was done using REFMAC[13] with model building in COOT[14] using the CCP4i2[15] software package. Further refinements and TLS refinement were done in PHENIX[16] and the structures were analysed by Molprobity[17]. CIF files for the ligands were generated by AceDRG[18] implemented in CCP4i2.

2.7. Molecular dynamics simulations

All simulations were performed as NPT ensembles at 310 K using periodic boundary conditions. The simulations were based on the crystal structure of 3-A048-SHANK complex. Hydrogens, consistent to pH7, were added to the complex. Short range electrostatic and van der Waals' interactions were truncated at 1.4 nm while long range electrostatics were treated with the particle-mesh Ewald's method and a long-range dispersion correction applied. A simulation box extending 2 nm from the protein was filled with TIP3P waters and 150 mM Na and Cl ions added to attain a neutral charge overall. Pressure was controlled by the Berendsen barostat and temperature by the V-rescale thermostat. The simulations were integrated with a leap-frog algorithm over a 2 fs time step, constraining bond vibrations with the P-LINCS method. Structures were saved every 0.1 ns for analysis in each of the three repeat runs over 200 ns. Simulation data were accumulated on the Bristol BrisSynBio supercomputer Bluegem. Analysis: RMSF (Root Mean Square Fluctuation) calculations to determine the distances between the compound's aromatic carboxylate and side chain (and its own) guanidinium groups, over the course of the simulations, was carried out using the pairdist function in GROMACS tools.
Software: The GROMACS-2019.2 suite of software was used to set up and perform the molecular dynamics simulations and analyses. Molecular graphics manipulations and visualisations were performed using VMD-1.9.1 and Chimera-1.10.2 and movies made using VMD. **3-A048** was parametrised by Acpype.[19]

2.8. NMR relaxation experiments

All NMR experiments were carried out using Bruker Avance NMR spectrometers operating at 750 MHz ^{1}H frequency. Temperature was maintained at 298 K. Data were processed using Topspin and analysed in Sparky[20]. ^{15}N T_1 and T_2 values and ^{15}N heteronuclear NOE values were obtained from standard experiments[21] recorded at 750 MHz ^{1}H frequency on 0.32 mM samples (in 5 mM Tris, 100 mM NaCl, pH 7.4) of SHANK1 PDZ, at 1:2 protein:ligand ratio. 0, 50, 90, 190, 390, 590, 790, 1090, 1390, 1790 ms delays and 33.9, 67.8, 101.8, 135.7, 169.6, 203.5, 237.4, 271.4, 339.2, 407.0 ms delays were used respectively for T1 and T2 experiment.

2.9. PSD95 PDZ1 Ligand Experiments

NMR spectra were acquired on Bruker Avance III 700 MHz spectrometer equipped with [^{1}H, ^{15}N, ^{13}C]-cryoprobe. 1D ^{1}H and 2D ^{1}H-^{15}N HSQC spectra of PSD95 PDZ1 were aquired using 100μM protein dissolved in 20 mM phosphate pH 6.5 at 298K. Ligand titrations were performed by sequential addition of small aliquots of a concentrated sample of the stock ligand dissolved in the same buffer as the protein. All NMR data were processed using TopSpin 3.62; curve fitting was performed using Origin 2016 Software.

2.10. Compound characterization

GKAP PBM

Ac-EAQTRL-OH

Exact Mass: 758.3810

Expected $[M+H^+]^{1+}$	Measured $[M+H^+]^{1+}$
759.3810	759.3994

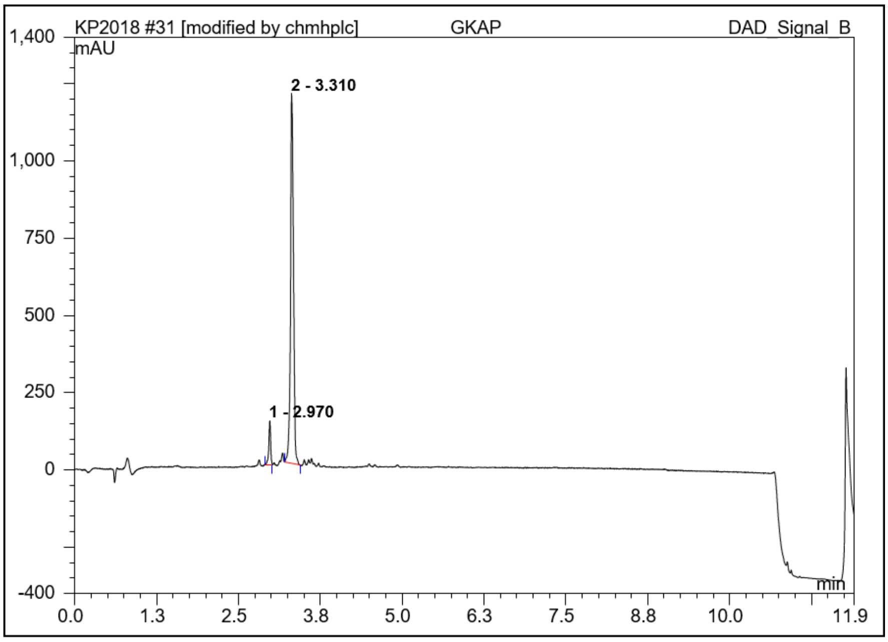

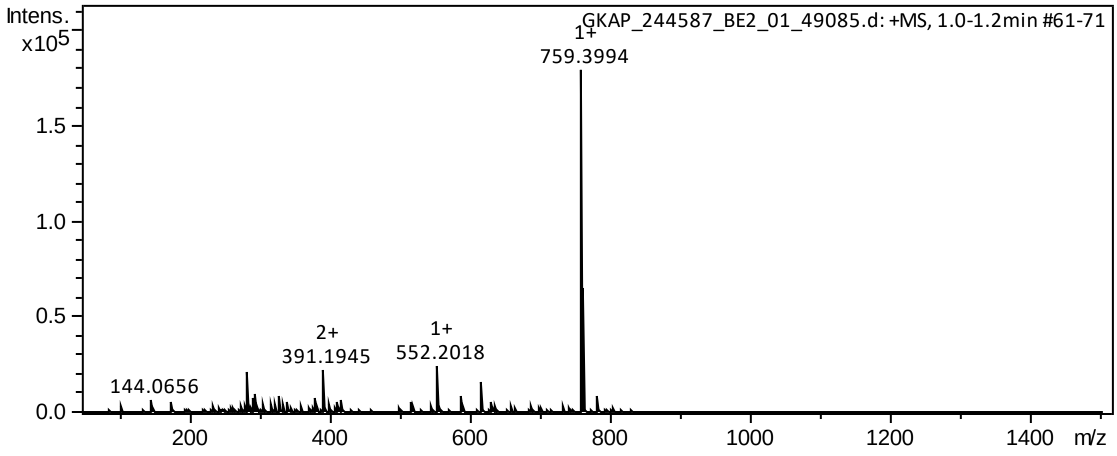

Ac-TRL-OH

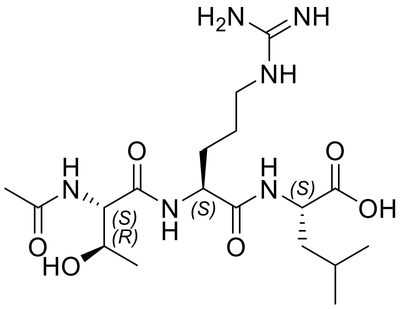

Chemical Formula: $C_{18}H_{34}N_6O_6$

Exact Mass: 430.2540

Expected $[M+H^+]^{1+}$	Measured $[M+H^+]^{1+}$
431.2613	431.2633

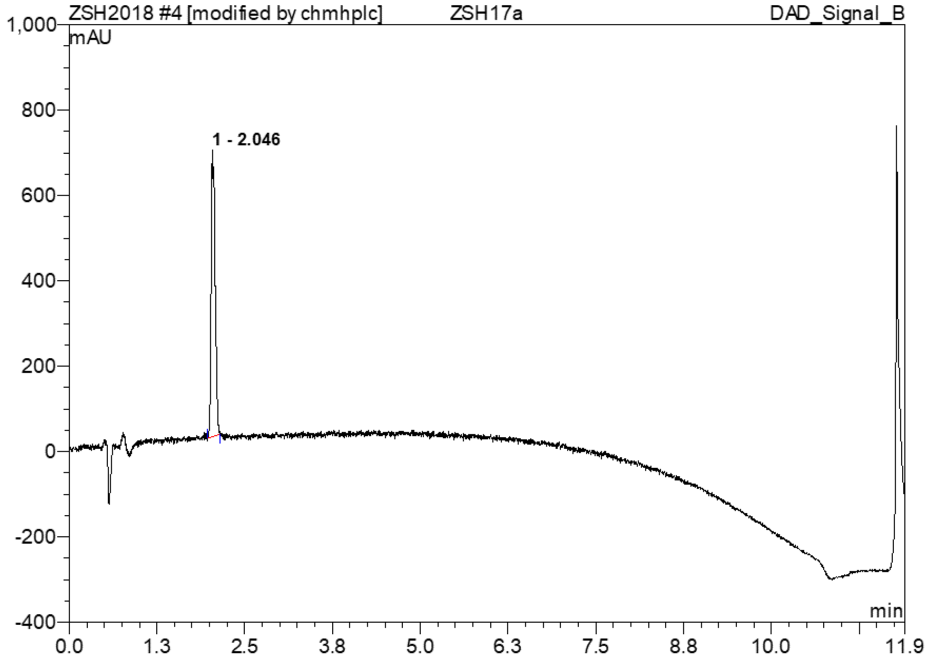

99+% purity

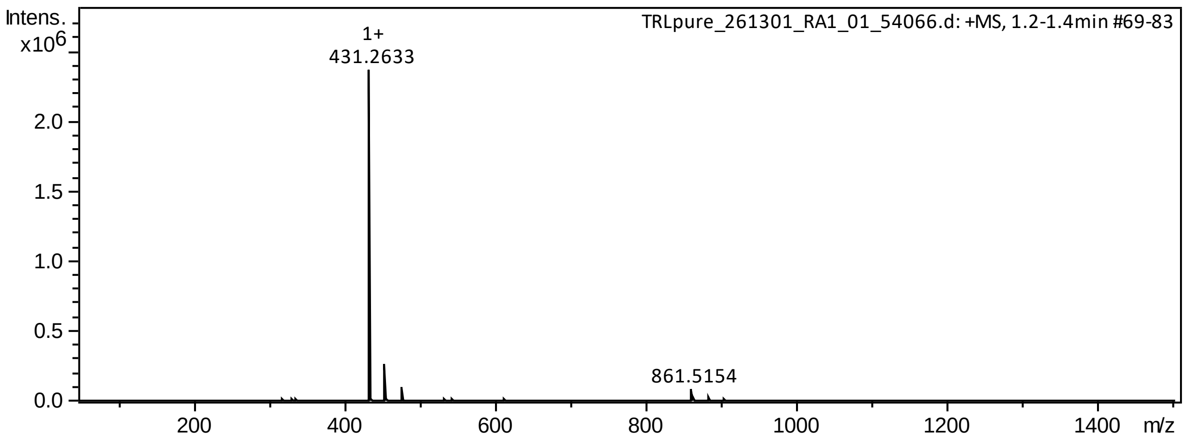

FITC-Ahx-TRL-OH

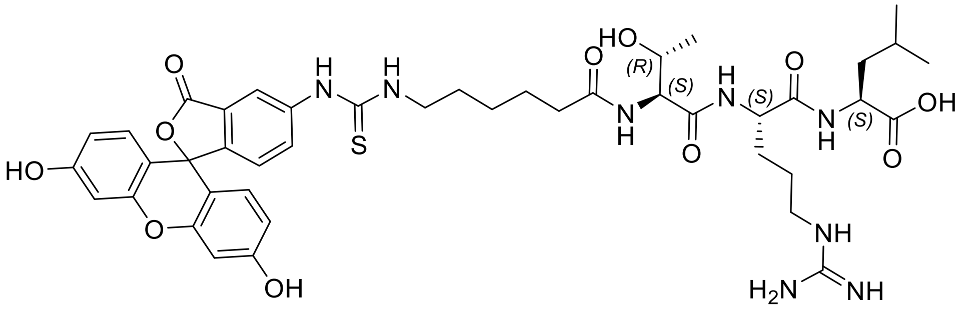

Chemical Formula: $C_{43}H_{54}N_8O_{11}S$

Exact Mass: 890.3633

Expected $[M+H^+]^{1+}$	Measured $[M+H^+]^{1+}$	Expected $[M+2H^+]^{2+}$	Measured $[M+2H^+]^{2+}$
891.3706	891.3716	446.1889	446.1910

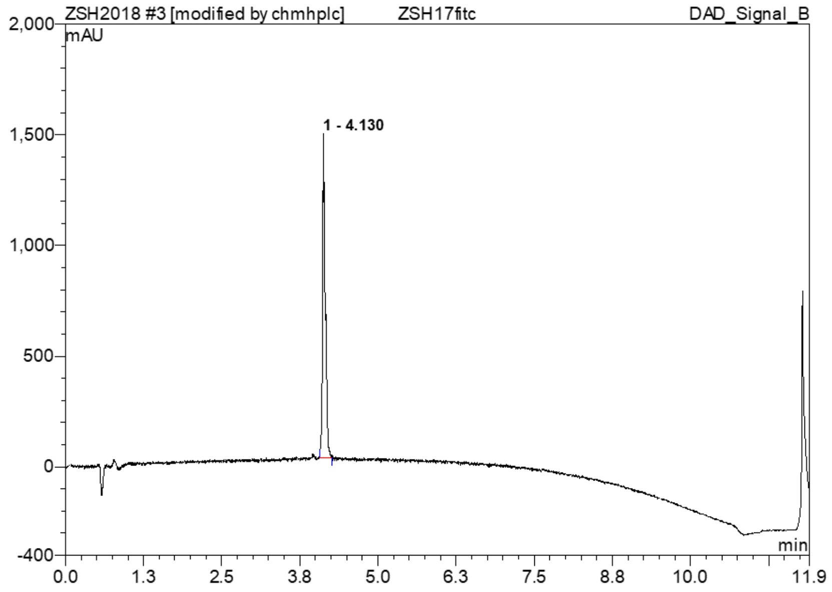

99+ % purity

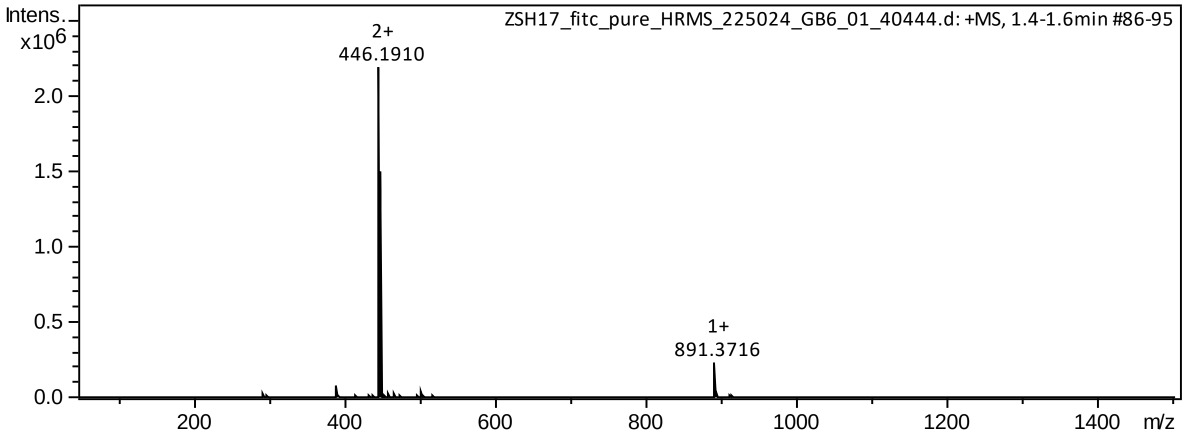

4-(N'-tert-butoxycarbonyl-hydrazino)-4-oxo-butyric acid

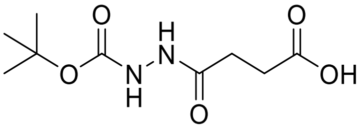

1.0 g (10 mmol) of succinic anhydride and 1.32 g (10 mmol) of *tert*-butyl carbazate were dissolved in 20 mL water. Within 15 min the solution became clear. The reaction mixture was lyophilized and the product was used in SPPS without any further purification. ^{1}H NMR (DMSO-d_6, 400.13 MHz): _ (ppm) = 1.39 (s, 9H, C(CH_3)$_3$), 2.32 (t, 2H, CH_2COOH), 2.43 (t, 2H, CH_2C=ONH), 8.69 (s, 1H, N*H*-*t*Boc), 9.52 (s, 1H, N*H*C=OCH_2); ESI-MS calcd. for $C_9H_{16}N_2O_5$ ([M-1H]$^-$): 231.0986, found 231.0980

4-(N'-tert-butoxycarbonyl-hydrazino)-4-oxo-propionic acid

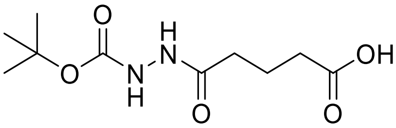

1.1 g (10 mmol) of glutaric anhydride and 1.32 g (10 mmol) of *tert*-butyl carbazate were dissolved in 20 mL water. Within 15 min the solution became clear. The reaction mixture was lyophilized and the product was used in SPPS without any further purification. ^{1}H NMR (DMSO-d_6, 400.13 MHz): (ppm) = 1.39 (s, 9H, C(CH_3)$_3$), 1.71 (quint, 2H, CH_2C$H_2$$CH_2$), 2.09 (t, 2H, C$H_2$COOH), 2.23 (t, 2H, C$H_2$C=ONH), 8.64 (s, 1H, N*H*-*t*Boc), 9.47 (s, 1H, N*H*C=OCH_2); ESI-MS calcd. for $C_{10}H_{18}N_2O_5$ ([M-1H]$^-$): 245.1143, found 245.1138.

1-A001

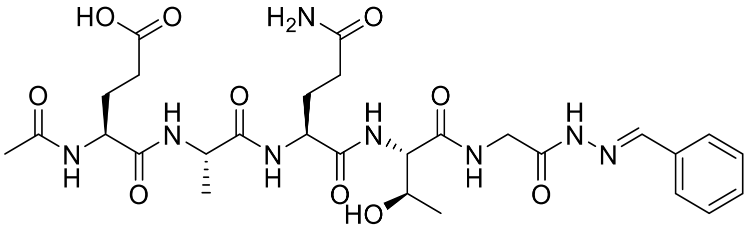

Chemical Formula: $C_{28}H_{40}N_8O_{10}$

Exact Mass: 648.2867

Expected $[M+H^+]^{1+}$	Measured $[M+H^+]^{1+}$	Expected $[M+Na^+]^{1+}$	Measured $[M+Na^+]^{1+}$
649.2940 (hydazone)	649.2938	671.2766	671.2756
561.2627 (hydrazide)	561.2621	583.2449	583.2439

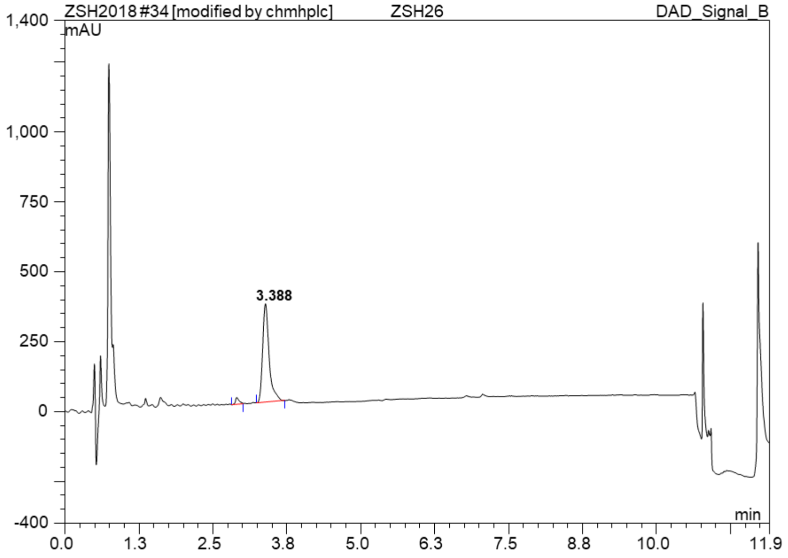

96.2 % purity

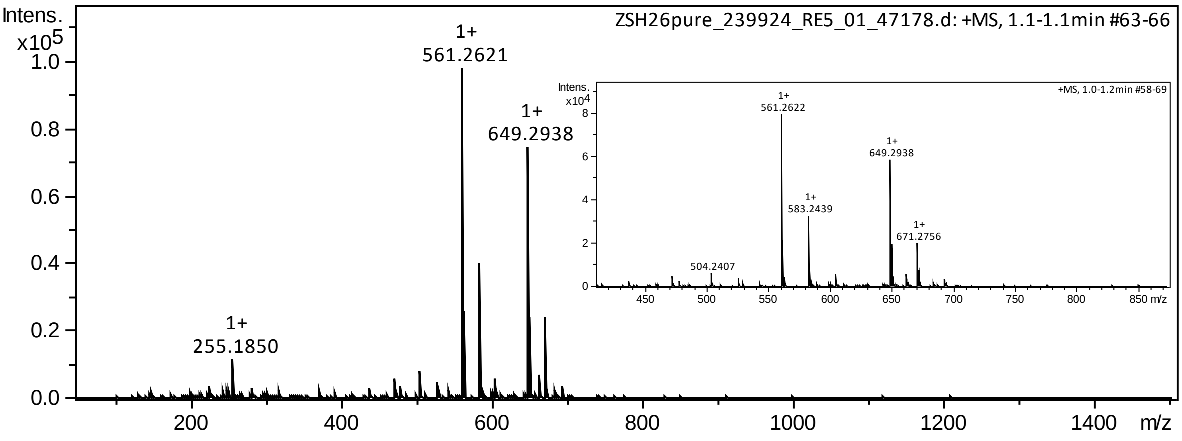

2-A001

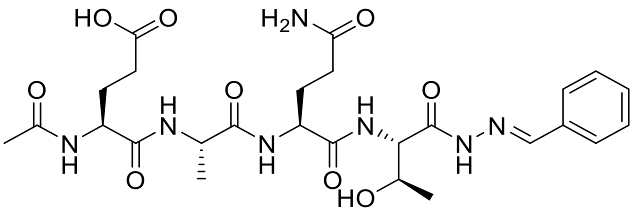

Chemical Formula: $C_{26}H_{37}N_7O_9$

Exact Mass: 591.2653

Expected $[M+H^+]^{1+}$	Measured $[M+H^+]^{1+}$	Expected $[M+Na^+]^{1+}$	Measured $[M+Na^+]^{1+}$
592.2726	592.2721	614.2554	614.2539

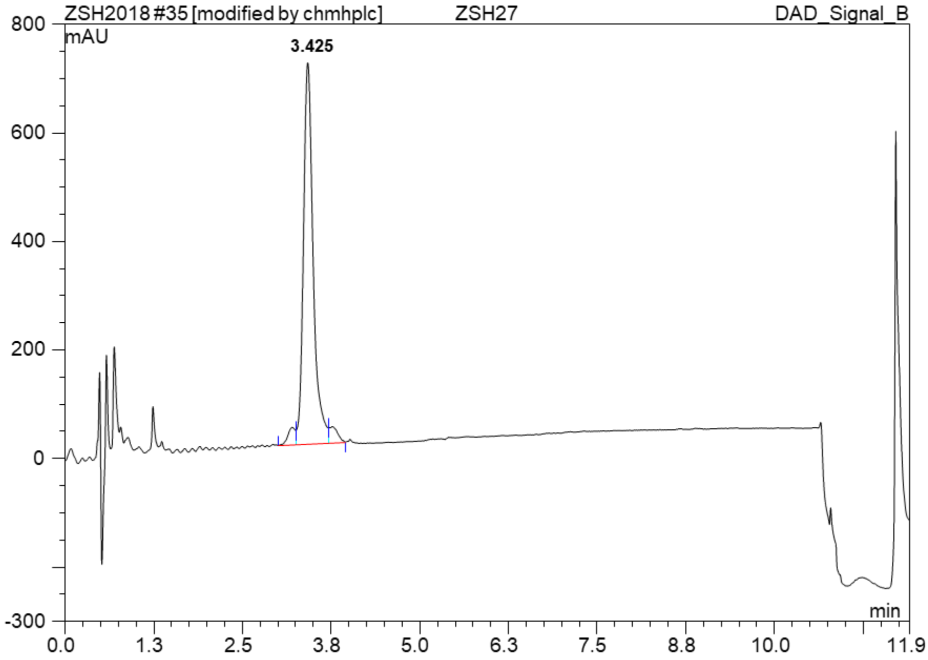

92.7 % purity

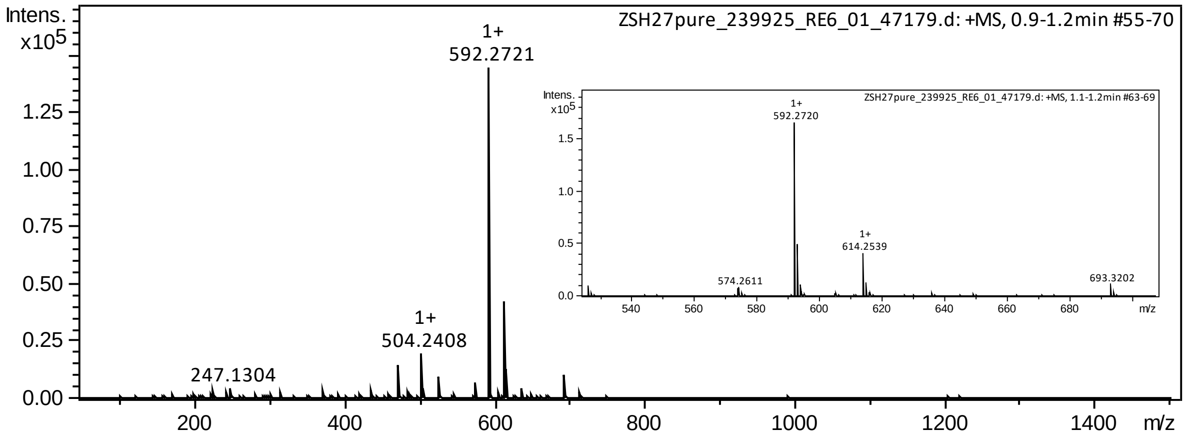

3-A001

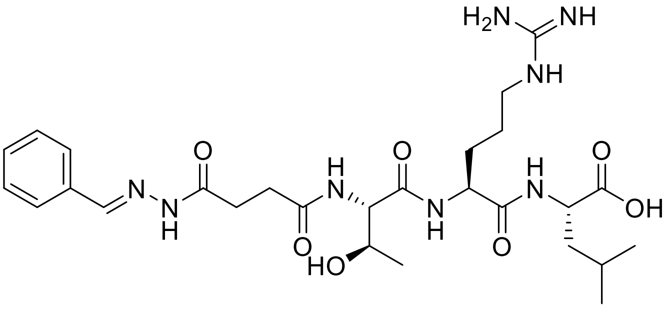

Chemical Formula: $C_{27}H_{42}N_8O_7$

Exact Mass: 590.3176

Expected $[M+H^+]^{1+}$	Measured $[M+H^+]^{1+}$	Expected $[M+H^+]^{2+}$	Measured $[M+2H^+]^{2+}$
591.3249	591.3251	296.1661	296.1663

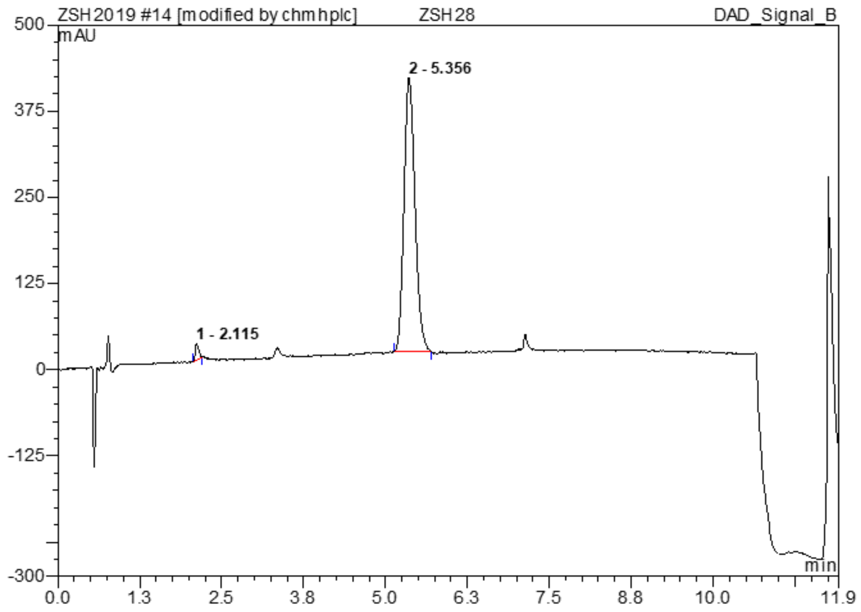

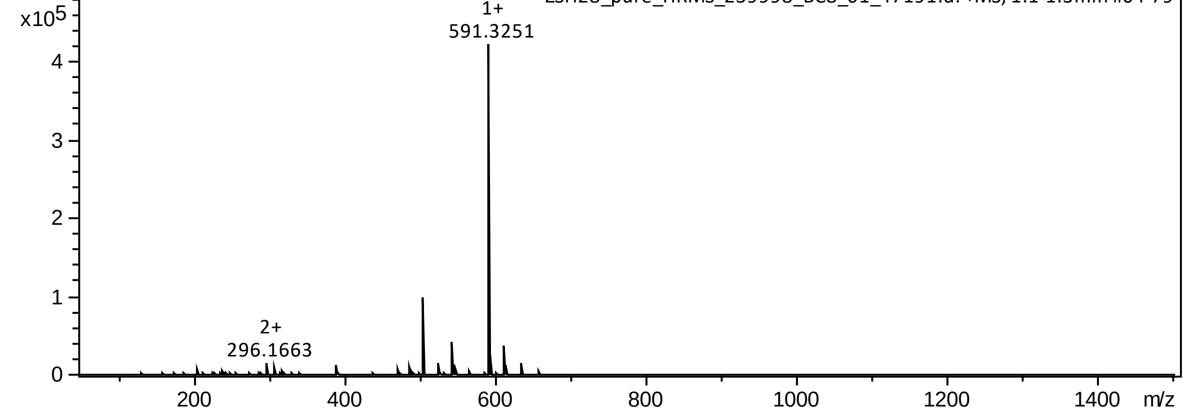

4-A001

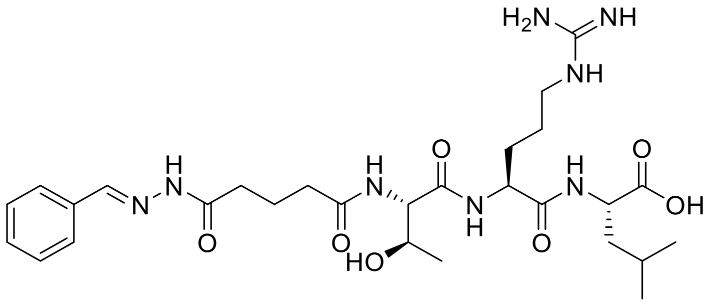

Chemical Formula: $C_{28}H_{44}N_8O_7$

Exact Mass: 604.3333

Expected $[M+H^+]^{1+}$	Measured $[M+H^+]^{1+}$	Expected $[M+H^++Na^+]^{2+}$	Measured $[M+H^++Na^+]^{2+}$
605.3406	605.3408	314.1665	314.1652

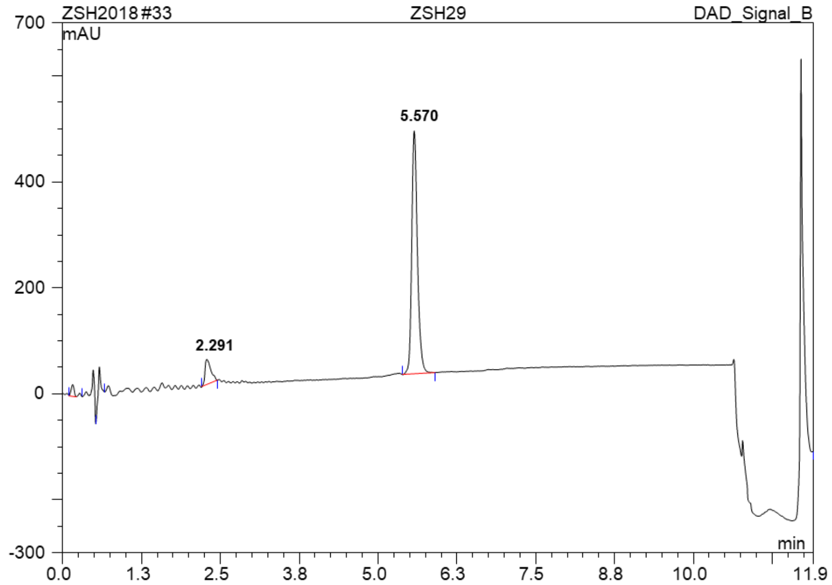

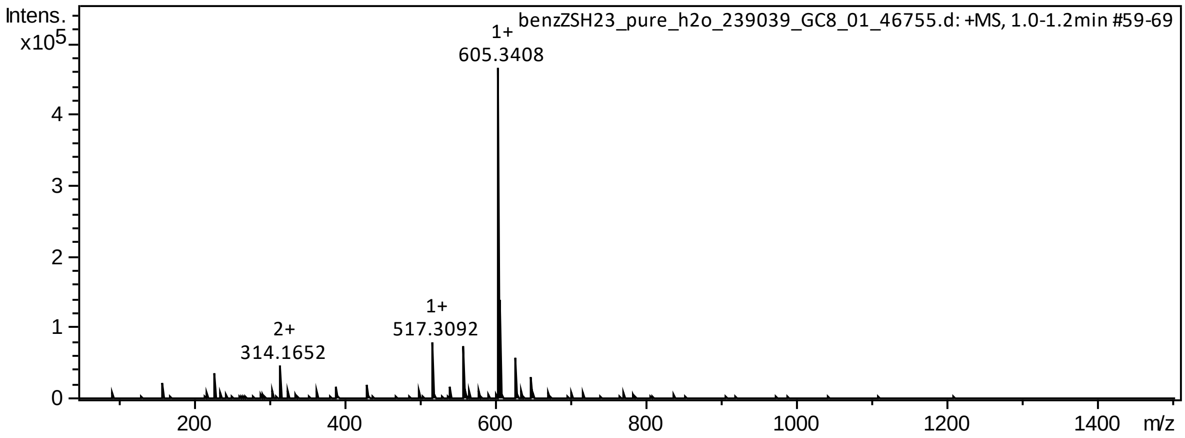

3-A047

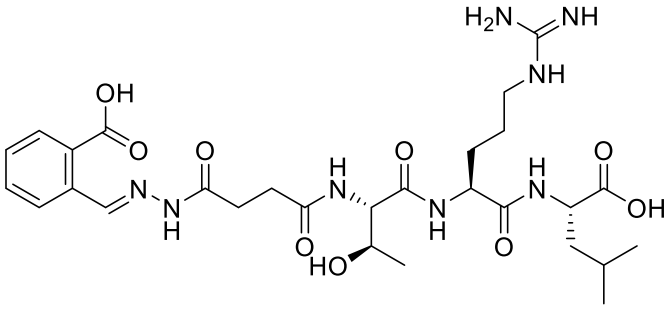

Chemical Formula: $C_{28}H_{42}N_8O_9$

Exact Mass: 634.3075

Expected $[M+H^+]^{1+}$	Measured $[M+H^+]^{1+}$
635.3148	635.3138

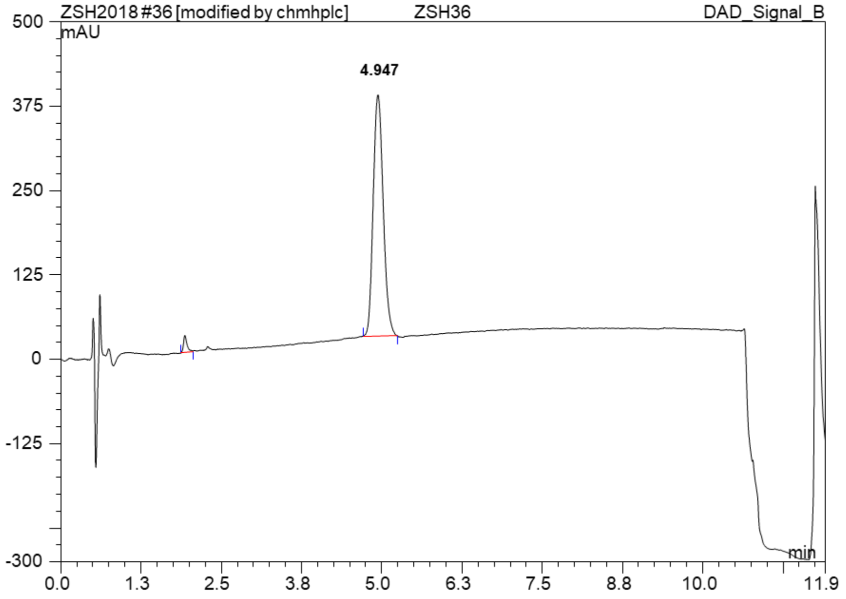

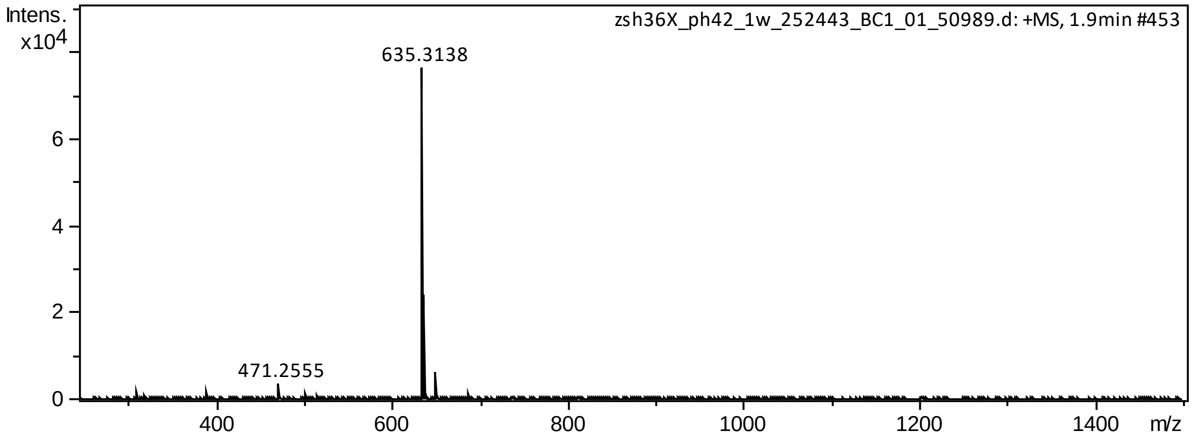

4-A047

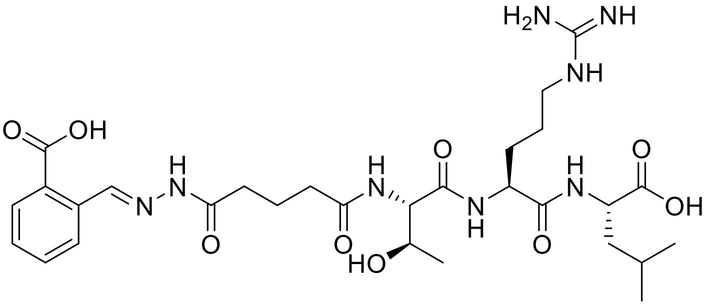

Chemical Formula: $C_{29}H_{44}N_8O_9$

Exact Mass: 648.3231

Expected $[M+H^+]^{1+}$	Measured $[M+H^+]^{1+}$
649.3304	649.3310

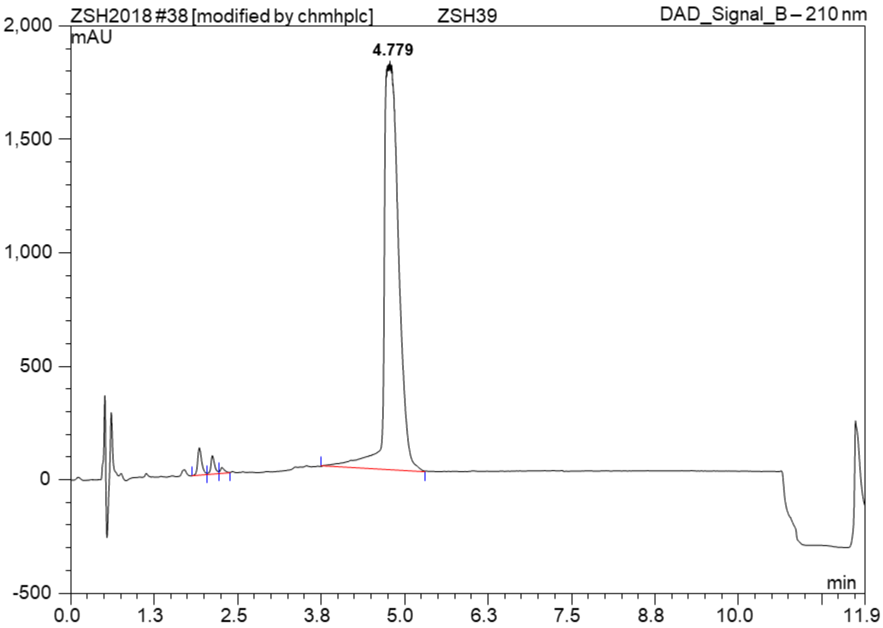

HPLC chromatogram λ = 210 nM

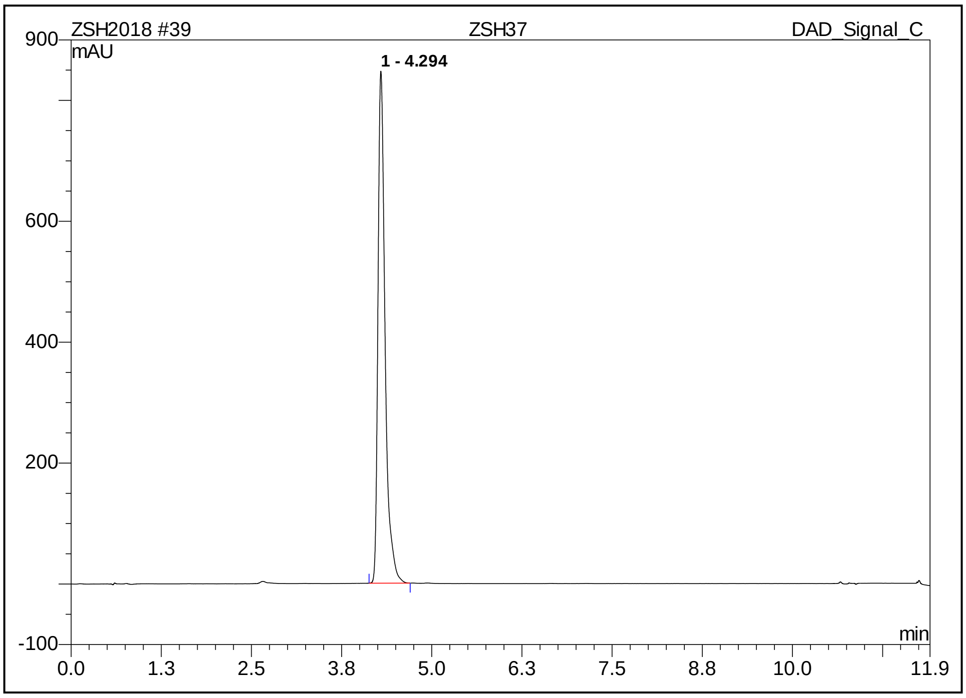

HPLC chromatogram λ = 280 nM

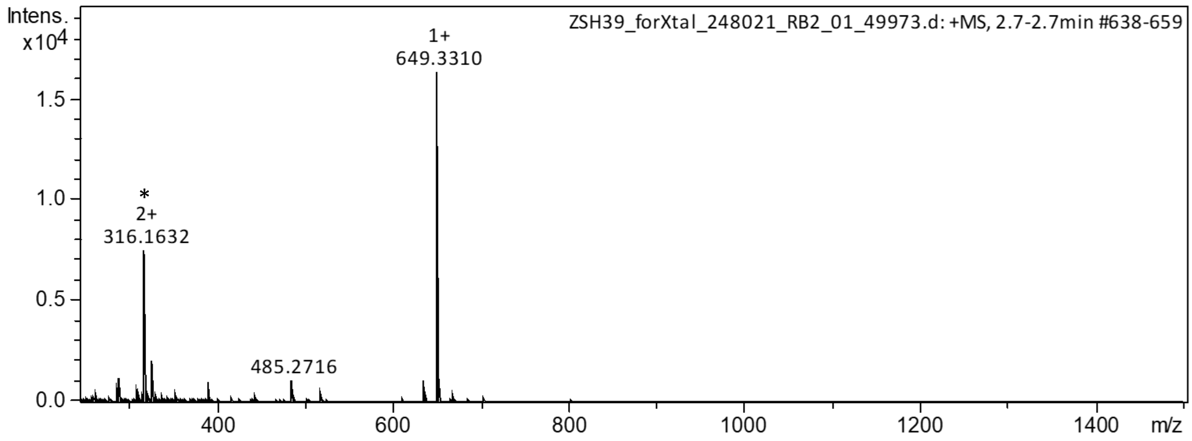

* $[M\text{-}H_2O+2H^+]^{2+}$ = 316.1632

3-A048

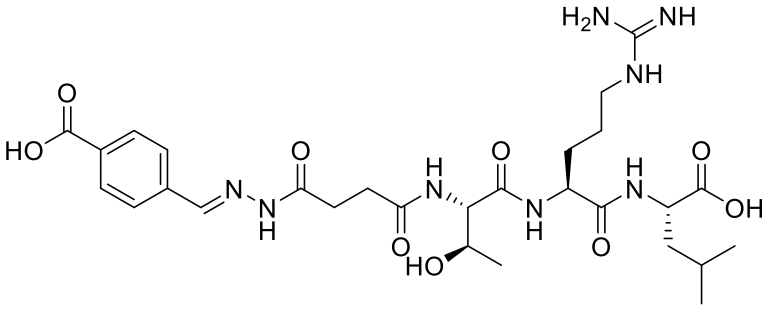

Chemical Formula: $C_{28}H_{42}N_8O_9$

Exact Mass: 634.3075

Expected $[M+H^+]^{1+}$	Measured $[M+H^+]^{1+}$
635.3148	635.3139

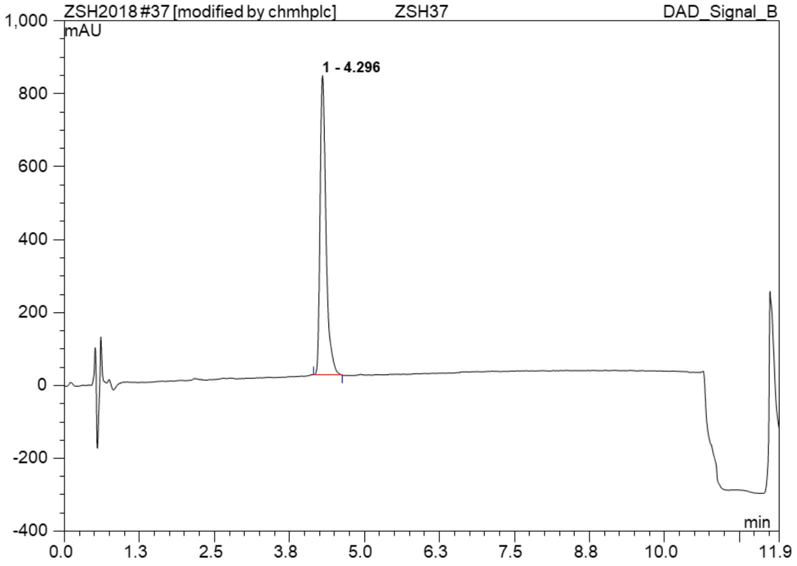

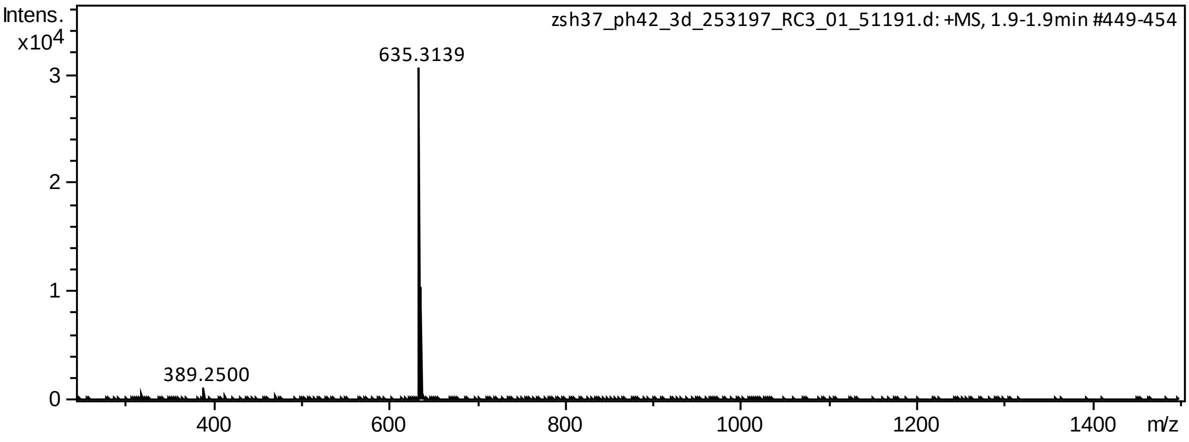

4-A048

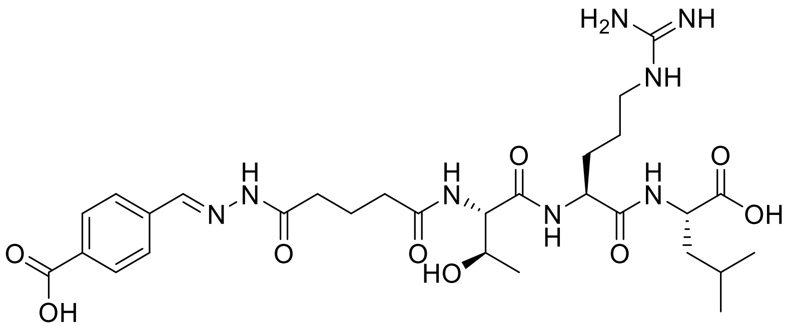

Chemical Formula: $C_{29}H_{44}N_8O_9$

Exact Mass: 648.3231

Expected [M+H+]1+	Measured [M+H+]1+
649.3304	649.3293

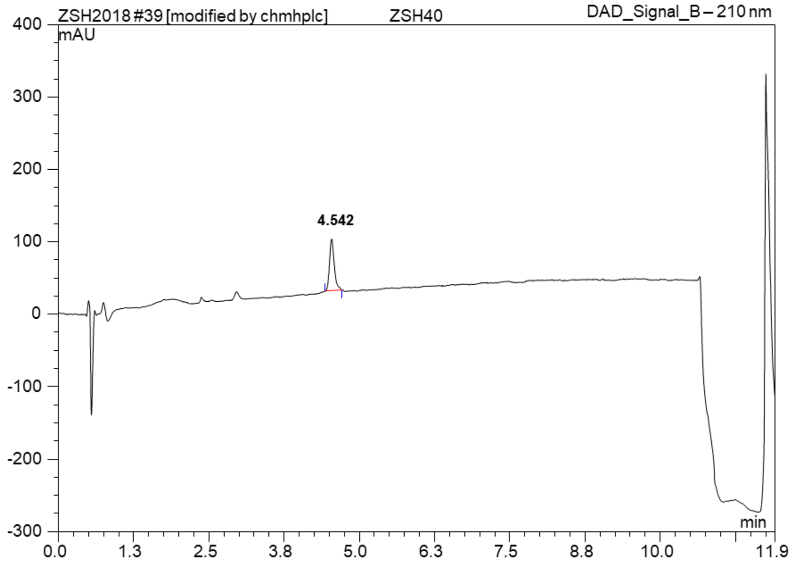

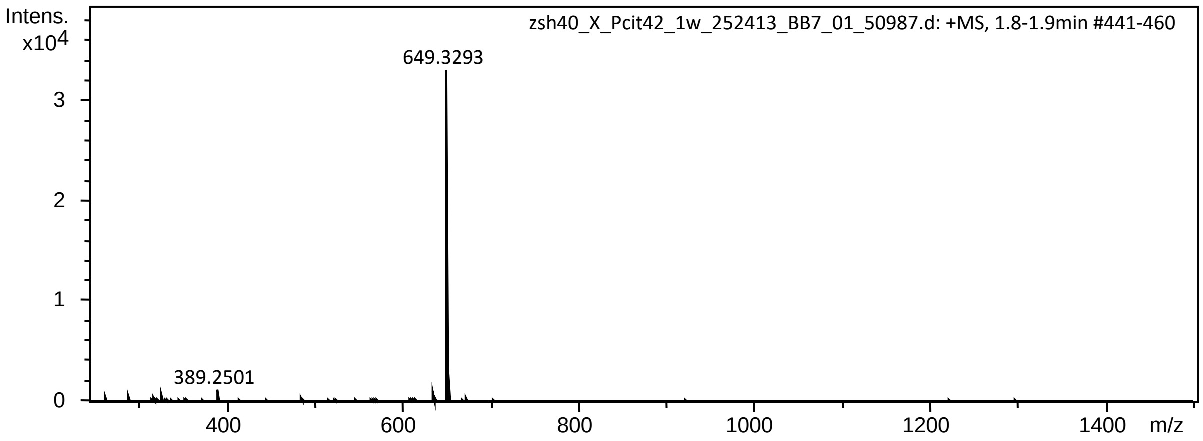

3-A144

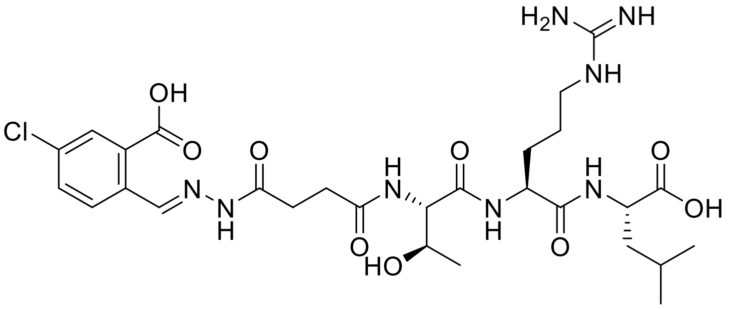

Chemical Formula: $C_{28}H_{41}ClN_8O_9$

Exact Mass: 668.2685

Expected $[M+H^+]^{1+}$	Measured $[M+H^+]^{1+}$
669.2758	669.2756

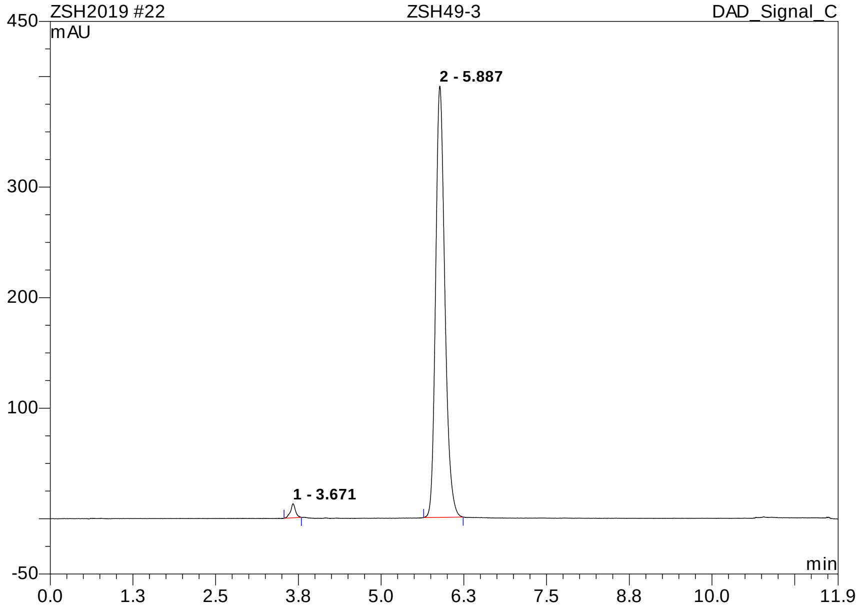

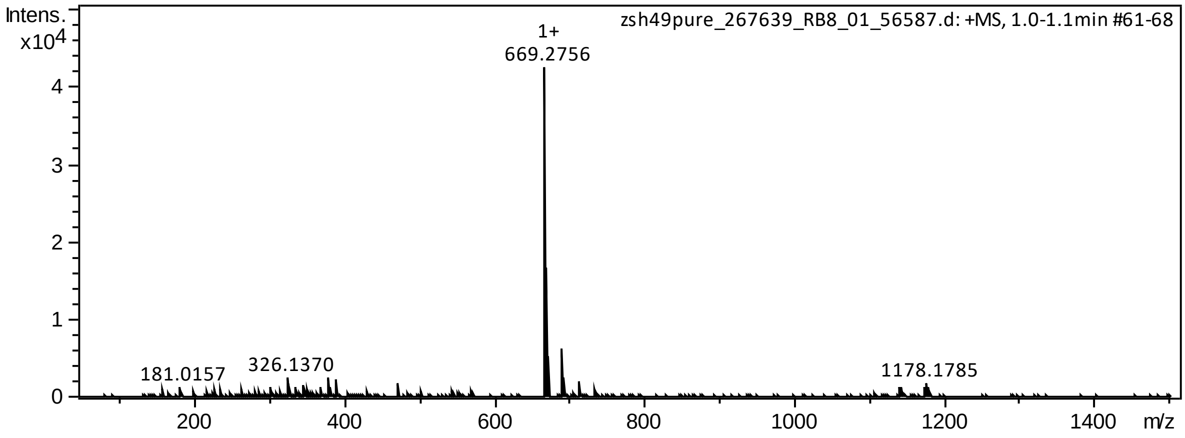

Purity: 98.26 %

4-A144

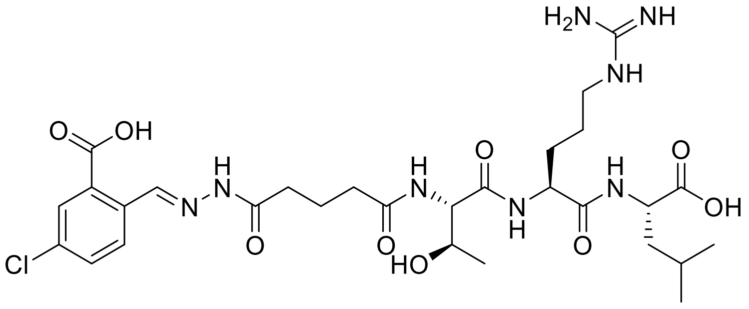

Chemical Formula: $C_{29}H_{43}ClN_8O_9$

Exact Mass: 682.2842

Expected $[M+H^+]^{1+}$	Measured $[M+H^+]^{1+}$
683.2915	683.2909

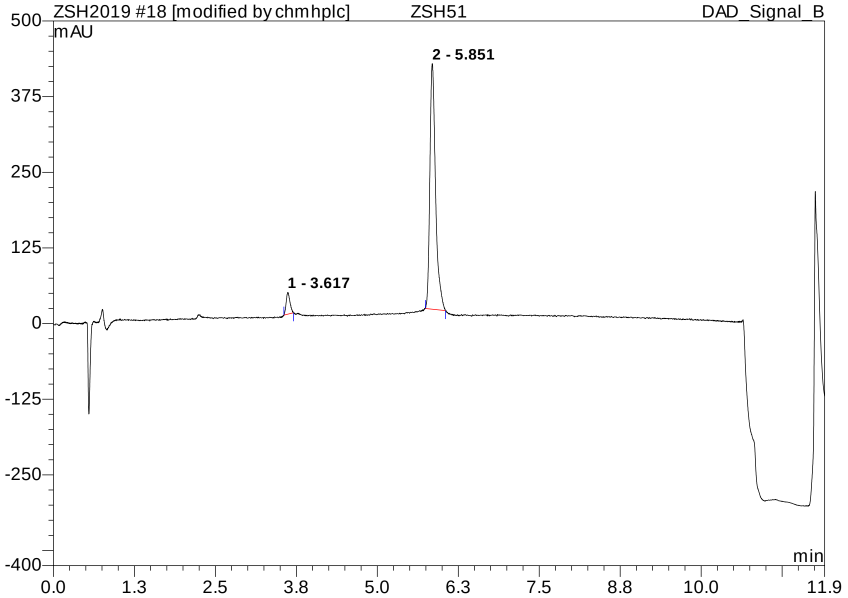

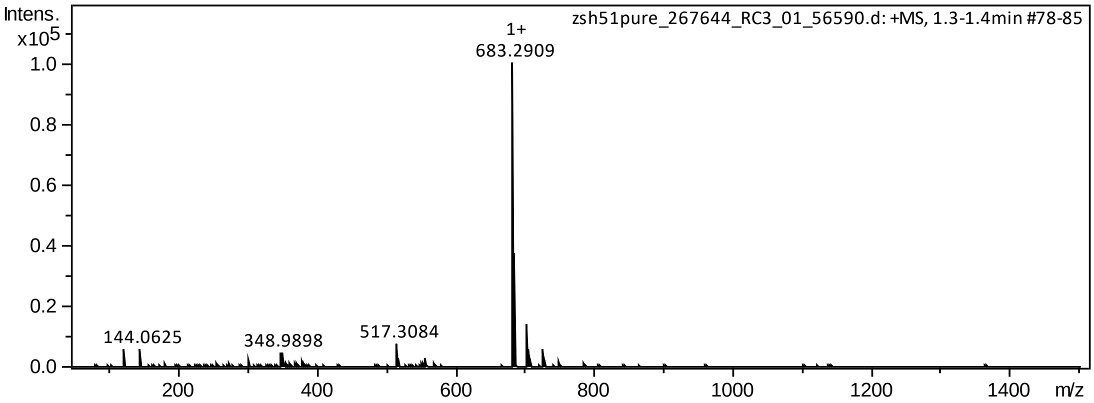

Purity : 94.48 %

3-A161

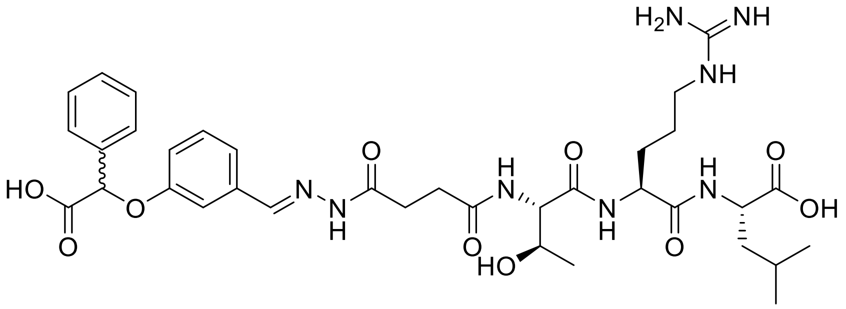

Chemical Formula: $C_{35}H_{48}N_8O_{10}$

Exact Mass: 740.3493Expected $[M+H^+]^{1+}$	Measured $[M+H^+]^{1+}$	Expected $[M+2H^+]^{2+}$	Measured $[M+2H^+]^{2+}$
741.3566	741.3563	371.1819	371.1821

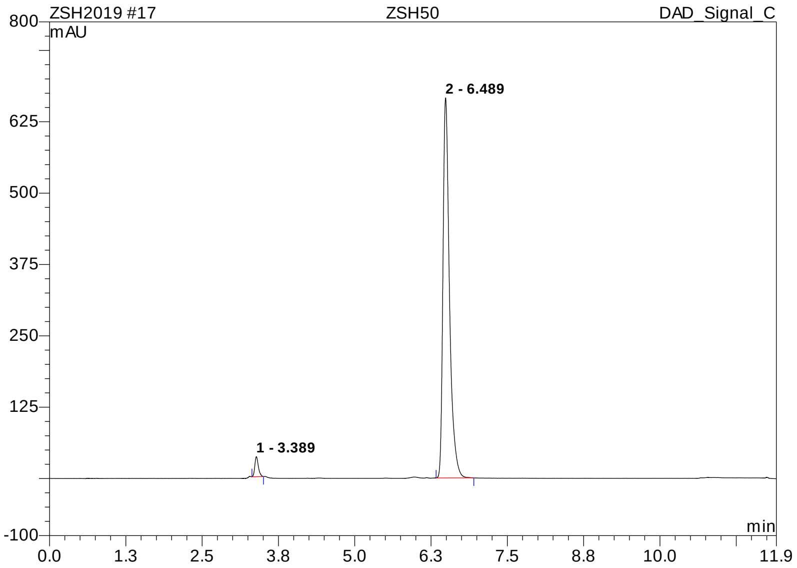

Note, that the absolute configuration and enantiomeric purity of A161 is unknown, a single peak in the HPLC chromatogram might indicate a single diastereoisomer or inseparable stereoisomers.

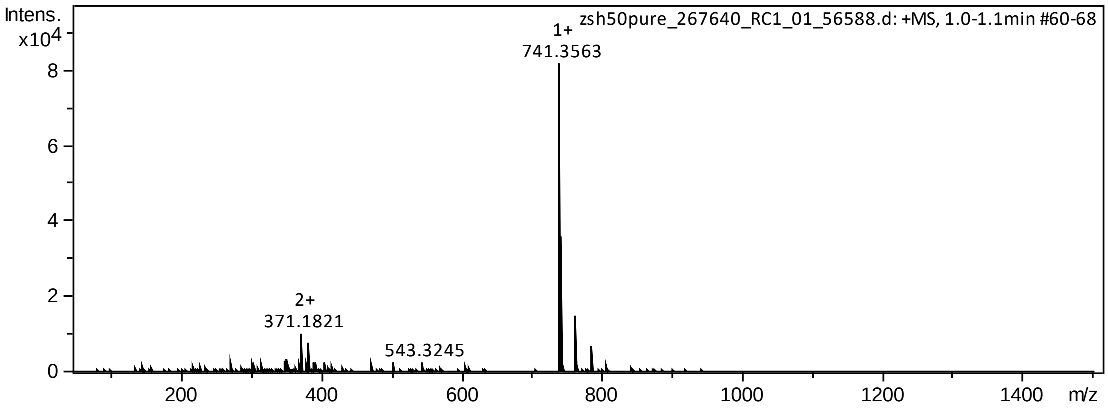

Purity: 97.22%

4-A161

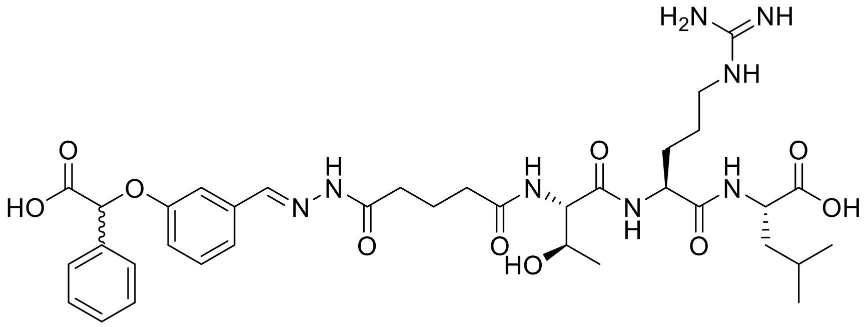

Chemical Formula: $C_{36}H_{50}N_8O_{10}$

Exact Mass: 754.3650Expected $[M+H^+]^{1+}$	Measured $[M+H^+]^{1+}$	Expected $[M+H^++Na^+]^{2+}$	Measured $[M+H^++Na^+]^{2+}$
755.3723	755.3723	389.1815	389.1802

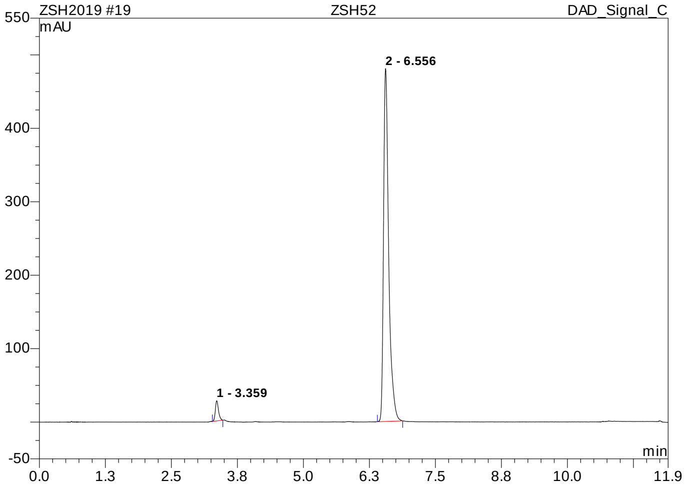

Note, that the absolute configuration and enantiomeric purity of A161 is unknown, a single peak in the HPLC chromatogram might indicate a single diastereoisomer or inseparable stereoisomers.

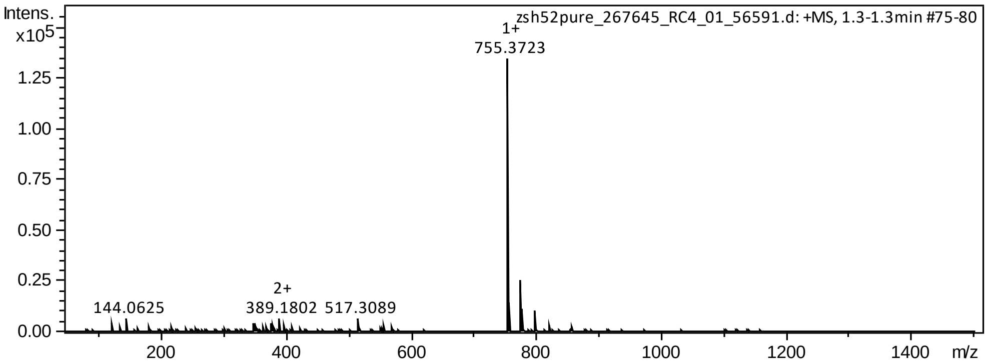

Purity: 96.6 %

2.11. References

(1) Sander, T.; Freyss, J.; Von Korff, M.; Rufener, C. DataWarrior: An Open-Source Program for Chemistry Aware Data Visualization and Analysis. *J. Chem. Inf. Model.* **2015**, *55* (2), 460–473.

(2) Im, Y. J.; Lee, J. H.; Park, S. H.; Park, S. J.; Rho, S. H.; Kang, G. B.; Kim, E.; Eom, S. H. Crystal Structure of the Shank PDZ-Ligand Complex Reveals a Class I PDZ Interaction and a Novel PDZ-PDZ Dimerization. *J. Biol. Chem.* **2003**, *278* (48), 48099–48104.

(3) Vogrig, A.; Dorr, L.; Bouzidi, N.; Boucherle, B.; Wattiez, A. S.; Cassier, E.; Vallon, G.; Ripoche, I.; Abrunhosa-Thomas, I.; Marin, P.; Nauton, L.; Thery, V.; Courteix, C.; Lian, L. Y.; Ducki, S. Structure-Based Design of PDZ Ligands as Inhibitors of 5-HT2A Receptor/PSD-95 PDZ1 Domain Interaction Possessing Anti-Hyperalgesic Activity. *ACS Chem. Biol.* **2013**, *8* (10), 2209–2216.

(4) Miles, J. A.; Yeo, D. J.; Rowell, P.; Rodriguez-Marin, S.; Pask, C. M.; Warriner, S. L.; Edwards, T. A.; Wilson, A. J. Hydrocarbon Constrained Peptides-Understanding Preorganisation and Binding Affinity. *Chem. Sci.* **2016**, *7* (6), 3694–3702.

(5) Winter, G. Xia2: An Expert System for Macromolecular Crystallography Data Reduction. *J. Appl. Crystallogr.* **2010**, *43* (1), 186–190.

(6) Winter, G.; Waterman, D. G.; Parkhurst, J. M.; Brewster, A. S.; Gildea, R. J.; Gerstel, M.; Fuentes-Montero, L.; Vollmar, M.; Michels-Clark, T.; Young, I. D.; Sauter, N. K.; Evans, G. DIALS: Implementation and Evaluation of a New Integration Package. *Acta Crystallogr. Sect. D Struct. Biol.* **2018**, *74* (2), 85–97.

(7) Evans, P. Scaling and Assessment of Data Quality. *Acta Crystallogr. Sect. D Biol. Crystallogr.* **2006**, *62* (1), 72–82.

(8) Evans, P. R.; Murshudov, G. N. How Good Are My Data and What Is the Resolution? *Acta Crystallogr. Sect. D Biol. Crystallogr.* **2013**, *69* (7), 1204–1214.

(9) Vonrhein, C.; Flensburg, C.; Keller, P.; Sharff, A.; Smart, O.; Paciorek, W.; Womack, T.; Bricogne, G. Data Processing and Analysis with the AutoPROC Toolbox. *Acta Crystallogr. Sect. D Biol. Crystallogr.* **2011**, *67* (4), 293–302.

(10) Kabsch, W. Xds. *Acta Crystallogr. Sect. D Biol. Crystallogr.* **2010**, *66* (2), 125–132.

(11) Tickle, I.; Flensburg, C.; Keller, P.; Paciorek, W.; Cambridge, A. S.-; Kingdom, U.; 2018, undefined. Staraniso.

(12) McCoy, A. J.; Grosse-Kunstleve, R. W.; Adams, P. D.; Winn, M. D.; Storoni, L. C.; Read, R. J. Phaser Crystallographic Software. *J. Appl. Crystallogr.* **2007**, *40* (4), 658–674.

(13) Vagin, A. A.; Steiner, R. A.; Lebedev, A. A.; Potterton, L.; McNicholas, S.; Long, F.; Murshudov, G. N. REFMAC5 Dictionary: Organization of Prior Chemical Knowledge and Guidelines for Its Use. *Acta Crystallogr. Sect. D Biol. Crystallogr.* **2004**, *60* (12 I), 2184–2195.

(14) Emsley, P.; Lohkamp, B.; Scott, W. G.; Cowtan, K. Features and Development of Coot. *Acta Crystallogr. Sect. D Biol. Crystallogr.* **2010**, *66* (4), 486–501.

(15) Winn, M. D.; Ballard, C. C.; Cowtan, K. D.; Dodson, E. J.; Emsley, P.; Evans, P. R.; Keegan, R. M.; Krissinel, E. B.; Leslie, A. G. W.; McCoy, A.; McNicholas, S. J.; Murshudov, G. N.; Pannu, N. S.; Potterton, E. A.; Powell, H. R.; Read, R. J.; Vagin, A.; Wilson, K. S. Overview of the CCP4 Suite and Current Developments. *Acta Crystallogr. Sect. D Biol. Crystallogr.*

2011, *67* (4), 235–242.

(16) Afonine, P. V.; Grosse-Kunstleve, R. W.; Echols, N.; Headd, J. J.; Moriarty, N. W.; Mustyakimov, M.; Terwilliger, T. C.; Urzhumtsev, A.; Zwart, P. H.; Adams, P. D. Towards Automated Crystallographic Structure Refinement with Phenix.Refine. *Acta Crystallogr. Sect. D Biol. Crystallogr.* **2012**, *68* (4), 352–367.

(17) Chen, V. B.; Arendall, W. B.; Headd, J. J.; Keedy, D. A.; Immormino, R. M.; Kapral, G. J.; Murray, L. W.; Richardson, J. S.; Richardson, D. C. MolProbity: All-Atom Structure Validation for Macromolecular Crystallography. *Acta Crystallogr. Sect. D Biol. Crystallogr.* **2010**, *66* (1), 12–21.

(18) Long, F.; Nicholls, R. A.; Emsley, P.; Gražulis, S.; Merkys, A.; Vaitkus, A.; Murshudov, G. N. AceDRG: A Stereochemical Description Generator for Ligands. *Acta Crystallogr. Sect. D Struct. Biol.* **2017**, *73* (2), 112–122.

(19) Da Silva, A. W. S.; Vranken, W. F. ACPYPE-Antechamber Python Parser Interface. *BMC Res. notes* **2012**, *5* (1), 367.

(20) Goddard, T.; reference, D. K.-T. is no corresponding record for this; 2008, undefined. Sparky 3. University of California, San Francisco, USA.

(21) Kay, L. E.; Torchia, D. A.; Bax, A. Backbone Dynamics of Proteins As Studied by 15N Inverse Detected Heteronuclear NMR Spectroscopy: Application to Staphylococcal Nuclease. *Biochemistry* **1989**, *28* (23), 8972–8979.
